# Study of the UV Light Conversion of Feruloyl Amides from *Portulaca oleracea* and Their Inhibitory Effect on IL-6-Induced STAT3 Activation

**DOI:** 10.3390/molecules21070865

**Published:** 2016-06-30

**Authors:** Joo Tae Hwang, Yesol Kim, Hyun-Jae Jang, Hyun-Mee Oh, Chi-Hwan Lim, Seung Woong Lee, Mun-Chual Rho

**Affiliations:** 1 Natural Product Research Center, Korea Research Institute of Bioscience and Biotechnology, 181 Ipsin-gil, Jeongeup-si, Jeonbuk 56212, Korea; jthwang@kribb.re.kr (J.T.H.); yesolkim@kribb.re.kr (Y.K.); water815@kribb.re.kr (H.-J.J.); ohhm@kribb.re.kr (H.-M.O.); 2 College of Agriculture and Life Sciences, Chungnam National University, 99 Daehak-ro, Yuseong-gu, Daejeon 300-764, Korea; chlim@cnu.ac.kr

**Keywords:** *Portulaca oleaceae* L, feruloyl amides, IL-6, STAT3, conversion study

## Abstract

Two new feruloyl amides, *N*-*cis*-hibiscusamide (**5**) and (7′*S*)-*N*-*cis*-feruloylnormetanephrine (**9**), and eight known feruloyl amides were isolated from *Portulaca oleracea* L. and the geometric conversion of the ten isolated feruloyl amides by UV light was verified. The structures of the feruloyl amides were determined based on spectroscopic data and comparison with literature data. The NMR data revealed that the structures of the isolated compounds showed *cis*/*trans*-isomerization under normal laboratory light conditions. Therefore, *cis* and *trans*-isomers of feruloyl amides were evaluated for their convertibility and stability by UV light of a wavelength of 254 nm. After 96 h of UV light exposure, 23.2%–35.0% of the *cis* and *trans*-isomers were converted to *trans*-isomers. Long-term stability tests did not show any significant changes. Among all compounds and conversion mixtures collected, compound **6** exhibited the strongest inhibition of IL-6-induced STAT3 activation in Hep3B cells, with an IC_50_ value of 0.2 μM. This study is the first verification of the conversion rates and an equilibrium ratio of feruloyl amides. These results indicate that this natural material might provide useful information for the treatment of various diseases involving IL-6 and STAT3.

## 1. Introduction

*P. oleracea* is an annual plant widely distributed in the Middle East, Central Europe, America, Australia and Asia [1,2]. It is generally more tolerant than other crops to saline soil and to drought, high temperature, and low nutrient conditions [3,4]. The plant’s strong adaptability might inhibit major crops from growing in some regions, and as such, *P. oleracea* is also considered a weed. However, the stems and leaves of the plant are edible, with a salty and sour taste. These parts of the plant are commonly used for salads or are cooked like spinach in many countries [2,5]. Additionally, the plant leaves and stems have long been used as traditional medicines for diuretic, febrifuge, antispasmodic, antiseptic and vermifuge purposes [6]. In particular, recent studies have demonstrated the nutritional and pharmaceutical importance of *P. oleracea*. Chemical constituents such as omega-3-fatty acids, vitamins and β-carotene are present at high concentrations in this plant [4,7]. Flavonoids, coumarins, monoterpene glycosides and alkaloids have also been observed [6]. The extensive pharmacological activities of these compounds, including analgesic, skeletal muscle-relaxing, anti-ulcerogenic, anti-hypoxic, anti-aging, anti-inflammatory and anti-oxidative activities, have been previously studied [1,8,9,10].

Interleukin-6 (IL-6) is an inflammatory cytokine. The levels of IL-6 are increased in several pathological conditions of inflammation and other diseases such as carcinomatous cachexia, multiple myeloma, rheumarthritis and hypercalcemia [11,12,13,14]. IL-6 activates signal transducer and activator of transcription 3 (STAT3), leading to an increase of various inflammatory factors such as tumor necrosis factor alpha (TNF-α), transforming growth factor beta 1 (TGF-β1), and interleukin-1 beta (IL-1β) [15]. Increased levels of inflammatory factors have been reported to promote human inflammatory diseases [16]. Therefore, many studies have aimed to identify candidate treatments inhibiting the activation of IL-6 or STAT3.

**Figure 1:**
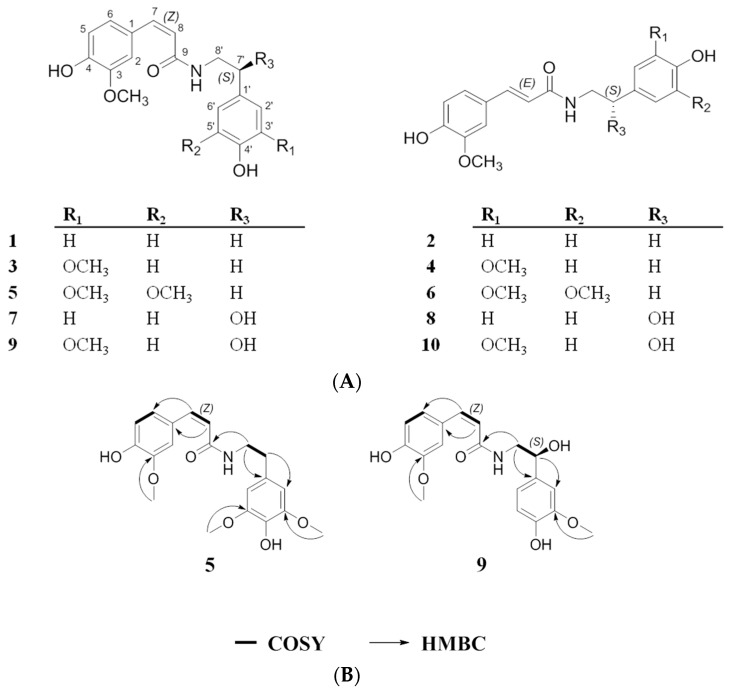
Structures of compounds **1**–**10** isolated from *Portulaca oleracea* (**A**); ^1^H-^1^H COSY and HMBC correlations for compounds **5** and **9** (**B**).

In our search for inhibitors of IL-6/STAT3 from edible plants, the 95% EtOH extract of the entire *P. oleracea* plant showed inhibitory activity on IL-6-induced STAT3 activation in Hep3B cells. Ten feruloyl amides **1**–**10** were next purified from the *P. oleracea* 95% EtOH extract (Figure 1A), and their chemical structures were identified by the corresponding spectroscopic data. Herein, we describe the isolation and structural determination of these feruloyl amides, their conversion rates upon UV light exposure and their inhibitory effects on IL-6-induced STAT3 activation in Hep3B cells.

## 2. Results and Discussion

### 2.1. Structural Elucidation of the Isolated Compounds

The EtOH extract from *P. oleracea* inhibited STAT3 reporter gene activation by IL-6 in Hep3B cells (60 μg/mL: 101.1% ± 0.4%, 30 μg/mL: 91.0% ± 0.3% and 10 μg/mL: 33.1% ± 1.2% inhibition in triplicate experiments). The following ten *cis* and *trans*-feruloyl amide isomers were isolated from the EtOH extract: *N*-*cis*-feruloyltyramine (**1**), *N*-*trans*-feruloyltyramine (**2**), *N*-*cis*-feruloyl-3′-methoxy-tyramine (**3**), *N*-*trans*-feruloyl-3′-methoxytyramine (**4**) *N*-*cis*-hibiscusamide (**5**), *N*-*trans*-hibiscus-amide (**6**), (7′*S*)-*N*-*cis*-feruloyloctopamine (**7**), (7′*S*)-*N*-*trans*-feruloyloctopamine (**8**), (7′*S*)-*N*-*cis*-feruloylnormetanephrine (**9**) and (7′*S*)-*N*-*trans*-feruloylnormetanephrine (**10**). The structures of **1**, **2**, **3**, **4**, **7**, **8** and **10** were determined from ESI-MS, ^1^H- and ^13^C-NMR data and through comparisons with the literature [17,18,19,20,21,22,23,24,25].

Compound **5** was isolated as a dark brown oil. A molecular formula of C_20_H_23_NO_6_ was determined based on its HRESI-MS spectrum, which showed a quasi-molecular ion peak at *m*/*z* 396.1418 [M + Na]^+^. The UV spectrum displayed absorption peaks at 201, 223 and 314 nm. The IR spectrum suggested the presence in the molecule of OH and NH groups, as indicated by a band at 3332 (*br*) cm^−1^ and an amide carbonyl group band at 1650 cm^−1^. The obtained ^13^C-NMR data clearly revealed 20 resonance signals, which were classified as three methoxy, two methylene, seven methine, and eight quaternary carbons (Table 1). The ^1^H-NMR spectrum exhibited signals for two olefinic protons at δ_H_ 6.57 (1H, d, *J* = 13.2 Hz, H-7) and 5.78 (1H, d, *J* = 13.2 Hz, H-8); ABX aromatic protons at δ_H_ 7.34 (1H, d, *J* = 2.0 Hz, H-2), 6.90 (1H, dd, *J* = 8.0, 2.0 Hz, H-6), and 6.69 (1H, d, *J* = 8.0 Hz, H-5); meta-coupled aromatic protons at δ_H_ 6.45 (2H, *s*, H-2′ and H-6′); two methylene protons at δ_H_ 2.70 (2H, t, *J* = 7.2 Hz, H-7′) and 3.42 (2H, t, *J* = 7.6 Hz, H-8′); and three methoxy groups at δ_H_ 3.75 (6H, s, H-3-OCH_3_ and H-3′-OCH_3_), and 3.79 (3H, s, H-5′-OCH_3_). These proton and carbon assignments were further confirmed by detailed analyses of the ^1^H-^1^H COSY, HMQC and HMBC spectra (Figure 1B). In the ^1^H-^1^H COSY spectrum, a spin-spin coupling partner sequence of H-5/H-6, H-7/H-8 and H-7′/H-8′ was observed. Furthermore, the HMBC experiment showed the following correlations: H-2/C-4, H-7/C-6, H-8/C-1, H-8′/C-1′ and C-9, H-7′/C-2′, H-2′/C-4′, H-3-OCH_3_/C-3, H-3′-OCH_3_/C-3′, and H-5′-OCH_3_/C-5′. Except for the additional methoxy group at H-5′ of **5**, its NMR signals were very similar to those of **3**, which was previously reported in a *cis-*conformation. When the two olefinic protons of **3** at δ_H_ 6.59 (1H, d, *J* = 12.4 Hz, H-7) and 5.80 (1H, d, *J* = 12.8 Hz, H-8) were compared with those of **5** at δ_H_ 6.57 (1H, d, *J* = 13.2 Hz, H-7) and 5.78 (1H, d, *J* = 13.2 Hz, H-8), the *cis* conformation was evident. Additionally, when comparing **5** with **6**, which is a previously reported feruloyl amide that is a *trans-*isomer of hibiscusamide, the NMR signals for **5** were similar to those of **6** except for two olefinic protons in *trans-*geometry δ_H_ 7.41 (1H, d, *J* = 15.6 Hz, H-7), and 6.39 (1H, d, *J* = 15.6 Hz, H-8). This result indicates that **5** and **6** are *cis* and *trans*-isomers. Accordingly, compound **5** was determined to be *N*-*cis*-hibiscusamide. To the best of the authors’ knowledge, this is the first time a *cis-*isomer has been reported from this plant.

Compound **9** was collected as a yellowish oil and has a molecular formula of C_19_H_21_NO_6_ as deduced from the molecular peak at *m*/*z* 382.1257 [M + Na]^+^ in the HRESI-MS spectrum. The UV spectrum revealed bands at 221, 283 and 314 nm. The OH and NH groups in the molecule were observed at 3320 (*br*) cm^−1^, and an amide carbonyl group was seen at 1651 cm^−1^ in the IR spectrum. The ^1^H and ^13^C-NMR spectra of **9** were similar to those of **3**, except for the presence of a hydroxyl group at H-7′. In the ^13^C-NMR spectrum of **9**, the presence of 19 resonance signals were classified as two methoxy, one methylene, nine methine, and seven quaternary carbons. The ^1^H-NMR spectrum revealed two olefinic protons at δ_H_ 6.59 (1H, d, *J* = 12.6 Hz, H-7) and 5.79 (1H, d, *J* = 12.6 Hz, H-8); ABX aromatic protons from the ferulic moiety at δ_H_ 7.38 (1H, d, *J* = 1.8 Hz, H-2), 6.91 (1H, dd, *J* = 8.4 Hz, 1.8 Hz, H-6), and 6.72 (1H, d, *J* = 8.4 Hz, H-5); ABX aromatic protons from the normetanephrine moiety at δ_H_ 6.93 (1H, d, *J* = 1.8 Hz, H-2′), 6.76 (1H, dd, *J* = 8.4 Hz, 1.8 Hz, H-6′), and 6.69 (1H, d, *J* = 8.4 Hz, H-5′); methylene protons at 3.39 (1H, dd, *J* = 13.8 Hz, 5.4 Hz, H-8′ α) and δ_H_ 3.46 (1H, dd, *J* = 13.2 Hz, 7.8 Hz, H-8′ β); a methine proton at 4.66 (1H, dd, *J* = 7.8 Hz, 4.8 Hz, H-7′); and two methoxy groups at δ_H_ 3.79 (3H, s, H-3-OCH_3_) and 3.80 (3H, s, H-3′-OCH_3_). The ^1^H and ^13^C resonances of **9** were assigned with a combination of ^1^H-^1^H COSY, HMQC and HMBC experiments (Figure 1B). The *J* values of the olefinic hydrogens of **3** were reported as 12.4 Hz (H-7) and 12.8 Hz (H-8), respectively. The configuration of olefinic hydrogens was confirmed as the *cis-*conformation. The *J* values of H-7 and H-8 of **9** were calculated to be 12.6 Hz and 12.6 Hz compared to those of **3**, respectively. Thus, compound **9** was identified as the *cis-*isomer of *N*-feruloylnormetanephrine. The configuration of C-7′ was determined based on a comparison of optical rotations and *J*-coupling constants with reported data for **8** [24]. The optical rotation values of **8** and **9** were measured as ${\left[\alpha \right]}_{D}^{20}$: −8.2 (c 0.10 CH_3_OH) for **8** and ${\left[\alpha \right]}_{D}^{20}$: −7.8 (c 0.10 CH_3_OH) for **9**. The *J*-coupling constants of H-7′ of **8** and **9** were calculated as *J* = 7.8 Hz, 4.8 Hz for **8** and *J* = 7.8 Hz, 4.8 Hz for **9**. The reported data for **8** [24] included similar optical rotation values, ${\left[\alpha \right]}_{D}^{20}$: −3.0 (c 0.12 CH_3_OH), and *J*-coupling constants of C-7′, *J* = 7.8 Hz, 4.9 Hz, suggesting that the relative configuration at C-7′ was estimated as an *S*-configuration. Thus, compound **9** was determined to be (7′*S*)-*N*-*cis*-feruloylnormetanephrine and is the first reported extraction of this compound from this plant.

### 2.2. Conversion Study of Compounds ***1***–***10***

**Figure 2:**
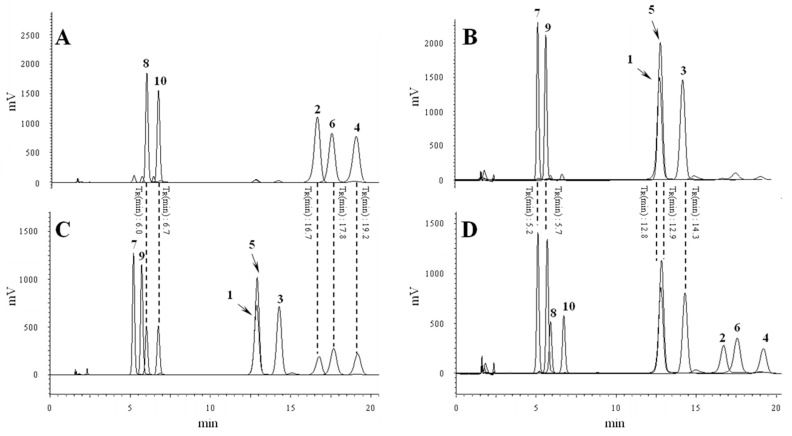
Overlaid HPLC-UV chromatograms obtained at 210 nm of (**A**) isolated *trans*-compounds with purities of ≥95%; (**B**) isolated *cis*-compounds with purities of ≥95%; (**C**) fully converted mixtures from *trans*-compounds after 96 h of UV exposure and (**D**) fully converted mixtures from *cis*-compounds after 96 h of UV (254 nm) exposure.

In the NMR spectra analysis of the *trans*-feruloyl amide, we identified minor peaks that implied the simultaneous presence of small amounts of the *cis*-isomers. These data and a previous phytochemical study on *P. oleracea* led us to consider that the isolated feruloyl amide isomer could be isomerized upon UV light exposure [18,25]. Conversion studies of other compounds with geometric isomers, such as resveratrol [26] and retinal [27], under light exposure have been performed, but the structural isomerization of feruloyl amide from *P. oleracea* has not been evaluated previously. Therefore, we selected the compounds isolated as *cis* and *trans*-isomers and performed conversions under UV light. The isolated *cis* and *trans*-compounds were verified with purities of ≥95% by HPLC. The verified pure compounds were dissolved in MeOH to a concentration of 1 mg/mL and were exposed to UV light at λ = 254 nm for 1, 2, 4, 8, 24, 48 or 96 h. The conversion rates, according to the UV light exposure time, were analyzed by HPLC (Figure 2A,B).

**Figure 3:**
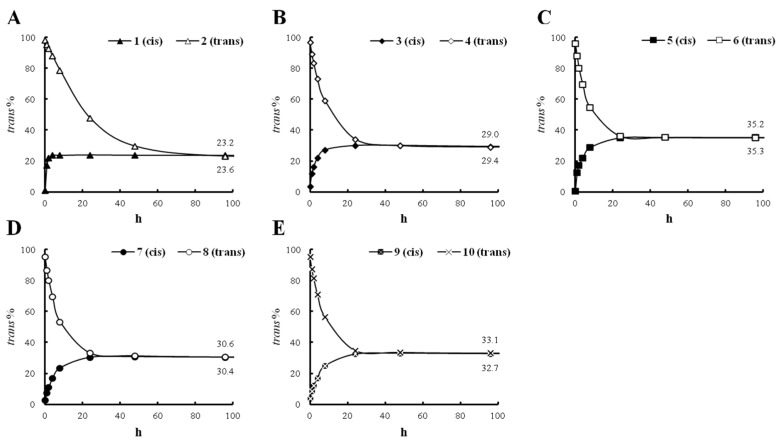
Conversion rates and stabilized ratios of compounds **1** and **2** (**A**); compound **3** and **4** (**B**); compound **5** and **6** (**C**); compound **7** and **8** (**D**); compound **9** and **10** (**E**).

Compound **2**, which was isolated as a *trans-*isomer, has a methoxy group at the tyramine moiety (Figure 1A). The conversion rates of **2**, which were calculated based on the peak area% of the *trans*-isomer in the HPLC chromatograms, showed dramatic changes from 0 h to 24 h: 98.3% at 0 h, 92.6% at 2 h, 78.6% at 8 h, and 47.7% at 24 h (Figure 3A). The data after 24 h (29.6% at 48 h and 23.2% at 96 h) indicated that the conversion rates of the *trans*-isomer rapidly decreased after 48 h and stabilized at 96 h (Figure 3A).

**Figure 4:**
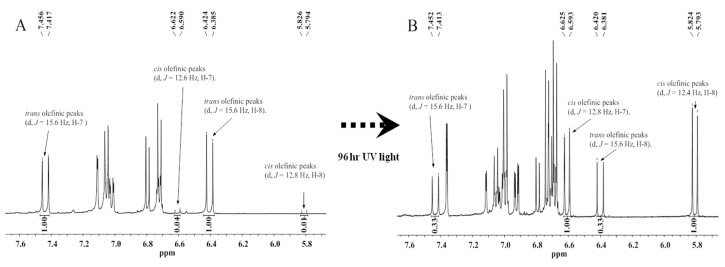
^1^H-NMR (600 MHz) spectra of compound **2**, which was isolated with a purity of ≥95% (**A**); and fully converted compound **2** after 96 h of UV (245 nm) exposure (**B**).

Additionally, to confirm the conversion of the *cis* and *trans*-structures of **2** under UV light, the treatment samples at 0 h and 96 h exposure times were analyzed by ^1^H-NMR (Figure 4A,B). The ratio of the integration values of the two olefinic proton peaks implied that the *cis* (δ_H_ 6.61 (d, *J* = 12.6 Hz, H-7), 5.81 (d, *J* = 12.8 Hz, H-8)) and *trans* (δ_H_ 7.44 (d, *J* = 15.6 Hz, H-7), 6.40 (d, *J* = 15.6 Hz, H-8)) isomers in the ^1^H-NMR spectrum of compound **2** were converted in a *trans*/*cis* proportion = 0.33/1. Under the same conditions, compound **4** (Figure 1A), which has two methoxy groups at the tyramine moiety, showed a more rapid *trans-*conversion rate than **2**: 96.8% at 0 h, 83.4% at 2 h, 58.9% at 8 h, and 34.1% at 24 h (Figure 3B). From 48 h to 96 h, the conversion rate declined. At 96 h, the mixture had equilibrated at a ratio of 29.0% *trans*- and 71.0% *cis*-isomer (Table S1 in the Supplementary Materials). Compared with **2**, we confirmed that the stabilized *trans*-conversion ratio was 5.8% higher (Figure 3B). In the case of compound **6** (Figure 1A), which has three methoxy groups at the tyramine moiety, the *trans-*conversion rates showed similar changes to those of **4**: 95.8% at 0 h, 80.1% at 2 h, 54.6% at 8 h, and 36.1% at 24 h (Figure 3C and Table S1). The conversion rates decreased from 48 h to 96 h. By 96 h, the equilibrium compositions were stabilized with 34.2% of *trans*- and 65.8% of the *cis*-isomer. Compound **6** showed 5.2% and 11.0% higher equilibrium compositions than **4** and **2**, respectively (Figure 3C). From these data, we confirmed that the conversion rate and equilibrium ratio of feruloyl amides are dependent on the number of methoxy groups attached to the tyramine moiety.

The data for compound **8** showed a dissimilar trend from that of **2**, which has a methoxy group. In addition to a methoxy group at the tyramine moiety, compound **8** has a hydroxyl group at the 7′ proton (Figure 1A). The *trans-*conversion rate of **8** was 33.1% at 24 h and stabilized at 30.6% at 96 h. Thus, the conversion rate of **8** was greater and the equilibrium composition was 7.4% higher than that of **2** (Figure 3D). Compound **10** has one more methoxy group at the 5′ position than **8** (Figure 1A). By 24 h, compound **10** showed similar conversion rates as **8**. However, the final equilibrium composition was 2.5% higher than **8** (Figure 3E). Based on these findings, similar to methoxy groups, 7′-hydroxyl group in feruloyl amides might also be a factor in determining conversion and stabilization rates.

The same experiments for the *cis-*isomers, **1** (99.1%), **3** (96.6%), **5** (95.0%), **7** (97.1%) and **9** (96.0%) of *cis*, were conducted as those for the *trans*-isomers. The conversion rates for compound **1** were high, 23.8% at 4 h, and subsequently stabilized (Figure 3A). The other *cis*-compounds, **3** (Figure 3B), **5** (Figure 3C), **7** (Figure 3D) and **9** (Figure 3E), showed conversion rates of 30.0%, 35.0%, 30.3% and 32.3% at 24 h, respectively. Subsequently, after 24 h, these values stabilized. The final equilibrium compositions of the *cis*-compounds were similar to those of the *trans*-compounds: **1** (*cis*):**2** (*trans*) = *trans* 23.6%:23.2%, **3** (*cis*):**4** (*trans*) = *trans* 29.4%:29.0%, **5** (*cis*):**6** (*trans*) = *trans* 35.3%:34.2%, **7** (*cis*):**8** (*trans*) = *trans* 30.4%:30.6% and **9** (*cis*):**10** (*trans*) = *trans* 32.7%:33.1% (Table S1). These data demonstrate that all feruloyl amide *cis* and *trans*-isomers have specific equilibrium ratios which are different from *trans*/*cis* = 23.2%/76.8% to 35.0%/65.0%, depending on the number of methoxy groups and the existence of 7′-hydroxyl group.

In most cases, it is known that *trans*-form geometric isomers are more stable. However, in our conversion study, the *cis*-isomers were found more stable than the *trans*-isomers in the mixtures after equilibration under UV light (254 nm). We found similar cases in photoisomerization studies of isomers that have structures related to feruloyl amides. For example, dicaffeoylquinic acids, except 1,3-dicaffeoylquinic acid, were converted to *cis*-forms as major isomers under UV-light (254 nm) after 30 min [28]. Meanwhile, in the case of resveratrol, *trans*-resveratrol was converted to 90.6% *cis*-resveratrol under 366 nm. However, 50% of *cis*-resveratrol was converted in *trans*-form at certain condition (at pH 1.0 after 22.8 h under the 366 nm) [26]. Therefore, there is a possibility that stabilized feruloyl amides can be converted in to another equilibrium composition under certain conditions. Thus, we consider more studies necessary to investigate the behavior of feruloyl amides and their conversion at various wavelength, pH values and temperature.

The isolated isomers did not exhibit any observable changes after the long-term, three-month stability tests in the dark at room temperature. Further stability experiments using different storage conditions were considered.

### 2.3. Activity of Isolated Compounds

**Figure 5:**
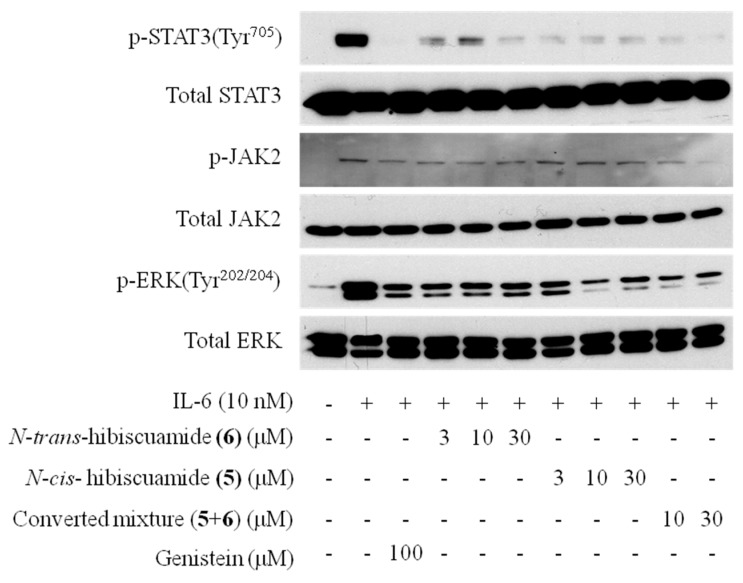
Effects of compounds **5** and **6** and their converted mixture (**5** + **6**) on the STAT3, JAK2 and ERK Phosphorylation by IL-6.

The inhibitory activity of the ten compounds **1**–**10** against IL-6-induced STAT3 activation in Hep3B cells was evaluated using oleanolic acid acetate [29] as a positive control (IC_50_ value: 0.7 μM). An MTT assay was performed to assess cell cytotoxicity and confirm that the effects at the tested concentrations were not due to cytotoxicity (data not shown). All isolated compounds exhibited inhibitory effects, with IC_50_ values of 19.3 (**1**), 5.6 (**2**), 16.2 (**3**), 6.0 (**4**), 6.7 (**5**), 0.2 (**6**), 19.5 (**7**), 2.6 (**8**), 28.6 (**9**) and 13.0 (**10**) μM. The IL-6/STAT3 inhibitory activities of the *trans*- feruloyl amides were more potent than those of the *cis-*isomers, indicating that the *trans-*double bond at C-7′ enhanced the inhibitory activity (Table 2). The conversion mixtures of compounds (**1**–**10**) also exhibited inhibitory activities, with IC_50_ values of 15.2 (**1** + **2**), 6.5 (**3** + **4**), 5.9 (**5** + **6**), 6.7 (**7** + **8**) and 13.7 (**9** + **10**). IC_50_ values of conversion mixtures were in the range of IC_50_ values of their pure *cis* and *trans*-isomers (Table 2). We expected that the IC_50_ values of all mixtures should be close to those of *cis*-feruloyl amides. However, except for (**1** + **2**) and (**5** + **6**), the IC_50_ values of the rest mixtures were close to those of *trans*-feruloyl amides. Therefore, to verify the activities of converted mixtures more clearly, it seems that more experiments investigating the various *cis*/*trans* ratios are needed. In addition, we investigated whether compounds **5** and **6** and their converted mixture (**5** + **6**), which exhibited the greatest inhibitory activities, had inhibitory effects on the tyrosine phosphorylation of STAT3 induced by IL-6 in Hep3B cells. As shown in Figure 5, treatment of Hep3B cells with compounds **5** and **6** for 20 min at 3, 10 and 30 µM resulted in inhibition of the phosphorylation of p-STAT3. The phosphorylation of p-JAK2, the upstream molecule in the IL-6/STAT3 signaling pathway, was also inhibited, as was phosphorylation of p-ERK, another downstream signaling molecule involved in the IL-6 signaling pathway.

## 3. Materials and Methods

### 3.1. Plant Material

Dried *P. oleracea* L. (15 kg) was purchased at the Kyung-dong Market in Seoul, Korea in May 2013. One of the authors (M.-C. Rho) performed botanical identification, and a voucher specimen (KRIB-KR2013-003) has been deposited at the laboratory of the Natural Product Research Center, Jeonbuk Branch of the Korea Research Institute of Bioscience and Biotechnology.

### 3.2. General Procedure

The structures of the isolated compounds were identified by spectroscopy using ^1^H-NMR, ^13^C-NMR, ^1^H-^1^H COSY, HMBC, HMQC, ESI-MS, HRESI-MS, IR, spectrophotometry and polarimetry. ^1^H, ^13^C and 2D NMR spectra were recorded on a JNM-EX400 (Jeol, Tokyo, Japan) and JNM-ECA600 (Jeol) instruments using TMS as references. MS data were obtained with a Jeol JMS-700 and Bruker- maXis 4G (Bruker-Daltonics, Bremen, Germany) mass spectrometers in positive and negative-ion modes. IR spectra were measured on Nicolet 6700 FT-IR (Thermo Scientific, Waltham, MA, USA) and Spectrum GX (Perkin Elmer, Boston, MA, USA) spectrometers. UV spectra were recorded on a Spectramax M_2^e^_ (Molecular Devices, Sunnyvale, CA, USA) spectrophotometer. Optical rotations were measured on a P-2000 polarimeter (Jasco, Tokyo, Japan). The HPLC analyses were performed using a Shimadzu (Tokyo, Japan) HPLC system equipped with a quaternary pump (LC-20AD), an auto-sampler (SIL-20A), a UV-detector (SPD-20A) and a column oven (CTO-20A). To isolate and purify compounds, a Hitachi (Tokyo, Japan) semi-preparative HPLC system with a UV-detector (L-2400) and a quaternary pump (L-2130) was used.

### 3.3. Isolation and Purification Procedures

*P. oleracea* (12 kg) dry powder was extracted three times with 95% EtOH (3 × 36 L) at room temperature, and the EtOH solution was evaporated under reduced pressure. The EtOH extract (612 g) was suspended in H_2_O and partitioned with ethyl acetate (3 × 4 L). The EtOAc fraction (381 g) was chromatographed on a silica gel column (50 cm × 10 cm, 200–400 mesh) and eluted with a hexane/ethyl acetate gradient (100:0–0:100, *v*/*v*) system to generate twelve fractions (fr.3-1–fr.3-12). Fractions 3-9 (12 g) were suspended in MeOH and partitioned with hexane. The MeOH soluble fraction 3-9-1 (8.6 g) was chromatographed on a C_18_ MPLC to generate fractions fr.3-9-1-1–fr.3-9-1-6) with a gradient solvent system containing H_2_O and MeOH (9:1–0:1). Fraction 3-9-1-2 (2.7 g) was re-chromatographed on a C_18_ MPLC to generate fractions fr.3-9-1-2-1–fr.3-9-1-2-7 with a gradient solvent system containing H_2_O and MeOH (9:1–1:1). Compounds **7** (8.1 mg), **8** (38.7 mg), **9** (4.2 mg) and **10** (28.3 mg) were purified from the fr.3-9-1-2-2 fraction (230 mg) by semi-preparative HPLC (Phenomenex Luna 5 μ C18, 150 mm × 21.20 mm i.d., MeCN: H_2_O = 20:80, flow rate 6 mL/min, UV 210 nm). Compounds **1** (8.0 mg), **2** (17.8 mg), **3** (4.3 mg), **4** (48.1 mg), **5** (3.8 mg), and **6** (26.2 mg) were purified from the fr.3-9-1-2-4 fraction (320 mg) by semi-preparative HPLC (Phenomenex Luna 5 μ C18, 150 mm × 21.20 mm i.d., MeCN:H_2_O = 19:81, flow rate 6 mL/min, UV 210 nm). The purity of each compound was verified as ≥95% by HPLC analysis (Figure 2A,B).

*N-cis-Feruloyltyramine* (**1**): Dark yellowish oil. ESI-MS ion peaks at *m*/*z* 314.1 [M + H]^+^ and 312.1 [M − H]. ^1^H-NMR (400 MHz, CD_3_OD): δ_H_ 7.35 (1H, s, H-2), 7.00 (2H, d, *J* = 8.4, H-2′ and H-6′), 6.93 (1H, d, *J* = 8.4, H-6), 6.74 (1H, d, *J* = 7.6, H-5), 6.69 (2H, d, *J* = 8.4, H-3′ and H-5′), 6.61 (1H, d, *J* = 12.4, H-7), 5.81 (1H, d, *J* = 12.4, H-8), 3.83 (3H, s, 3-OCH_3_), 3.40 (2H, t, *J* = 8.0, H-8′), 2.69 (2H, t, *J* = 8.0, H-7′). ^13^C-NMR (100 MHz, none of the instruments given have this field strength CD_3_OD): δ_C_ 157.2 (C-4′), 148.8 (C-4), 148.8 (C-3), 138.5 (C-7), 131.4 (C-1), 130.9 (C-2′ and C-6′), 128.8 (C-1′), 125.0 (C-6), 122.0 (C-8), 116.5 (C-3′ and C-5′), 116.1 (C-5), 114.2 (C-2), 56.6 (C-3-OCH_3_), 42.4 (C-8′), 35.7 (C-7′).

*N-trans*-*Feruloyltyramine* (**2**): Dark brown oil. ESI-MS ion peaks at *m*/*z* 314.1 [M + H]^+^ and 312.1 [M − H]^+^. ^1^H-NMR (400 MHz, CD_3_OD): δ_H_ 7.45 (1H, d, *J* = 15.6 Hz, H-7), 7.10 (1H, d, *J* = 1.6 Hz, H-2), 7.05 (2H, d, *J* = 8.4 Hz, H-2′ and H-6′), 7.01 (1H, dd, *J* = 8.4 Hz, 2.0 Hz, H-6), 6.80 (1H, d, *J* = 8.0 Hz, H-5), 6.72 (2H, d, *J* = 8.6 Hz, H-3′ and H-5′), 6.42 (1H, d, *J* = 15.6 Hz, H-8), 3.86 (3H, s, H-3′-OCH_3_), 3.47 (2H, t, *J* = 7.6 Hz, H-8′), 2.75 (2H, t, *J* = 7.6 Hz, H-7′). ^13^C-NMR (100 MHz, CD_3_OD): δ_C_ 157.1 (C-4′), 150.0 (C-4), 149.4 (C-3), 142.2 (C-7), 131.5 (C-1), 130.9 (C-2′ and C-6′), 128.5 (C-1′), 123.4 (C-6), 119.0 (C-8), 116.6 (C-5), 116.4 (C-3′ and C-5′), 111.7 (C-2), 56.5 (C-3-OCH_3_), 42.5 (C-8′), 35.8 (C-7′).

*N-cis*-*Feruloyl-3′-methoxytyramine* (**3**): Dark green oil. ESI-MS ion peaks at *m*/*z* 344.0 [M + H]^+^ and 342.2 [M − H]^+^. ^1^H-NMR (400 MHz, CD_3_OD): δ_H_ 7.35 (1H, d, *J* = 2.0 Hz, H-2), 6.91 (1H, dd, *J* = 8.0, 2.0 Hz, H-6), 6.75 (1H, d, *J* = 1.6 Hz, H-2′), 6.71 (1H, d, *J* = 8.0 Hz, H-5), 6.68 (1H, d, *J* = 8.0 Hz, H-5′), 6.59 (1H, d, *J* = 12.4 Hz, H-7), 6.59 (1H, dd, *J* = 8.0, 2.0 Hz, H-6′), 5.80 (1H, d, *J* = 12.8 Hz, H-8), 3.81 (3H, s, H-3-OCH_3_), 3.77 (3H, s, H-3′-OCH_3_), 3.41 (2H, t, *J* = 7.2 Hz, H-7′), 2.69 (2H, t, *J* = 7.8 Hz, H-8′). ^13^C-NMR (100 MHz, CD_3_OD): δ_C_ 149.2 (C-3), 148.9 (C-3′), 148.8 (C-4), 146.3 (C-4′), 138.6 (C-7), 132.2 (C-1), 128.8 (C-1′), 125.1 (C-6), 122.4 (C-6′), 121.9 (C-8), 116.4 (C-5), 116.1 (C-5′), 114.2 (C-2′), 113.6 (C-2), 56.5 (C-3-OCH_3_), 56.5 (C-3′-OCH_3_), 42.4 (C-8′), 36.1 (C-7′).

*N-trans*-*Feruloyl-3′-methoxytyramine* (**4**): Dark brown oil. ESI-MS ion peaks at *m*/*z* 344.0 [M + H]^+^ and 342.2 [M − H]^+^. ^1^H-NMR (400 MHz, CD_3_OD): δ_H_ 7.38 (1H, d, *J* = 15.6 Hz, H-7), 7.04 (1H, d, *J* = 1.6 Hz, H-2), 6.95 (1H, dd, *J* = 8.0 Hz, 1.6, H-6), 6.75 (1H, d, *J* = 2.0 Hz, H-2′), 6.73 (1H, d, *J* = 8.0 Hz, H-5), 6.66 (1H, d, *J* = 8.0 Hz, H-5′), 6.60 (1H, dd, *J* = 8.0 Hz, 2.0 Hz, H-6′), 6.36 (1H, d, *J* = 15.6 Hz, H-8), 3.80 (3H, s, H-3-OCH_3_), 3.75 (3H, s, H-3′-OCH_3_), 3.42 (2H, t, *J* = 7.2 Hz, H-8′), 2.70 (1H, t, *J* = 7.2 Hz, H-7′). ^13^C-NMR (100 MHz, CD_3_OD): δ_C_ 150.1 (C-4), 149.5 (C-3), 149.2 (C-3′), 146.2 (C-4′), 142.3 (C-7), 132.3 (C-1′), 128.5 (C-1), 123.4 (C-6), 122.5 (C-6′), 119.0 (C-8), 116.7 (C-5), 116.4 (C-5′), 113.7 (C-2), 111.7 (C-2′), 56.5 (C-3-OCH_3_), 56.5 (C-3′-OCH_3_), 42.6 (C-8′), 36.3 (C-7′).

*N-cis*-*Hibiscusamide* (**5**): Dark brown oil. ${\left[\alpha \right]}_{D}^{20}$: −0.2 (c 0.10 CH_3_OH). HRESI-MS ion peak at *m*/*z* 396.1418 [M + Na]^+^ (calcd for C_20_H_23_NO_6_, 396.1418). UV (CH_3_OH) λ_max_ (log ε): 201 (3.95), 223 (3.56) and 314 nm (3.33). IR (KBr) ν_max_: 3332 (*br*), 1650, 1580, 1517 and 1459 cm^−1^. ^1^H-NMR data in CD_3_OD (600 MHz) and ^13^C-NMR data CD_3_OD (150 MHz) are reported in (Table 1).

*N-trans-Hibiscusamide* (**6**): Dark brown oil. ESI-MS ion peaks at *m*/*z* 374.0 [M + H]^+^ and 372.2 [M − H]^+^. ^1^H-NMR data in CD_3_OD (600 MHz) and ^13^C-NMR data CD_3_OD (150 MHz) are reported in (Table 1).

(*7′S*)-*N-cis*-*Feruloyloctopamine* (**7**): Yellowish oil. ${\left[\alpha \right]}_{D}^{20}$: −7.2 (c 0.10 CH_3_OH). ESI-MS ion peak at *m*/*z* 328.2 [M − H]^+^. ^1^H-NMR (600 MHz, CD_3_OD): δ_H_ 7.37 (1H, d, *J* = 1.8 Hz, H-5), 7.15 (2H*, d*, *J* = 8.4 Hz, H-2 and H-6′), 6.91 (1H, d, *J* = 8.4 Hz, H-6), 6.72 (2H, d, *J* = 8.4 Hz, H-3′ and H-5′), 6.70 (1H, d, *J* = 12.6 Hz, H-7), 6.59 (1H, d, *J* = 7.8 Hz, H-2), 5.79 (1H, d, *J* = 12.6 Hz, H-8), 4.65 (1H, dd, *J* = 7.8, 3.0 Hz, H-7′), 3.81 (3H, s, H-3-OCH_3_), 3.44 (1H, dd, *J* = 13.2 Hz, 4.6 Hz, H-8′β), 3.37 (1H, dd, *J* = 13.8 Hz, 7.8 Hz, H-8′α). ^13^C-NMR (150 MHz, CD_3_OD): δ_C_ 170.5 (C-9), 158.3 (C-4′), 149.6 (C-4), 148.8 (C-3), 139.1 (C-7), 134.5 (C-1), 128.4 (C-2′ and C-6′), 127.9 (C-1′), 125.2 (C-8), 120.9 (C-6), 116.2 (C-3′ and C-5′), 116.1 (C-5), 114.0 (C-2), 73.4 (C-7′), 56.4 (C-3-OCH_3_), 48.1 (C-8′).

(*7′S*)-*N-trans*-*Feruloyloctopamine* (**8**): Yellowish oil. ${\left[\alpha \right]}_{D}^{20}$: −8.2 (c 0.10 CH_3_OH). ESI-MS ion peak at *m*/*z* 328.1 [M − H]^+^. ^1^H-NMR (600 MHz, CD_3_OD): δ_H_ 7.44 (1H, d, *J* = 15.6 Hz, H-7), 7.22 (2H, d, *J* = 8.4 Hz, H-2′ and H-6′), 7.11 (1H, d, *J* = 1.8 Hz, H-5), 7.01 (1H, dd, *J* = 8.4, 1.8 Hz, H-6), 6.79 (1H, d, *J* = 8.4 Hz, H-2), 6.77 (2H, d, *J* = 8.4 Hz, H-3′ and H-5′), 6.46 (1H, d, *J* = 15.6 Hz, H-8), 4.73 (1H, dd, *J* = 7.8 Hz, 4.8 Hz, H-7′), 3.86 (3H, s, H-3-OCH_3_) 3.54 (1H, dd, *J* = 13.2 Hz, 4.6 Hz, H-8′β), 3.45 (1H, dd, *J* = 13.8 Hz, 7.8 Hz, H-8′α). ^13^C-NMR (150 MHz, CD_3_OD): δ_C_ 169.8 (C-9), 158.3 (C-4), 150.1 (C-4′), 149.5 (C-3), 142.5 (C-7), 135.0 (C-1), 128.7 (C-2′ and C-6′), 128.5 (C-1′), 123.5 (C-8), 118.9 (C-6), 116.7 (C-5), 116.3 (C-3′ and C-5′), 111.8 (C-2), 73.6 (C-7′), 56.5 (C-3-OCH_3_), 50.0 (C-8′).

(*7′S*)-*N-cis*-*Feruloylnormetanephrine* (**9**): Yellowish oil. ${\left[\alpha \right]}_{D}^{20}$: −7.8 (c 0.10 CH_3_OH). HRESI-MS ion peak at (*m*/*z* 382.1257 [M + Na]^+^; calcd for C_19_H_21_NO_6_, 382.1261). UV (CH_3_OH) λ_max_ (log ε): 221 (3.24), 283 (3.06) and 314 nm (3.04). IR (KBr) ν_max_: 3320 (*br*), 1651, 1599, 1521 and 1458 cm^−1^. ^1^H-NMR data in CD_3_OD (600 MHz) and ^13^C-NMR data CD_3_OD (150 MHz) are reported in (Table 1).

(*7′S*)-*N*-*trans*-*Feruloylnormetanephrine* (**10**): Yellowish oil. ${\left[\alpha \right]}_{D}^{20}$: −30.4 (c 0.10 CH_3_OH). ESI-MS ion peak at *m*/*z* 358.1 [M − H]^+^. ^1^H-NMR data in CD_3_OD (600 MHz) and ^13^C-NMR data CD_3_OD (150 MHz) are reported in (Table 1).

### 3.4. Stability and Conversion Testing of Cis and trans-Feruloyl Amides

All isolated pure compounds **1**–**10** (verified as ≥95% by HPLC analysis) were dissolved in MeOH to a concentration of 1 mg/mL, and the solutions were stored in the dark at room temperature for stability testing. For conversion tests, 0.1 mL of each solution was used to determine the initial ratio of *cis* and *trans-* by HPLC. Subsequently, the remainder of each solution was then exposed to a UV lamp (30 W, UV output 13.4 W, 253.7 nm) in a dark room for 96 h. A 0.1-mL aliquot of each solution was removed and analyzed by HPLC at 1, 2, 4, 8, 24, 48 and 96 h to determine the ratio of *cis* and *trans-* isomers. HPLC analysis was performed on a Phenomenex Luna 5 μ C18, 150 mm × 4.60 mm i.d column under isocratic solvent conditions (H_2_O/MeCN = 80/20, *v*/*v*, 20 min). The flow rate was 1.0 mL/min, and the injection volume was 10 μL. The detection wavelength was 210 nm, and the column temperature was maintained at 40 °C. The wavelength for detection was set at 210 nm, where all compounds exhibited maximum absorption.

### 3.5. Cell Culture

Human hepatoma Hep3B cells (ATCC No. HB-8064) and myeloma U266 cells (ATCC No. TIB-196^TM^) were obtained from American Type Culture Collection (Rockville, MD, USA) and were maintained in a DMEM medium, supplemented with 10% fetal bovine serum, 50 U/mL penicillin and 50 mg/mL streptomycin, at 37 °C in a 5% CO_2_ incubator. All of the cell culture reagents were purchased from GibcoBRL (Life Technologies, Cergy-Pontoise, France).

### 3.6. Reagents and Chemicals

Recombinant human IL-6 was purchased from R & D systems (Minneapolis, MN, USA). Anti-phospho STAT3 (Tyr^705^) antibody was purchased from Calbiochem (Darmstadt, Germany) and anti-total STAT3, anti-phospho JAK2 (Tyr^1007/1008^), anti-phospho ERK (Tyr^202/204^) were from Cell Signaling Technology (Boston, MA, USA). All reagents including genistein were obtained from Sigma-Aldrich Ltd (St Louis, MO, USA).

### 3.7. Establishment of the Stable Cell Line Expressing pStat3-Luc

Hep3B cells were cotransfected with pStat3-Luc encoding the Stat3 binding site and pcDNA3.1 (+) carrying a hygromycin selection marker (Clontech Laboratories, Palo Alto, CA, USA) using Lipofectamine plus (Invitrogen, Carlsbad, CA, USA). Two days after transfection, cells that stably expressed luciferase were selected with hygromycin (100 μg/mL), and stable clones were expanded. The expression of luciferase in the clones stably expressing pStat3-Luc was confirmed by luciferase assays.

### 3.8. IL-6-Induced STAT3 Activation

Hep3B cells stably expressing pSTAT3-Luc were established as described previously [29]. The Hep3B cells stably expressing pSTAT3-Luc were seeded in 96-well culture plates at 2 × 10^4^ cells/well. After 24 h, cells were subjected to starvation for 12 h, followed by treatment with IL-6 (10 ng/mL) with or without compounds for 12 h. Luciferase activity was measured according to the manufacturer’s protocol (Promega Corp., Madison, WI, USA).

### 3.9. Cell Viability Assay

Hep3B cells were seeded and cultured for 24 h in 96-well culture plates. After 24 h, the culture medium was replaced with serum-free medium supplemented with a sample at the indicated dose. Following culture with the sample for 48 h, MTT (0.5 mg/mL) was added, and after 4 h of incubation at 37 °C, 200 μL of DMSO was added to each well. Cell viability was determined by MTT assay according to the manufacturer’s protocol (Sigma Chemical Co., St Louis, MO, USA).

### 3.10. Western Blot Analysis

Hep3B or U266 cells were stimulated with IL-6 (10 ng/mL) for 20 min after pre-treatment with compound for 1 h. Whole-cell lysates were prepared using a cell lysis buffer (Cell Signaling Technology) and subjected to western blot [29].

## 4. Conclusions

In this work, we evaluated the IL-6/STAT3 inhibitory activity of ten feruloyl amides isolated from *P. oleracea*. Their structures were determined by NMR and MS spectral data. Among the isolated compounds, with *cis*-configured double bonds, compounds **5** and **9** were reported for the first time. In experiments studying the UV-induced *cis*/*trans*-isomerization of the isolated feruloyl amides, we found that all isomeric pairs equilibrated to *trans*/*cis*-ratios of about 22%/77% to 35%/65%. As the number of methoxy groups in the tyramine moiety increased, the equilibrium ratio increased in favor of the *trans-*isomers, and the presence of the 7′-hydroxyl group also increased the *trans*-isomer proportion. Although all isomer compounds exhibited potent inhibitory activity in the STAT3-dependent luciferase assay, the *trans*-feruloyl amides had stronger activity than the *cis-*isomers, and the conversion mixtures maintained in the ranges of IC50 values on their pure *cis* and *trans-*isomers. Among the tested compounds, **5** and **6** exhibited the most potent inhibitory activity against IL-6/STAT3. Also, compound **5**, **6** and their converted mixture (**5** + **6**) exhibited inhibition on the phosphorylation of p-STAT3, p-JAK2 and p-ERK. Based on these results, these isolated feruloyl amides from *P. oleracea* might be useful candidates for lead compounds against inflammatory diseases and their conversion study could be the basis for the design of IL-6 inhibitors.

## Figures and Tables

**Table 1:** ^1^H and ^13^C-NMR data for compounds **5**, **6**, **9** and **10** ^1^.

Positions	5 (*cis*, *Z*)		6 (*trans*, *E*)		9 (*cis*, *Z*)		10 (*trans*, *E*)	
	δ_H_	δ_c_	δ_H_	δ_c_	δ_H_	δ_c_	δ_H_	δ_c_
1	-	128.0	-	127.6	-	126.6	-	128.5
2	7.34 (*d*, 2.0) ^2^	116.2	7.07 (*d*, 2.0)	111.6	7.38 (*d*, 1.8)	112.7	7.09 (*d*, 1.8)	111.8
3	-	149.3	-	149.4	-	147.4	-	149.2
4	-	149.8	-	149.4	-	148.2	-	150.1
5	6.69 (*d*, 8.0)	114.0	6.74 (*d*, 8.0)	117.0	6.72 (*d*, 8.4)	115.9	6.77 (*d*, 7.8)	116.7
6	6.90 (*dd*, 8.0, 2.0)	125.2	6.98 (*dd*, 8.0, 2.0)	123.6	6.91 (*dd*, 8.4, 1.8)	123.8	7.00 (*d*, 7.8)	123.5
7	6.57 (*d*, 13.2)	138.6	7.41 (*d*, 15.6)	142.4	6.59 (*d*, 12.6)	137.7	7.42 (*d*, 15.6)	142.5
8	5.78 (*d*, 13.2)	121.2	6.36 (*d*, 15.6)	118.3	5.79 (*d*, 12.6)	118.6	6.45 (*d*, 15.6)	118.9
9	-	170.6	-	169.5	-	169.2	-	169.8
1′	-	131.3	-	131.4	-	133.9	-	135.7
2′	6.45 (*s*)	107.1	6.50 (*s*)	106.9	6.93 (*d*, 1.8)	109.4	6.97 (*d*, 1.8)	111.1
3′	-	149.3	-	151.5	-	147.6	-	149.6
4′	-	135.2	-	135.2	-	145.9	-	147.4
5′	-	149.0	-	149.9	6.69 (*d*, 8.4)	114.4	6.75 (*d*, 7.8)	116.2
6′	6.45 (*s*)	107.0	6.50 (*s*)	107.2	6.76 (*dd*, 8.4, 1.8)	118.6	6.81 (*dd*, 7.8, 1.8)	120.2
7′	2.70 (*t*, 7.2)	36.6	2.75 (*t*, 7.2)	36.8	4.66 (*dd*, 7.8, 4.8)	72.2	4.71 (*dd*, 7.8, 4.8)	73.8
8′ α	3.42 (*t*, 7.6)	42.4	3.47 (*t*, 7.6)	42.6	3.39 (*dd*, 13.8, 5.4)	46.8	3.43 (*dd*, 13.8, 5.4)	50.0
8′ β	3.42 (*t*, 7.6)	42.4	3.47 (*t*, 7.6)	42.6	3.46 (*dd*, 13.2, 7.8)	46.8	3.52 (*dd*, 13.2, 7.8)	50.0
3-OCH_3_	3.75 (*s*)	56.8	3.80 (*s*)	56.9	3.79 (*s*)	55.0	3.82 (*s*)	55.6
3′-OCH_3_	3.75 (*s*)	56.8	3.80 (*s*)	56.9	3.80 (*s*)	55.0	3.84 (*s*)	55.5
5′-OCH_3_	3.79 (*s*)	56.4	3.85 (*s*)	56.5	-	-	-	-

^1^ ^1^H-NMR data in CD_3_OD (600 MHz), ^13^C-NMR data in CD_3_OD (150 MHz); ^2^ Chemical shifts (δ) in ppm relative to TMS, *J* in Hz.

**Table 2:** IC_50_ values of compounds **1**–**10** for the inhibition of the induction of STAT3 by IL-6.

Compounds	IC_50_ (μM)
*N*-*cis*-feruloyltyramine (**1**)	19.3
*N*-*trans*-feruloyltyramine (**2**)	5.6
Converted mixture (**1** + **2**)	15.2
*N*-*cis*-feruloyl-3′-methoxytyramine (**3**)	16.2
*N*-*trans*-feruloyl-3′-methoxytyramine (**4**)	6.0
Converted mixture (**3** + **4**)	6.5
*N*-*cis*-hibiscusamide (**5**)	6.7
*N*-*trans*-hibiscusamide (**6**)	0.2
Converted mixture (**5** + **6**)	5.9
(7′*S*)-*N*-*cis*-feruloyloctopamine (**7**)	19.5
(7′*S*)-*N*-*trans*-feruloyloctopamine (**8**)	2.6
Converted mixture (**7** + **8**)	6.7
(7′*S*)-*N*-*cis*-feruloylnormetanephrine (**9**)	28.6
(7′*S*)-*N*-*trans*-feruloylnormetanephrine (**10**)	13.0
Converted mixture (**9** + **10**)	13.7
Oleanolic acid acetate ^1^	0.7

^1^ Oleanolic acid acetate was used as the positive control.

## Supplementary Materials

The following are available online at http://www.mdpi.com/1420-3049/21/7/865/s1.
