# Supplementary material for: Study of the UV Light Conversion of Feruloyl Amides from Portulaca oleracea and Their Inhibitory Effect on IL-6-Induced STAT3 Activation
Source: Molecules. 2016 Jun 30;21(7):865. doi: 10.3390/molecules21070865 (PMC6273636; doi:10.3390/molecules21070865)
Supplement: Supplementary file 1 [file molecules-21-00865-s001.pdf]

Supplementary Materials: Conversion Study of Feruloyl Amides from *Portulaca oleracea* by UV Light and Their Inhibitory Effect on IL-6-Induced STAT3 Activation

Joo Tae Hwang, Yesol Kim, Hyun-Jae Jang, Hyun-Mee Oh, Chi-Hwan Lim, Seung Woong Lee and Mun-Chual Rho

	Content	Page
**Figure S1.**	ESI/MS (Positive) spectrum of **1**	S2
**Figure S2.**	ESI/MS (Negative) spectrum of **1**	S2
**Figure S3.**	^{1}H-NMR (400 MHz, CD_3OD) spectrum of **1**	S3
**Figure S4.**	^{13}C-NMR (125 MHz, CD_3OD) spectrum of **1**	S4
**Figure S5.**	ESI/MS (Positive) spectrum of **2**	S5
**Figure S6.**	ESI/MS (Negative) spectrum of **2**	S5
**Figure S7.**	^{1}H-NMR (400 MHz, CD_3OD) spectrum of **2**	S6
**Figure S8.**	^{13}C-NMR (125 MHz, CD_3OD) spectrum of **2**	S7
**Figure S9.**	ESI/MS (Positive) spectrum of **3**	S8
**Figure S10.**	ESI/MS (Negative) spectrum of **3**	S8
**Figure S11.**	^{1}H-NMR (400 MHz, CD_3OD) spectrum of **3**	S9
**Figure S12.**	^{13}C-NMR (125 MHz, CD_3OD) spectrum of **3**	S10
**Figure S13.**	ESI/MS (Positive) spectrum of **4**	S11
**Figure S14.**	ESI/MS (Negative) spectrum of **4**	S11
**Figure S15.**	^{1}H-NMR (400 MHz, CD_3OD) spectrum of **4**	S12
**Figure S16.**	^{13}C-NMR (125 MHz, CD_3OD) spectrum of **4**	S13
**Figure S17.**	HRESI/MS spectrum of **5**	S14
**Figure S18.**	UV spectra of **5**	S14
**Figure S19.**	IR spectra of **5**	S15
**Figure S20.**	^{1}H-NMR (600 MHz, CD_3OD) spectrum of **5**	S16
**Figure S21.**	^{13}C-NMR (150 MHz, CD_3OD) spectrum of **5**	S17
**Figure S22.**	^{1}H-^{1}H COSY spectrum of **5**	S18
**Figure S23.**	HMBC spectrum of **5**	S19
**Figure S24.**	HMQC spectrum of **5**	S20
**Figure S25.**	ESI/MS (Positive) spectrum of **6**	S21
**Figure S26.**	ESI/MS (Negative) spectrum of **6**	S21
**Figure S27.**	^{1}H-NMR (600 MHz, CD_3OD) spectrum of **6**	S22
**Figure S28.**	^{13}C-NMR (150 MHz, CD_3OD) spectrum of **6**	S23
**Figure S29.**	ESI/MS (Positive) spectrum of **7**	S24
**Figure S30.**	ESI/MS (Negative) spectrum of **7**	S24
**Figure S31.**	^{1}H-NMR (600 MHz, CD_3OD) spectrum of **7**	S25
**Figure S32.**	^{13}C-NMR (150 MHz, CD_3OD) spectrum of **7**	S26
**Figure S33.**	ESI/MS (Positive) spectrum of **8**	S27
**Figure S34.**	ESI/MS (Negative) spectrum of **8**	S27
**Figure S35.**	^{1}H-NMR (600 MHz, CD_3OD) spectrum of **8**	S28
**Figure S36.**	^{13}C-NMR (150 MHz, CD_3OD) spectrum of **8**	S29
**Figure S37.**	HRESI/MS spectrum of **9**	S30
**Figure S38.**	UV spectra of **9**	S30
**Figure S39.**	IR spectra of **9**	S31
**Figure S40.**	^{1}H-NMR (600 MHz, CD_3OD) spectrum of **9**	S32
**Figure S41.**	^{13}C-NMR (150 MHz, CD_3OD) spectrum of **9**	S33
**Figure S42.**	^{1}H-^{1}H COSY spectrum of **9**	S34
**Figure S43.**	HMBC spectrum of **9**	S35
**Figure S44.**	HMQC spectrum of **9**	S36
**Figure S45.**	ESI/MS (Positive) spectrum of **10**	S37
**Figure S46.**	ESI/MS (Negative) spectrum of **10**	S37
**Figure S47.**	^{1}H-NMR (600 MHz, CD_3OD) spectrum of **10**	S38
**Figure S48.**	^{13}C-NMR (150 MHz, CD_3OD) spectrum of **10**	S39
**Figure S49.**	HPLC chromatogram of *P. oleracea* (*dry powder*) which refined by Silica-SPE (eluted with a Hexane:EtOAc = 7:3).	S10
**Figure S50.**	Conversion rates and equilibrium ratio of Compound **1** and **2** (**A**); Compound **3** and **4** (**B**); Compound **5** and **6** (**C**); Compound **7** and **8** (**D**); Compound **9** and **10** (**E**).	S41
**Figure S51.**	The HPLC chromatograms of **1** (**A**); **2** (**B**); **3** (**C**); **4** (**D**); **5** (**E**); **6** (**F**); **7** (**G**); **8** (**H**); **9** (**I**) and **10** (**J**).	S42
**Table S1.**	Conversion rates of *cis* and *trans-* feruloyl amides (**1**–**10**)	S43

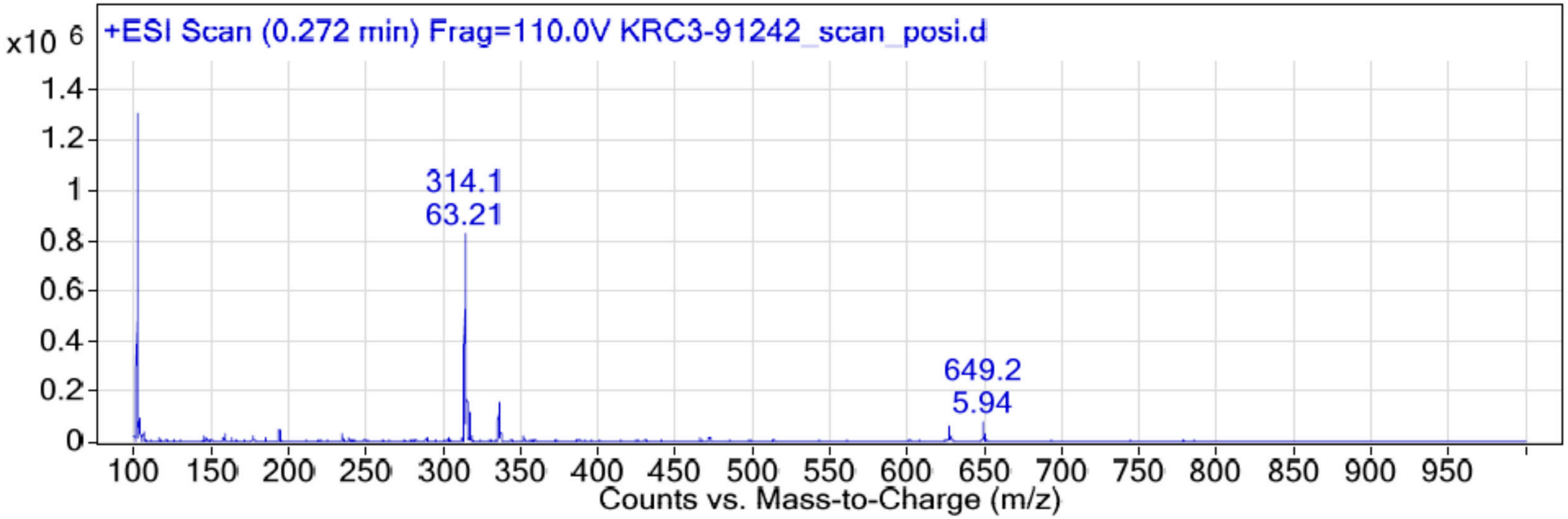

Figure S1. ESI/MS (Positive) spectrum of **1**.

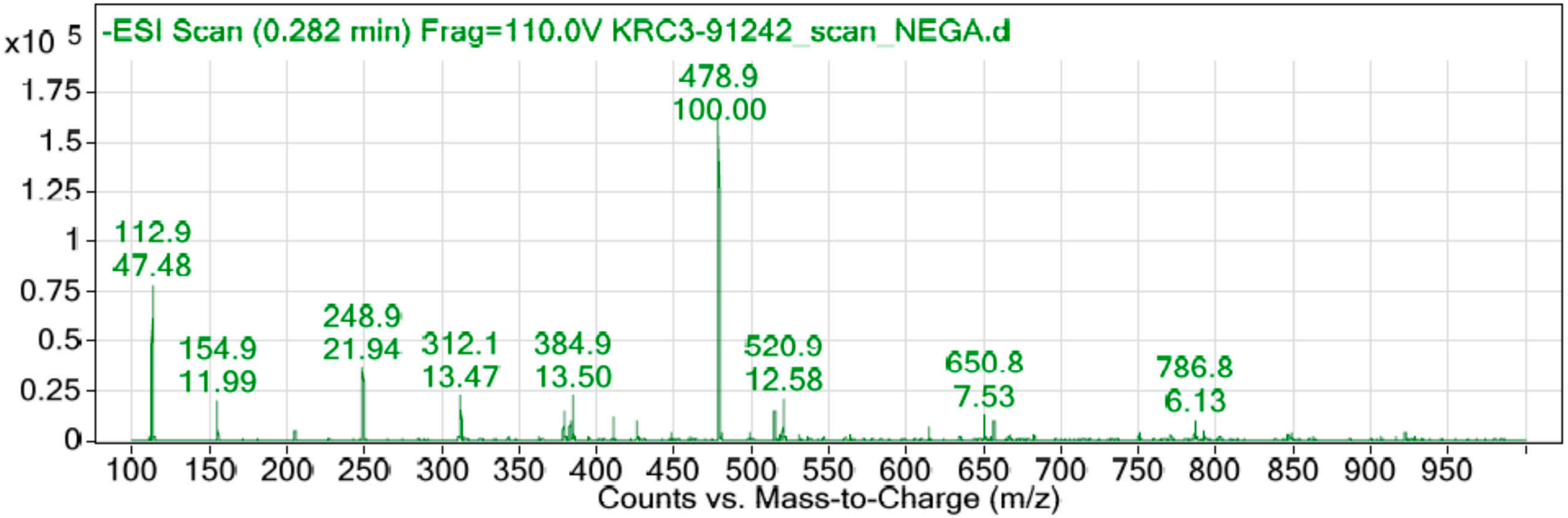

Figure S2. ESI/MS (Negative) spectrum of **1**.

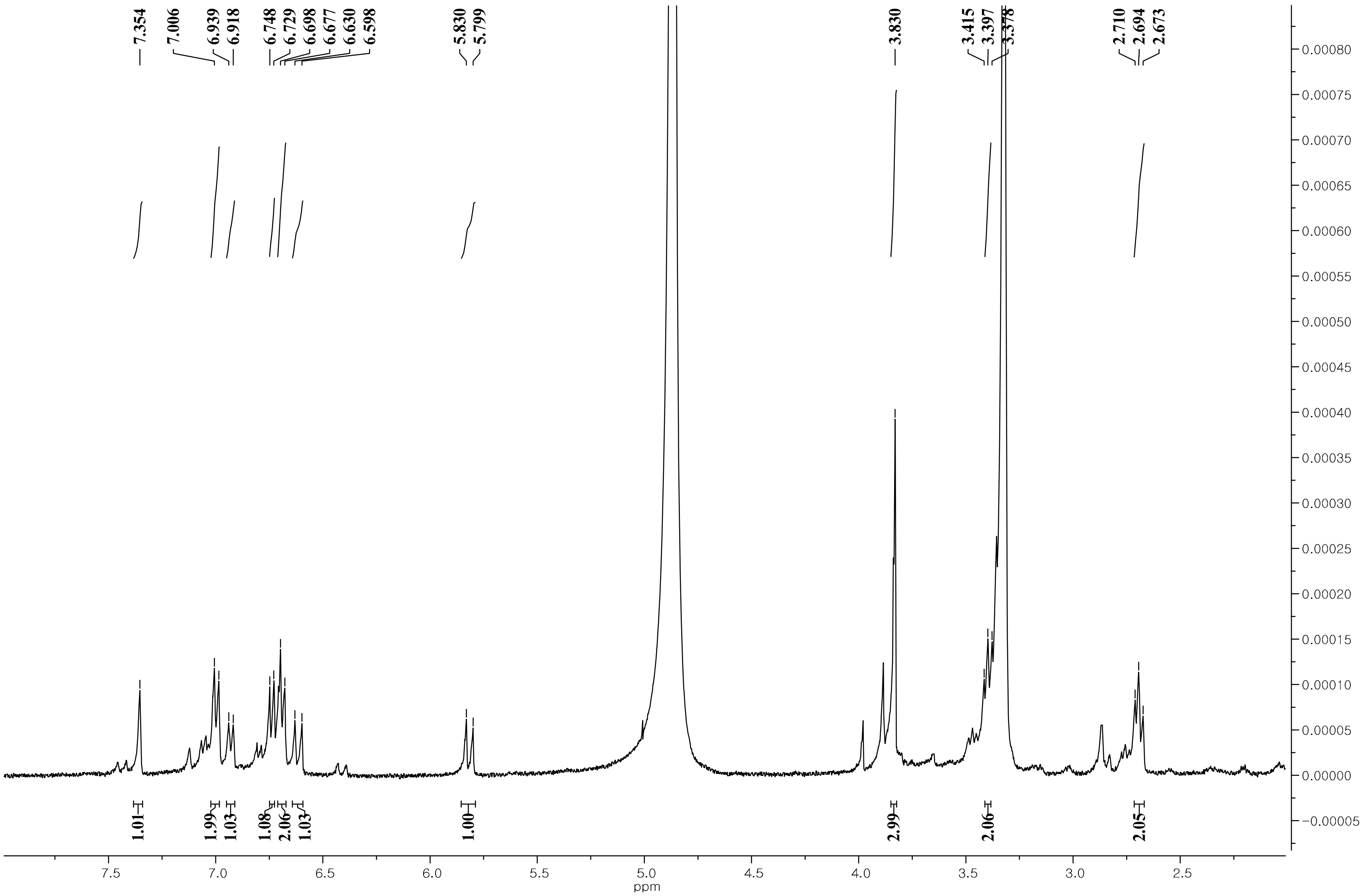

Figure S3. ^{1}H-NMR (400 MHz, CD_3OD) spectrum of **1**.

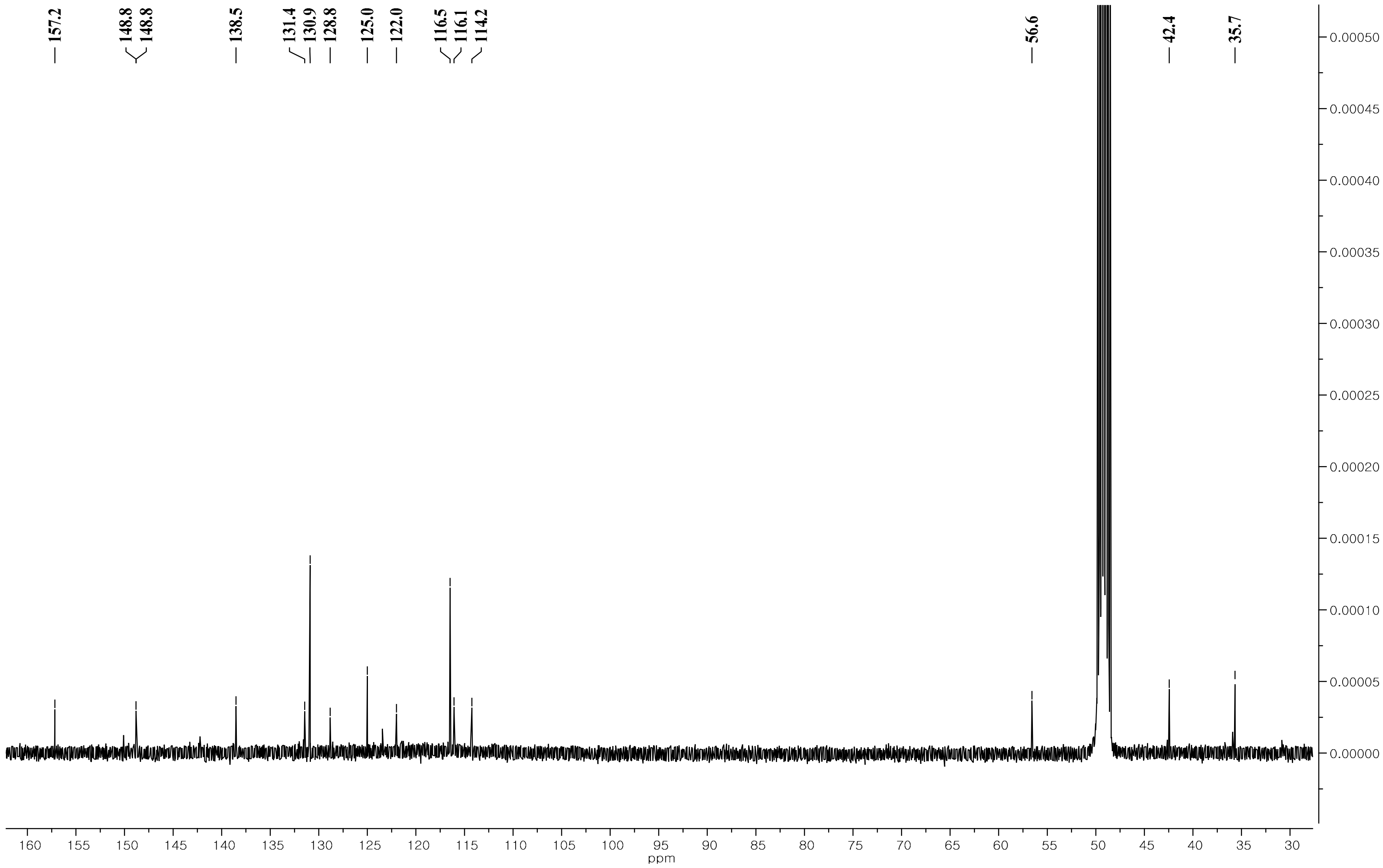

Figure S4. ^{13}C-NMR (125 MHz, CD_3OD) spectrum of **1**.

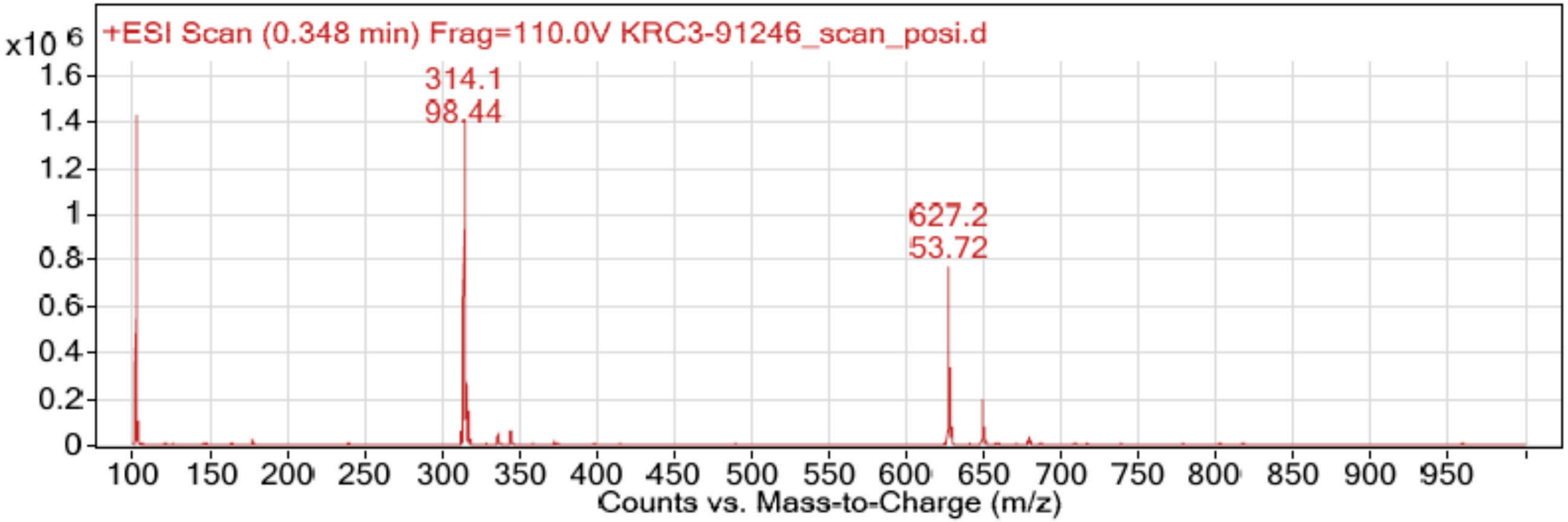

Figure S5. ESI/MS (Positive) spectrum of **2**.

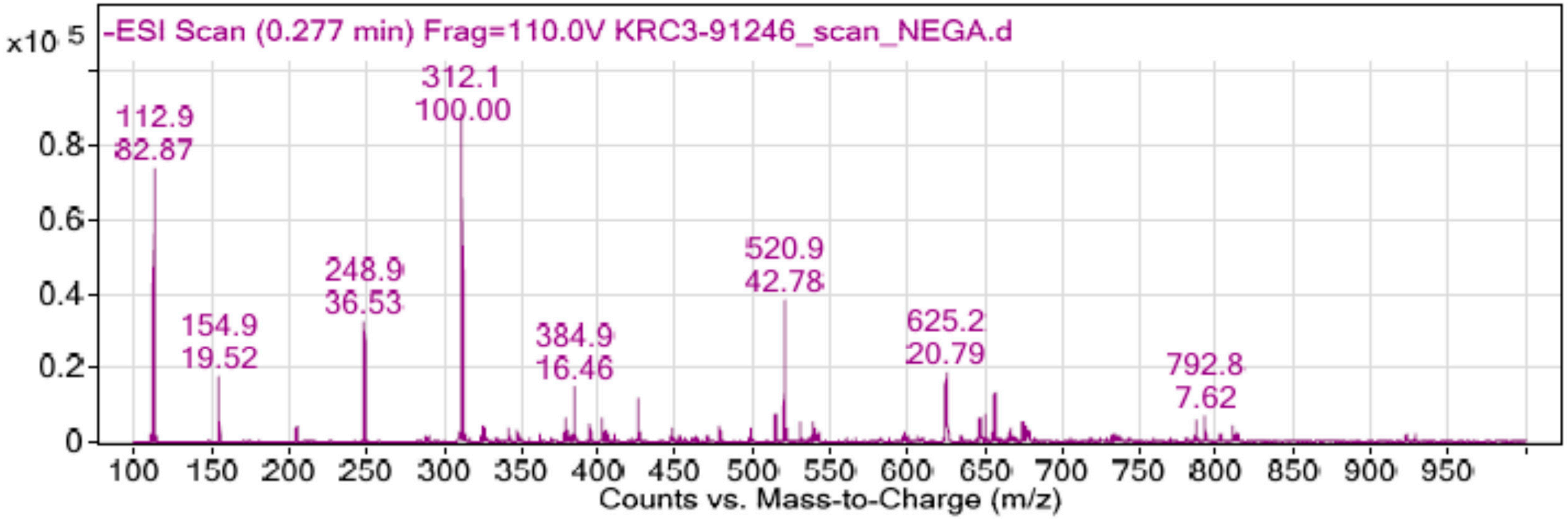

Figure S6. ESI/MS (Negative) spectrum of **2**.

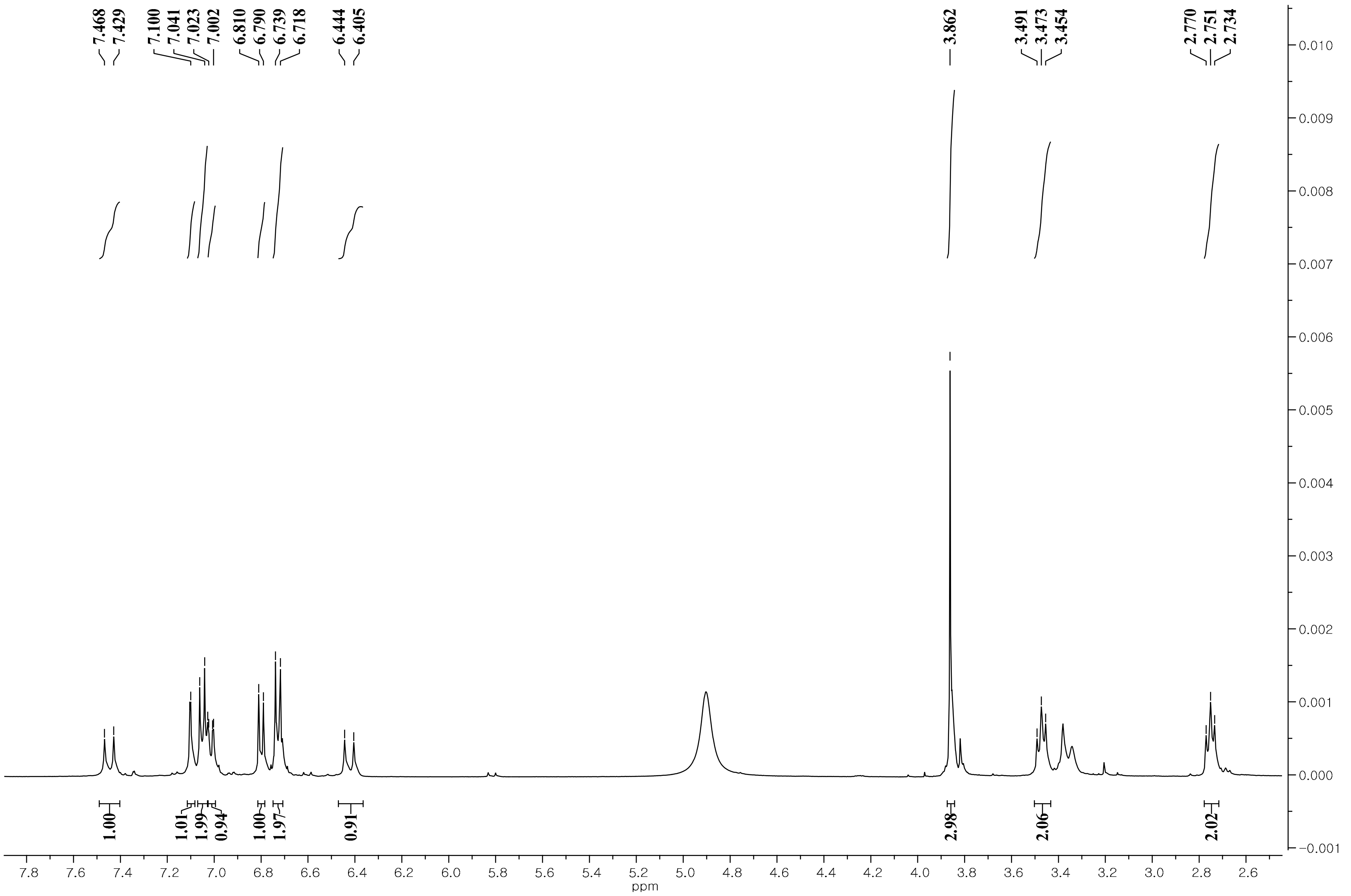

Figure S7. ^{1}H-NMR (400 MHz, CD_3OD) spectrum of **2**.

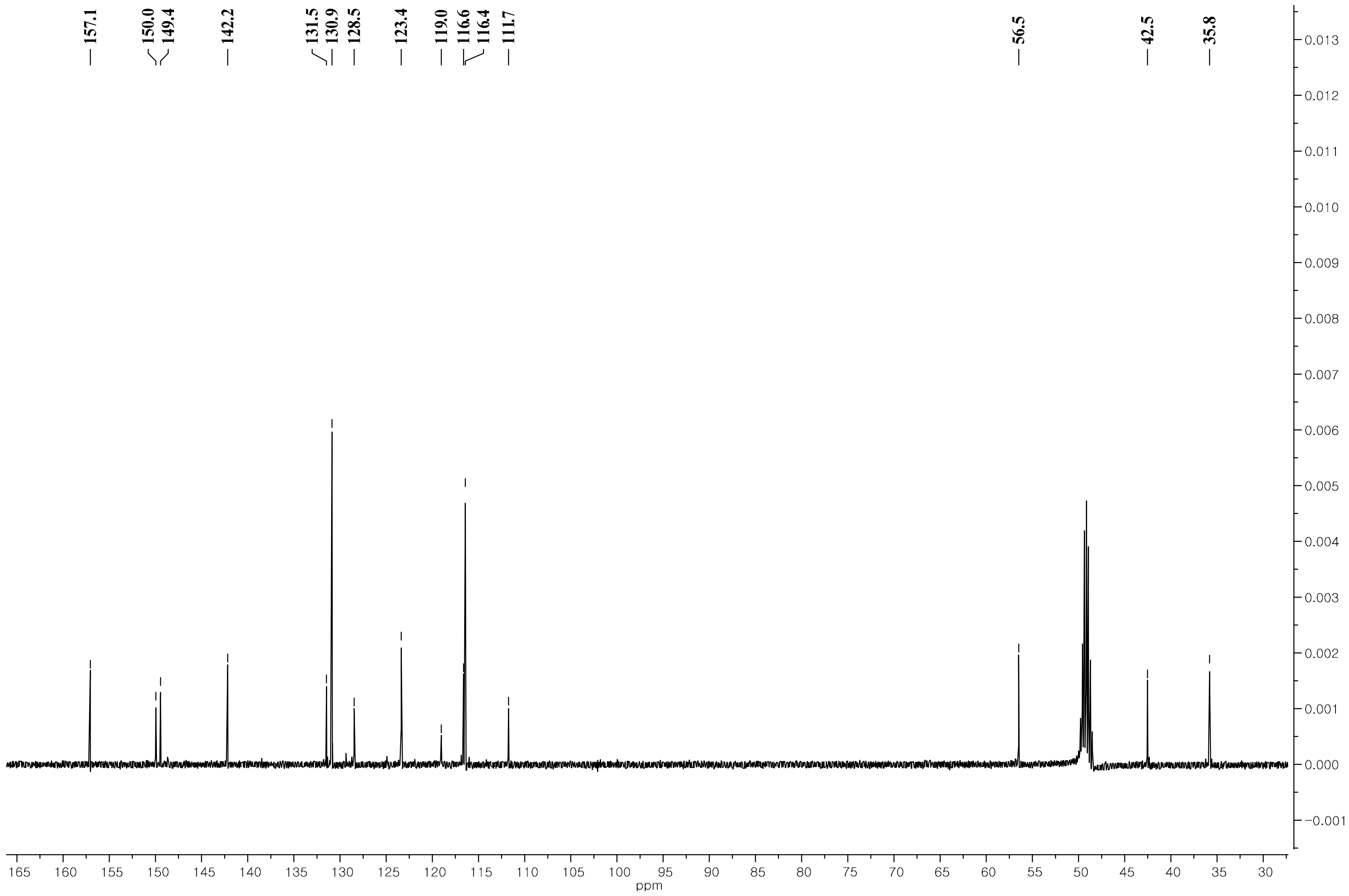

Figure S8. ^{13}C-NMR (125 MHz, CD_3OD) spectrum of **2**.

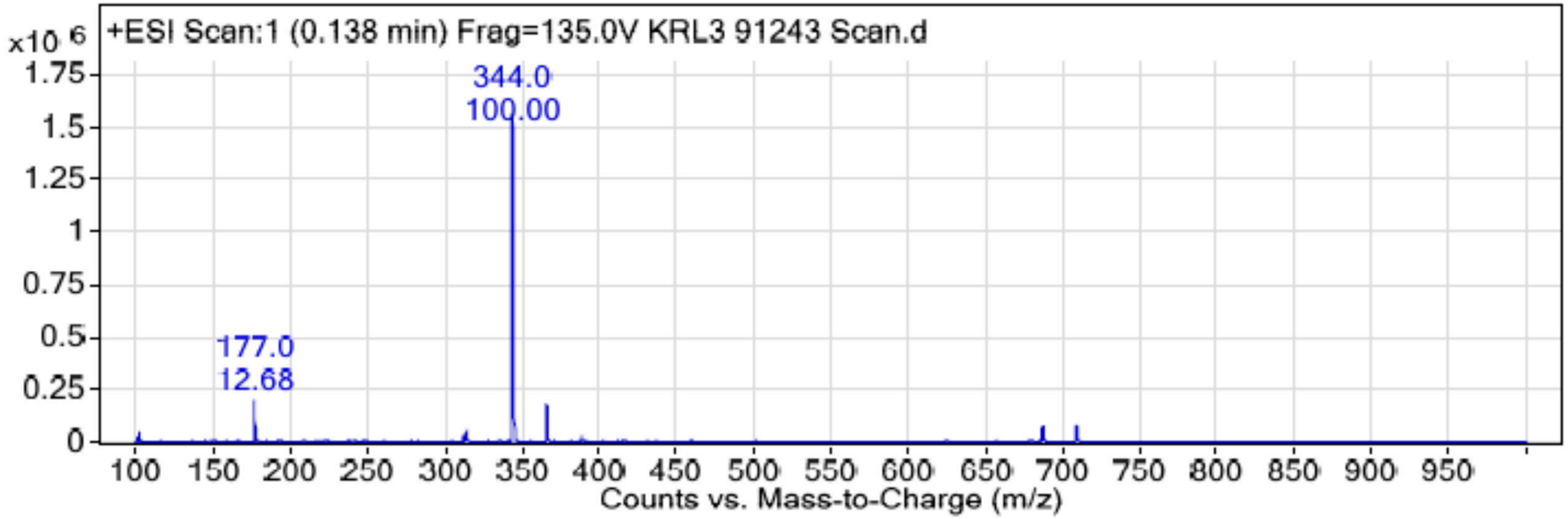

Figure S9. ESI/MS (Positive) spectrum of **3**.

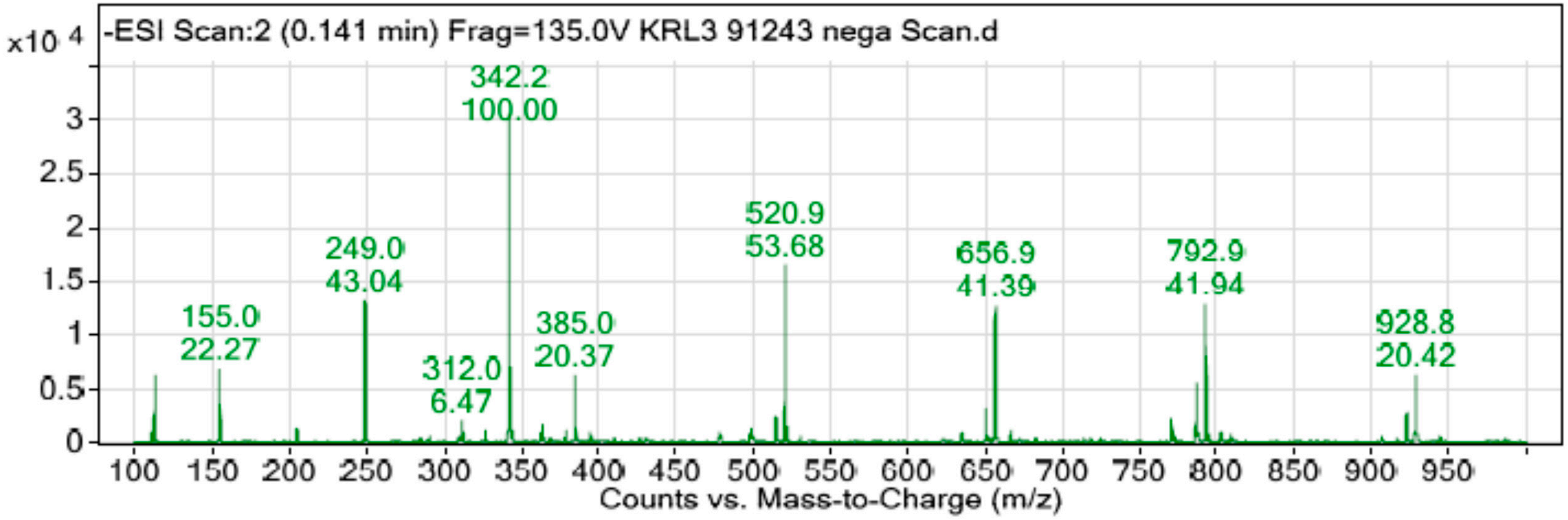

Figure S10. ESI/MS (Negative) spectrum of **3**.

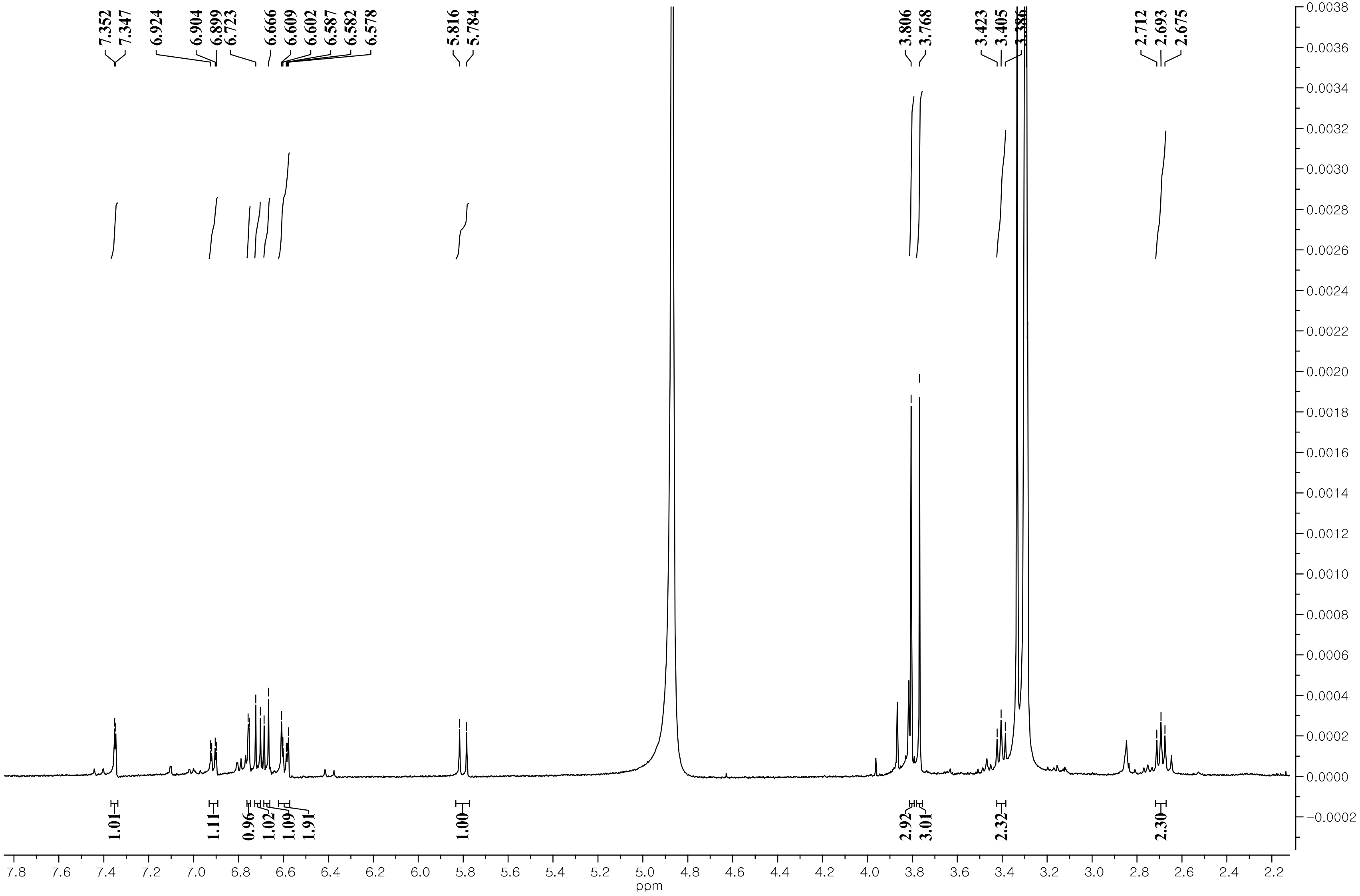

Figure S11. ¹H-NMR (400 MHz, CD_3OD) spectrum of **3**.

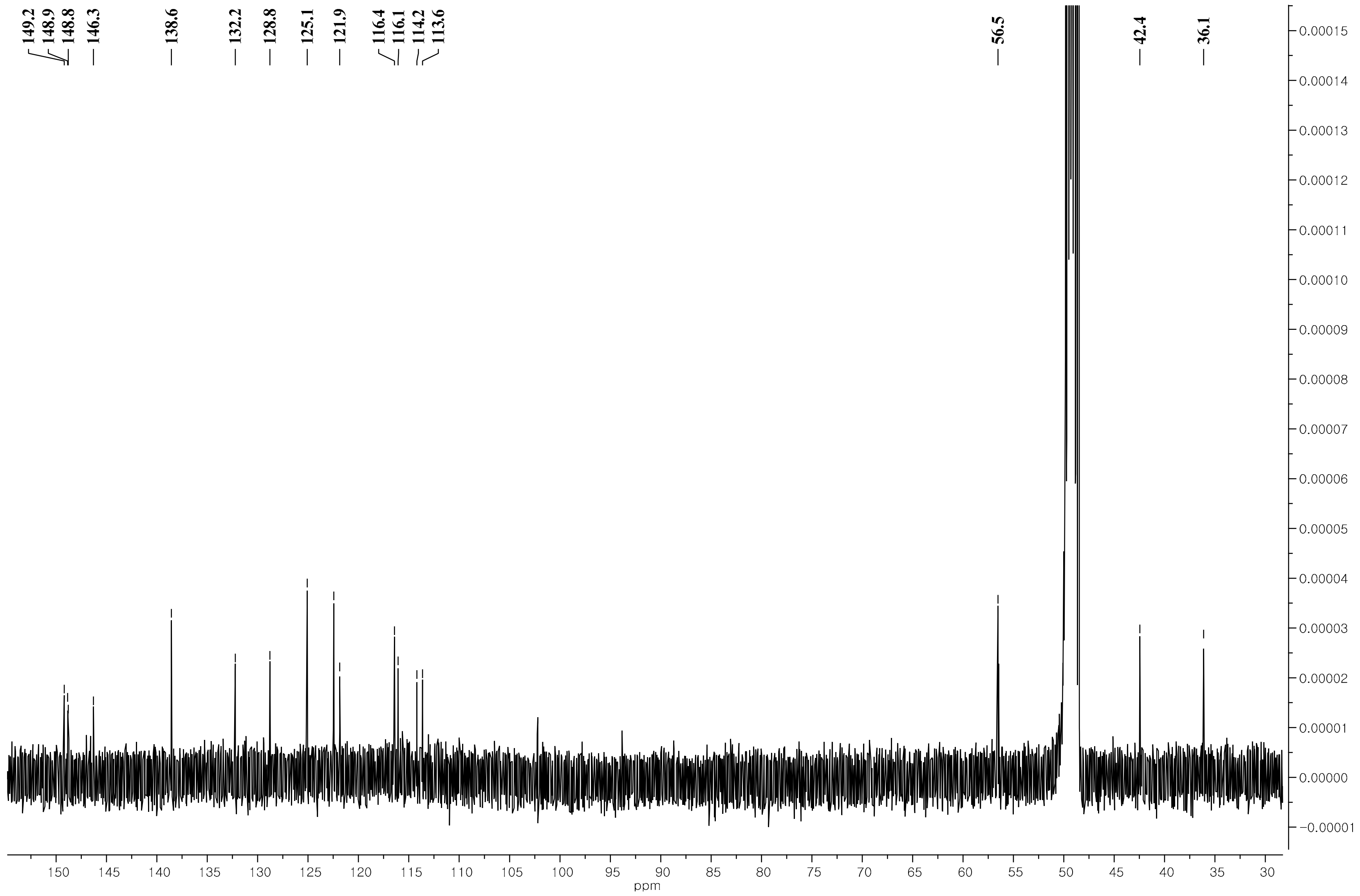

Figure S12. ^{13}C-NMR (125 MHz, CD_3OD) spectrum of **3**.

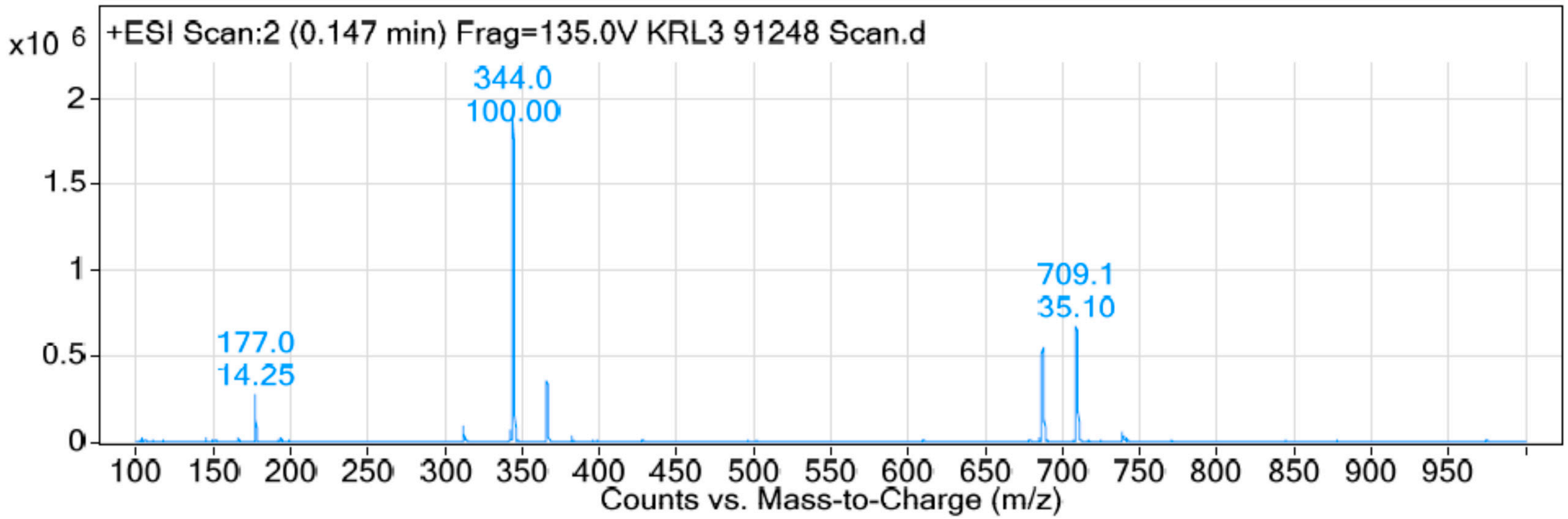

Figure S13. ESI/MS (Positive) spectrum of **4**.

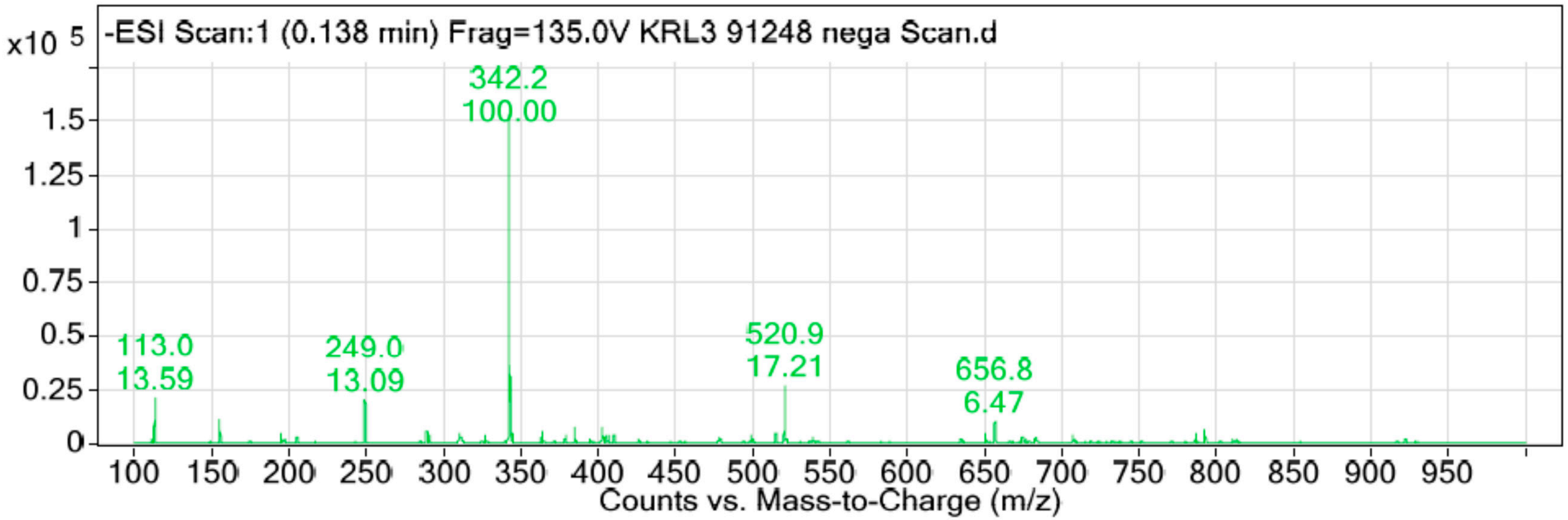

Figure S14. ESI/MS (Negative) spectrum of **4**.

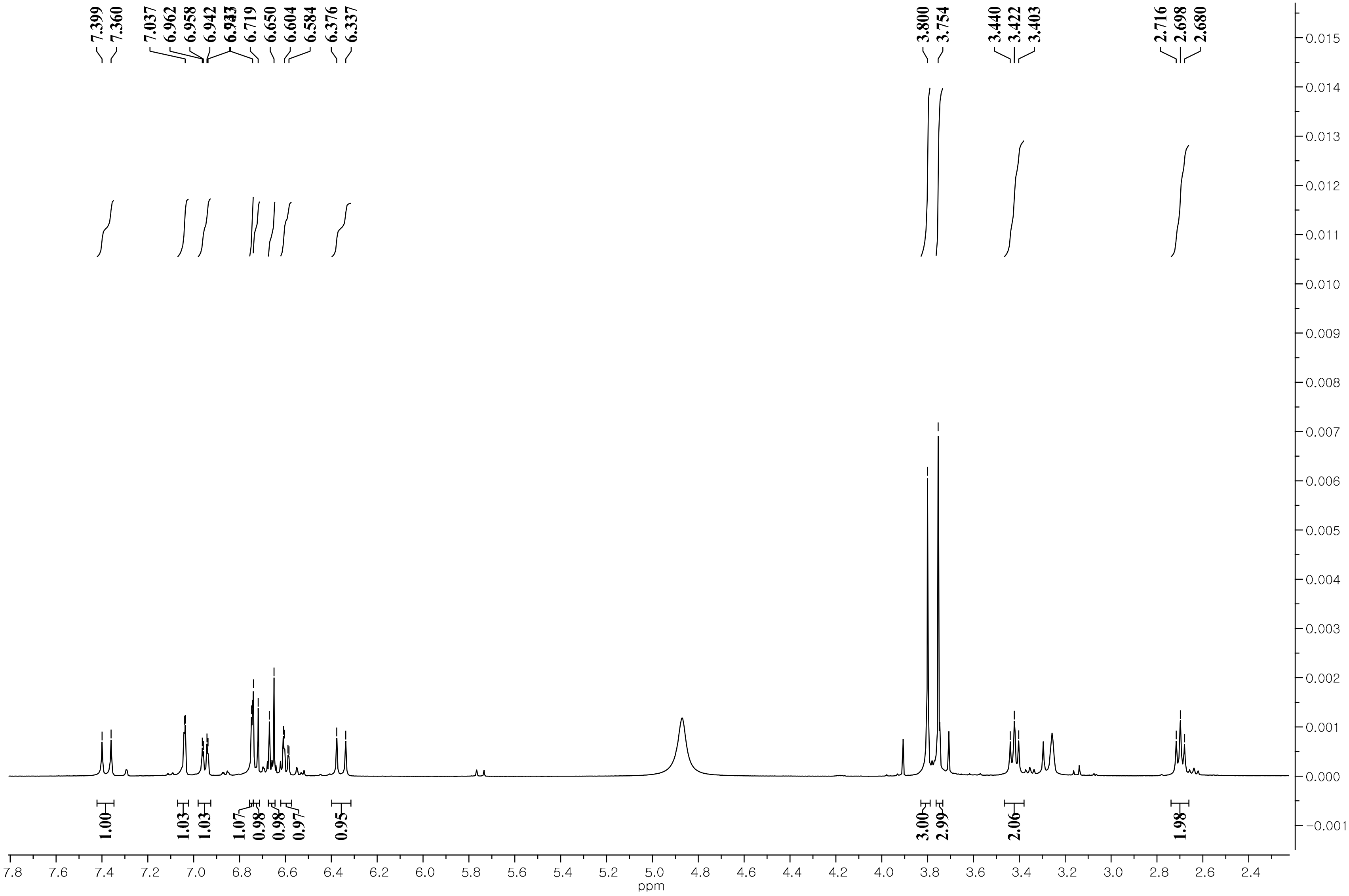

Figure S15. ^{1}H-NMR (400 MHz, CD3OD) spectrum of **4**.

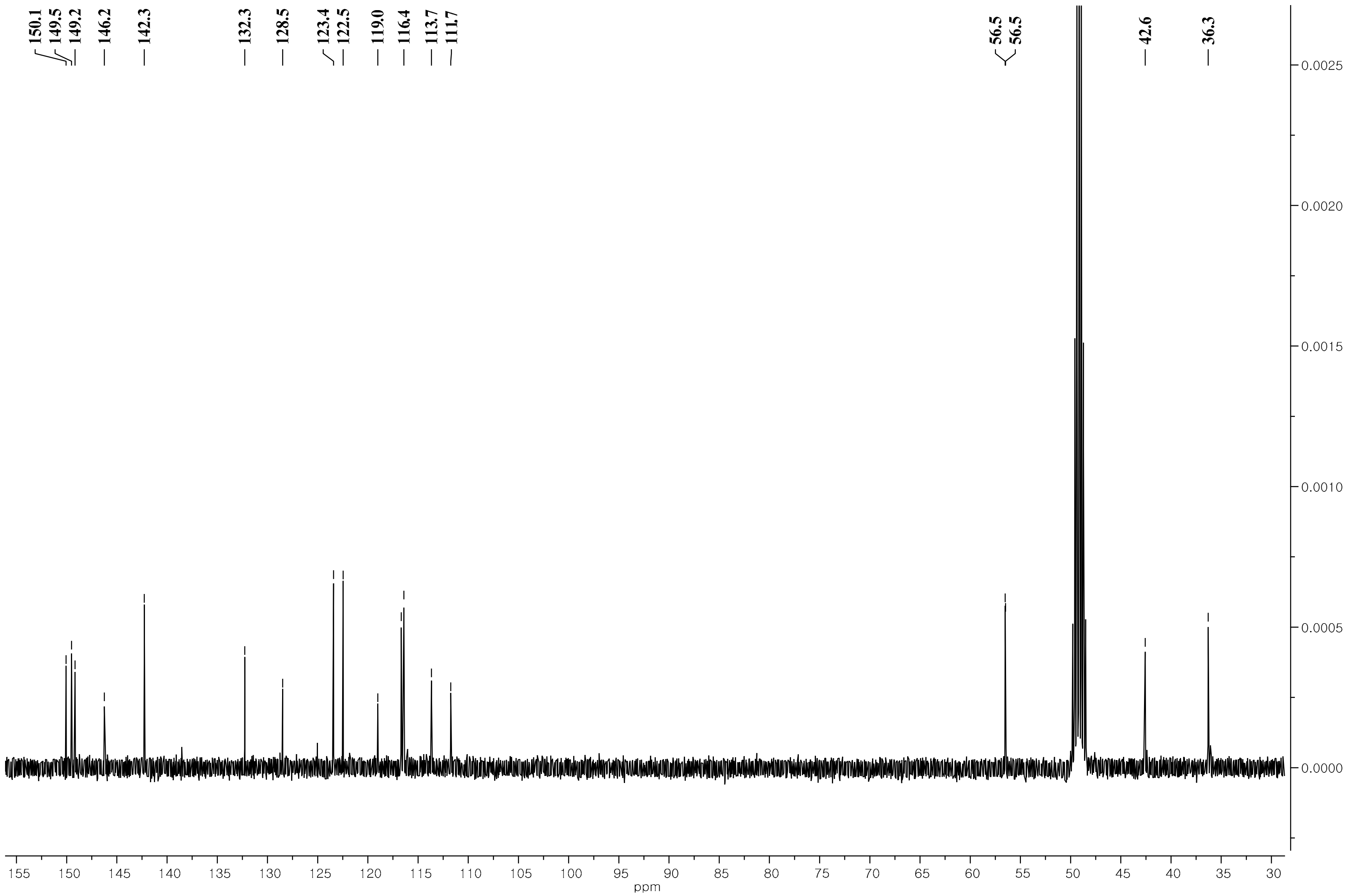

Figure S16. ^{13}C-NMR (125 MHz, CD_3OD) spectrum of **4**.

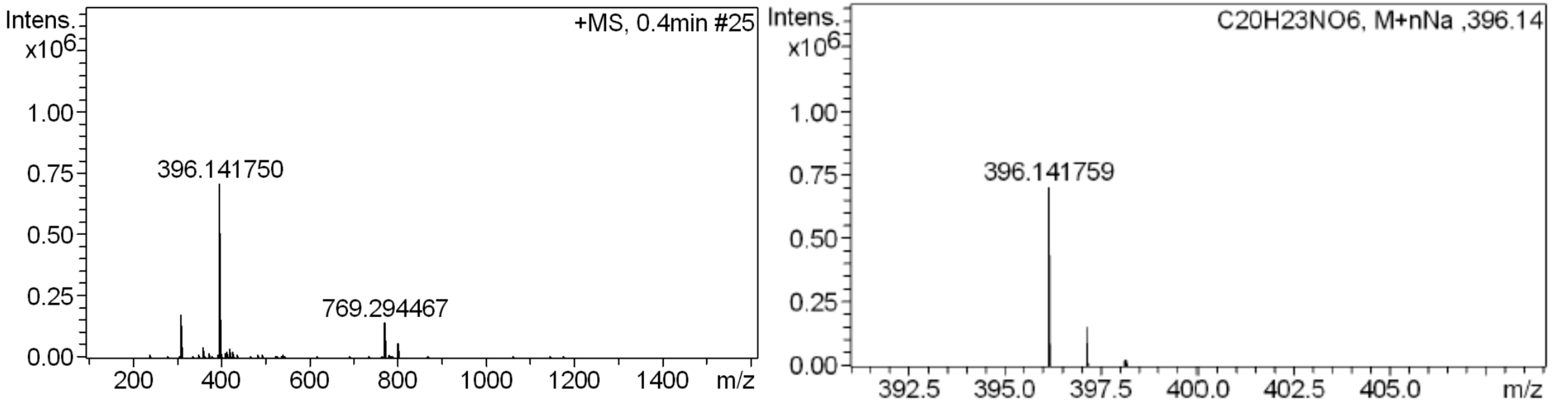

Figure S17. HRESI/MS spectrum of **5**.

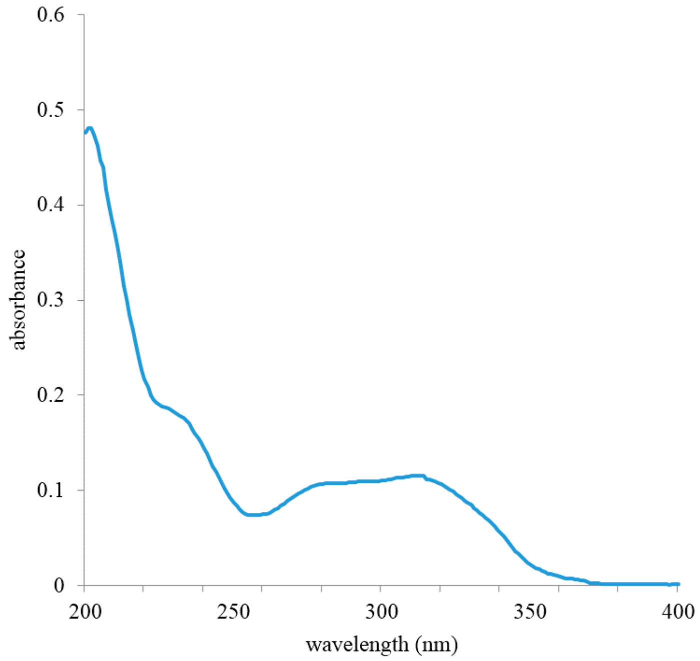

Figure S18. UV spectra of **5**.

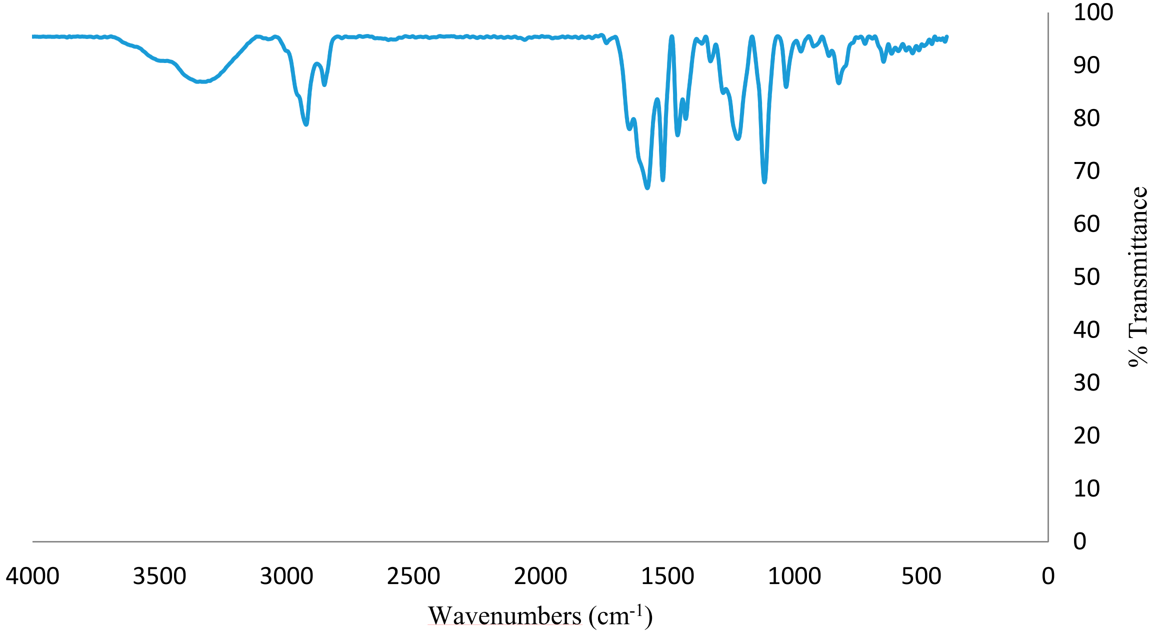

Figure S19. IR spectra of **5**.

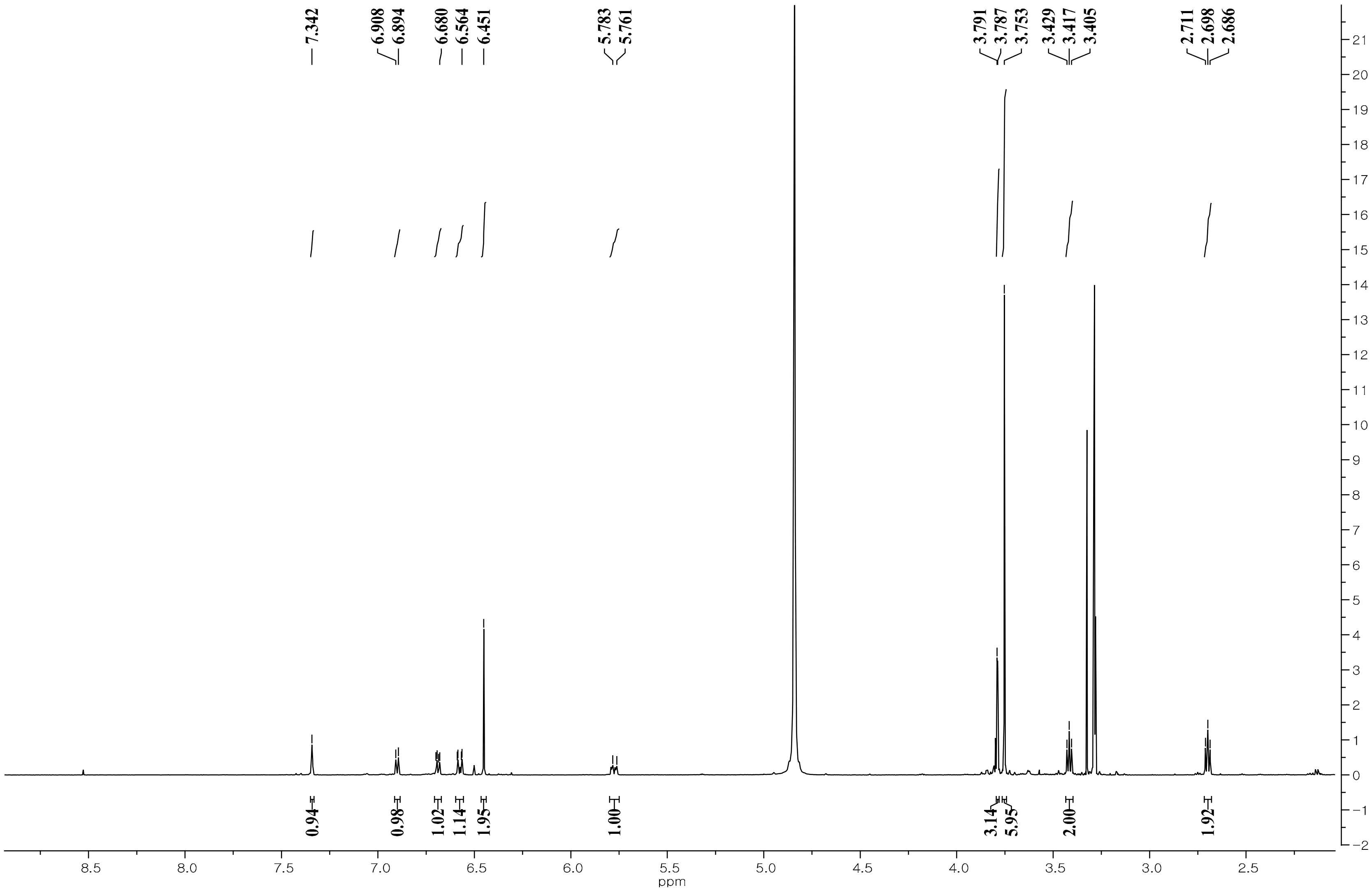

Figure S20. ^{1}H-NMR (600 MHz, CD_3OD) spectrum of **5**.

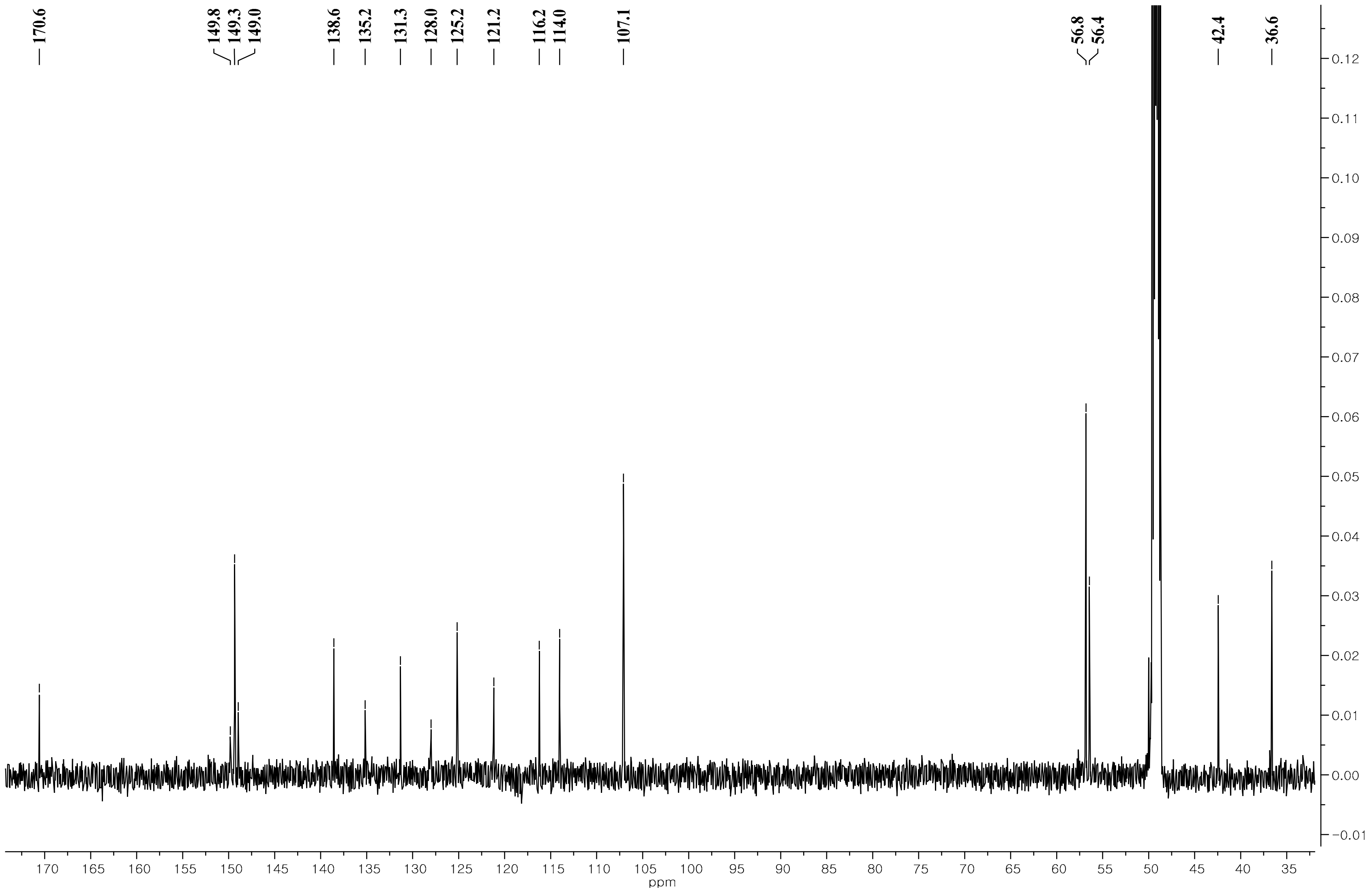

Figure S21. ^{13}C-NMR (150 MHz, CD_3OD) spectrum of **5**.

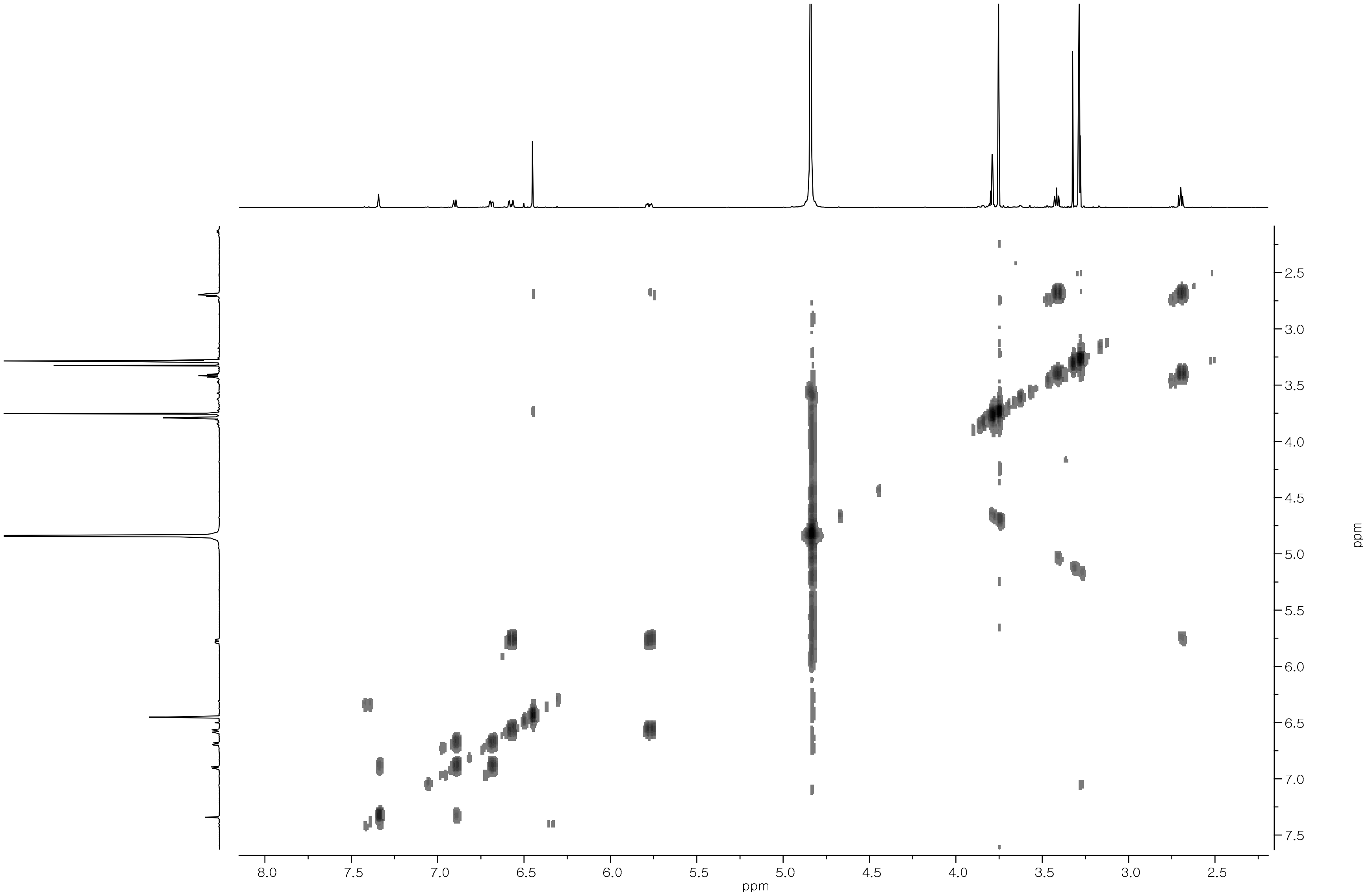

Figure S22. ^{1}H-^{1}H COSY spectrum of **5**.

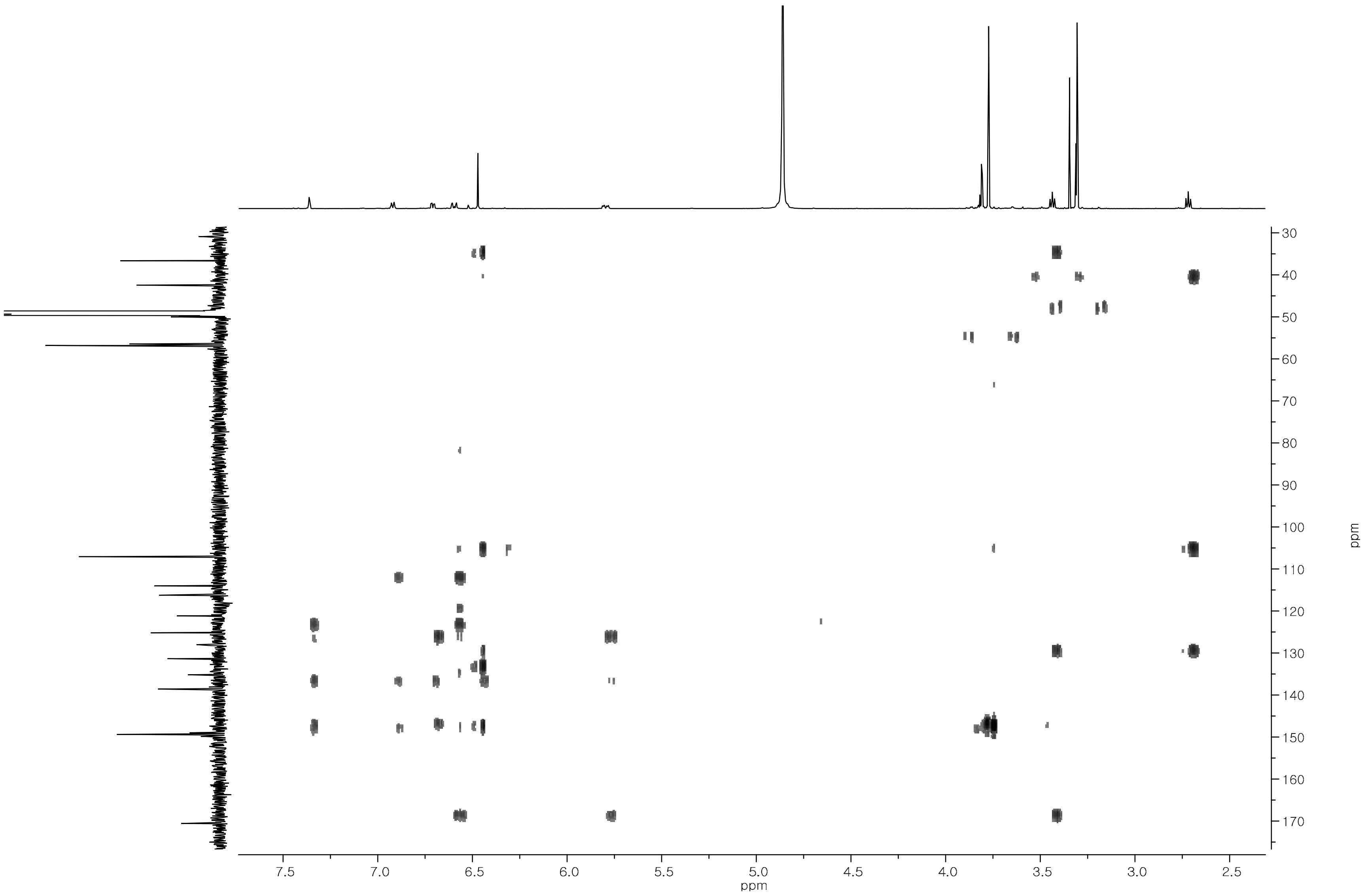

Figure S23. HMBC spectrum of **5**.

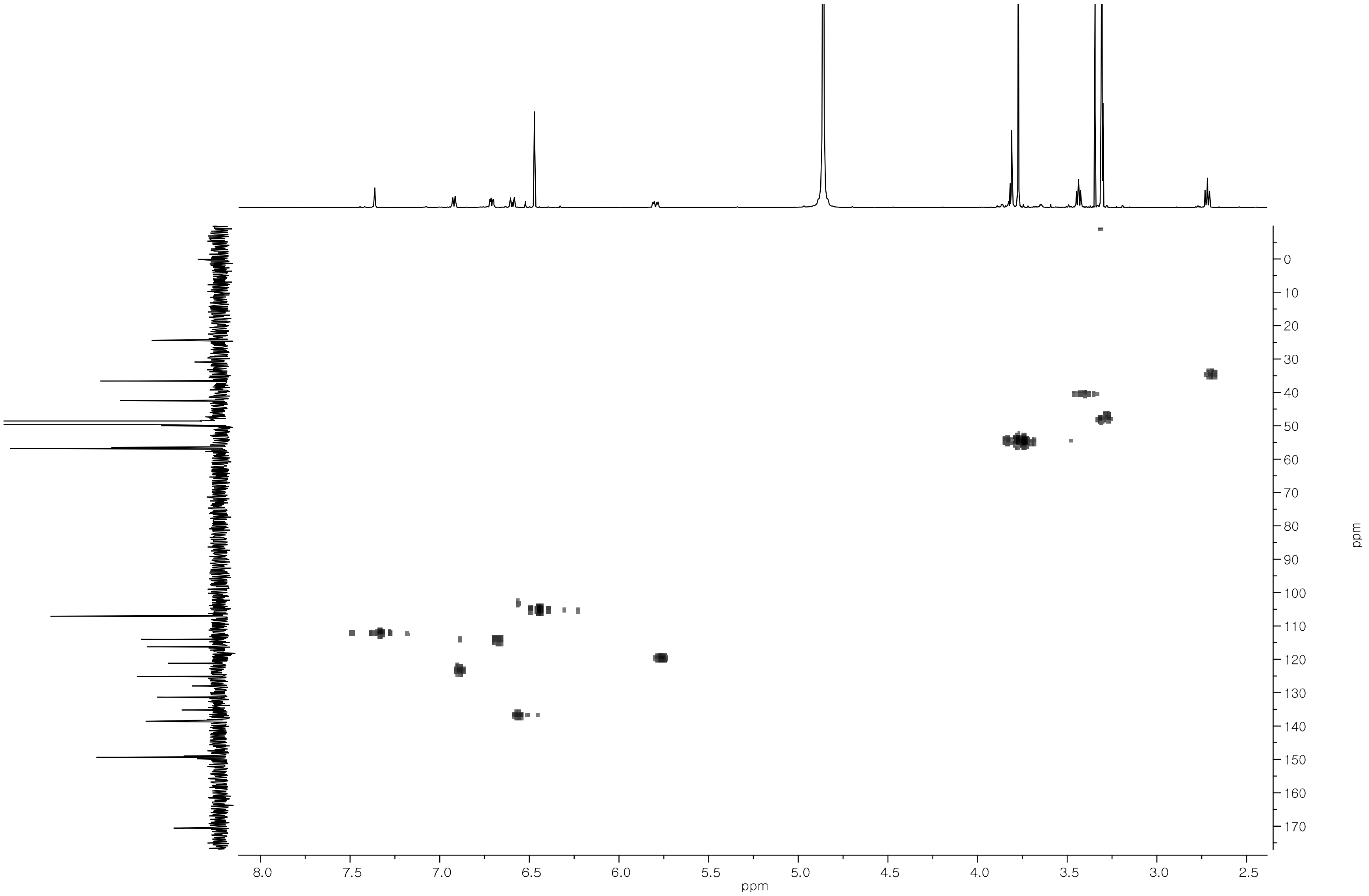

Figure S24. HMQC spectrum of **5**.

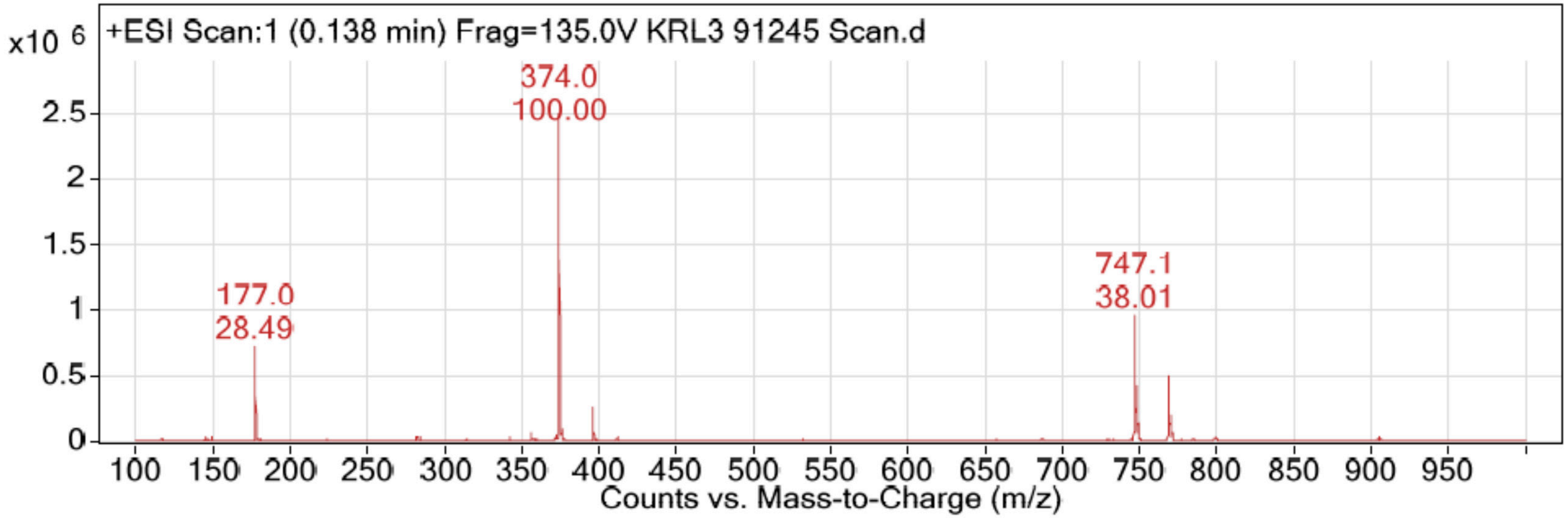

Figure S25. ESI/MS (Positive) spectrum of **6**.

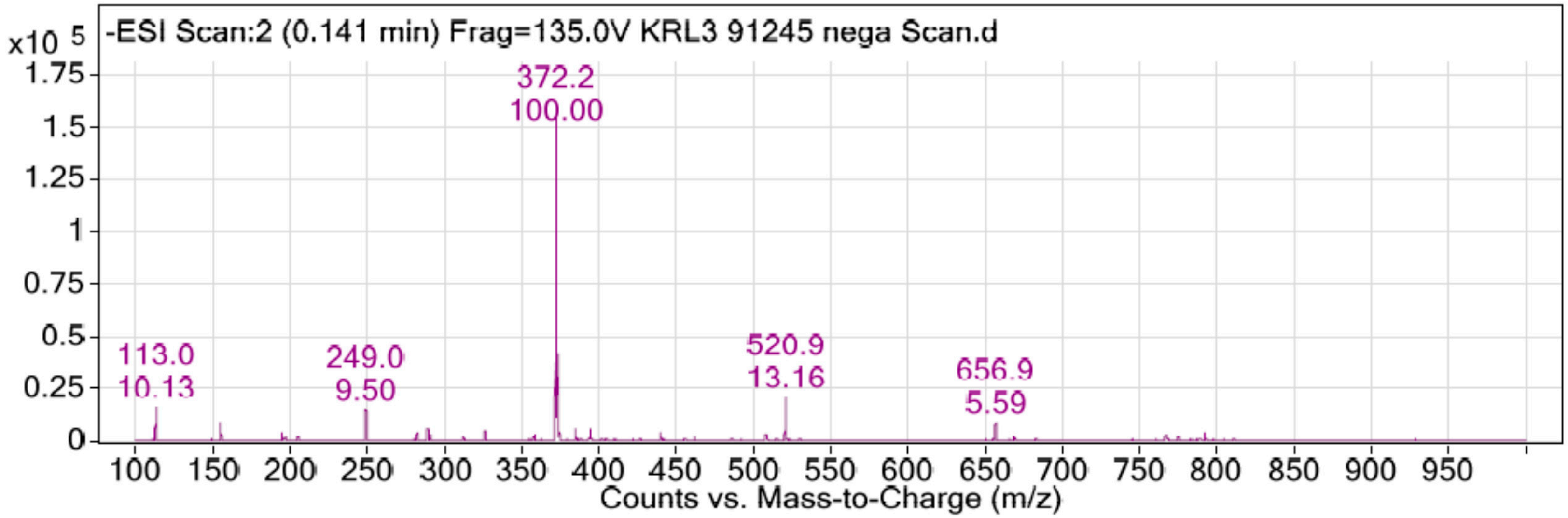

Figure S26. ESI/MS (Negative) spectrum of **6**.

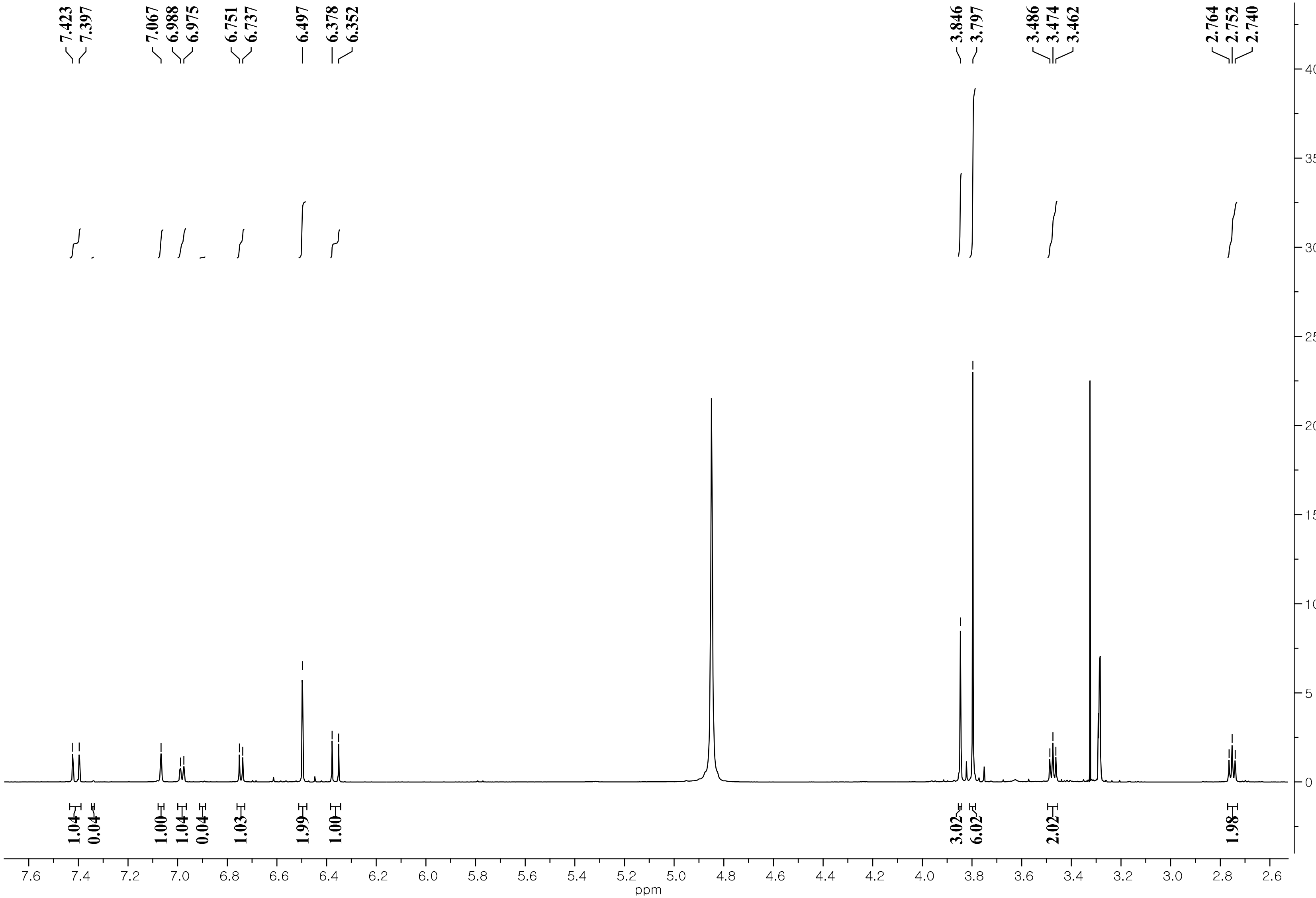

Figure S27. ^{1}H-NMR (600 MHz, CD_3OD) spectrum of **6**.

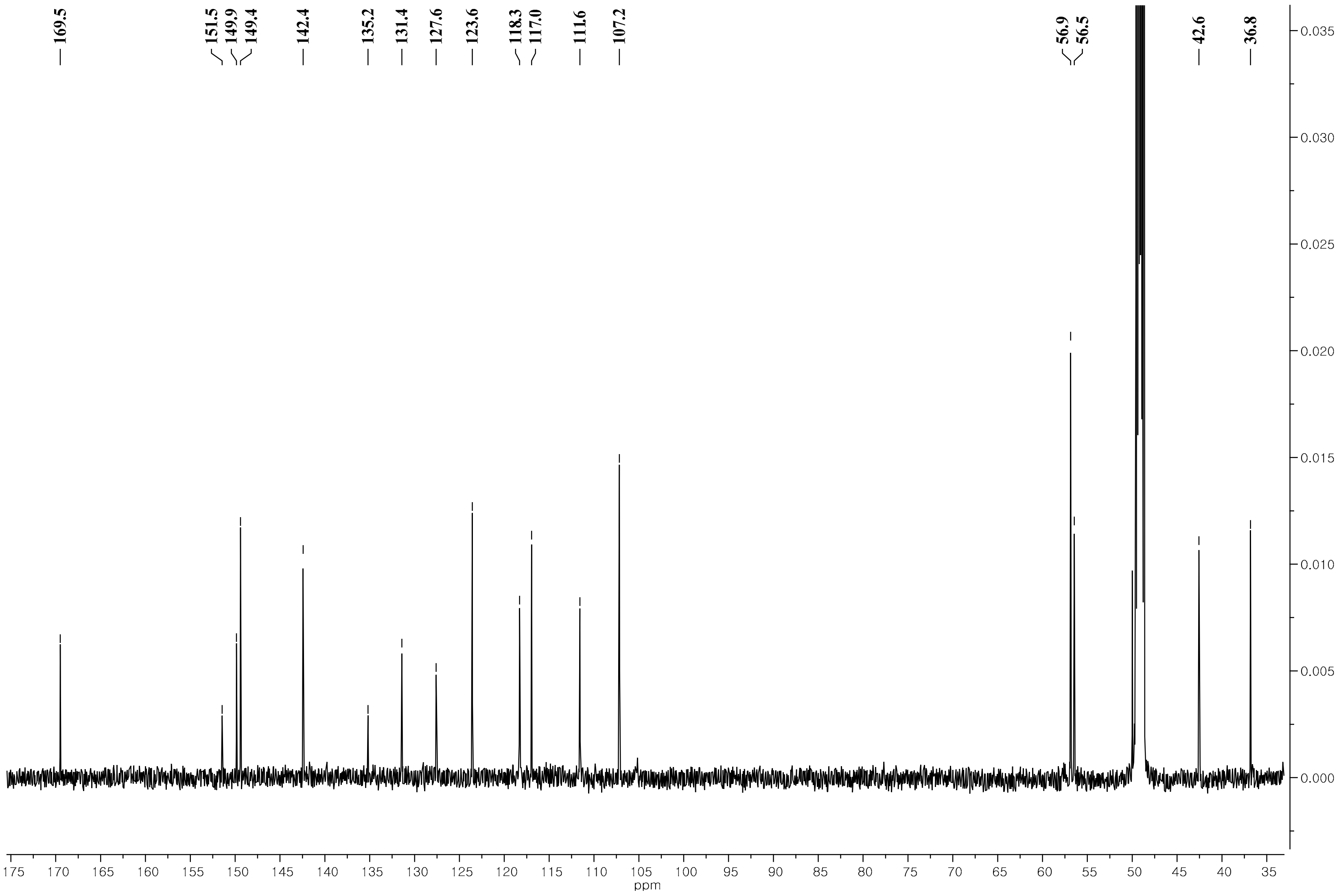

Figure S28. ^{13}C-NMR (150 MHz, CD_3OD) spectrum of **6**.

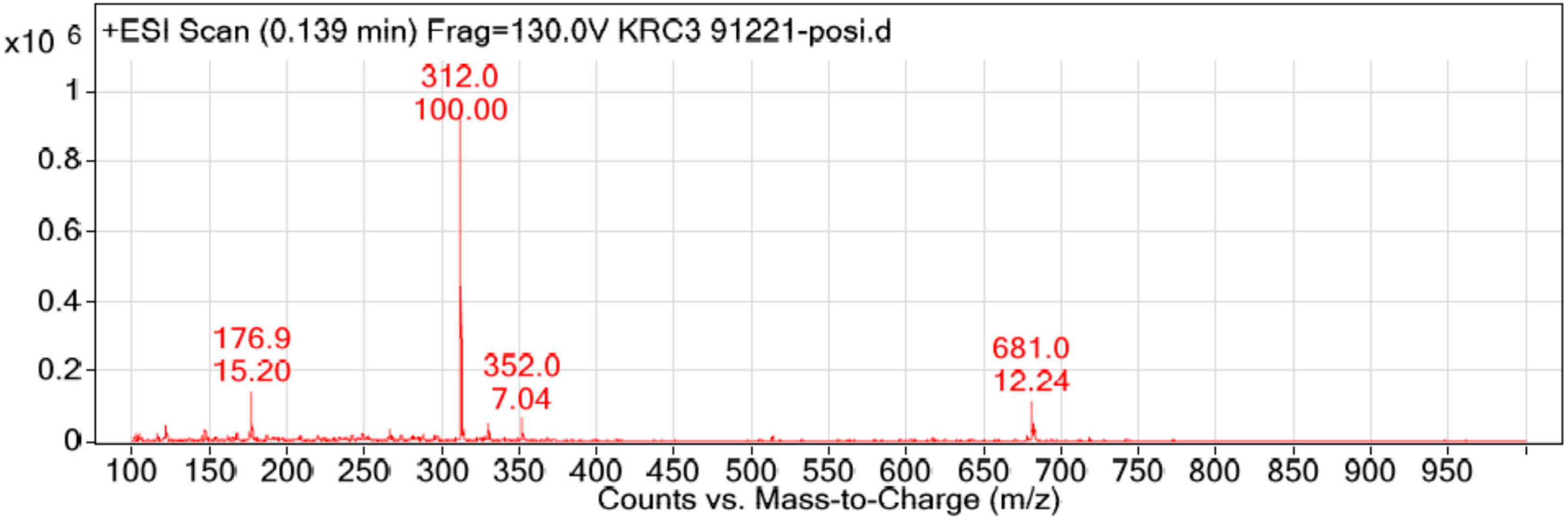

Figure S29. ESI/MS (Positive) spectrum of **7**.

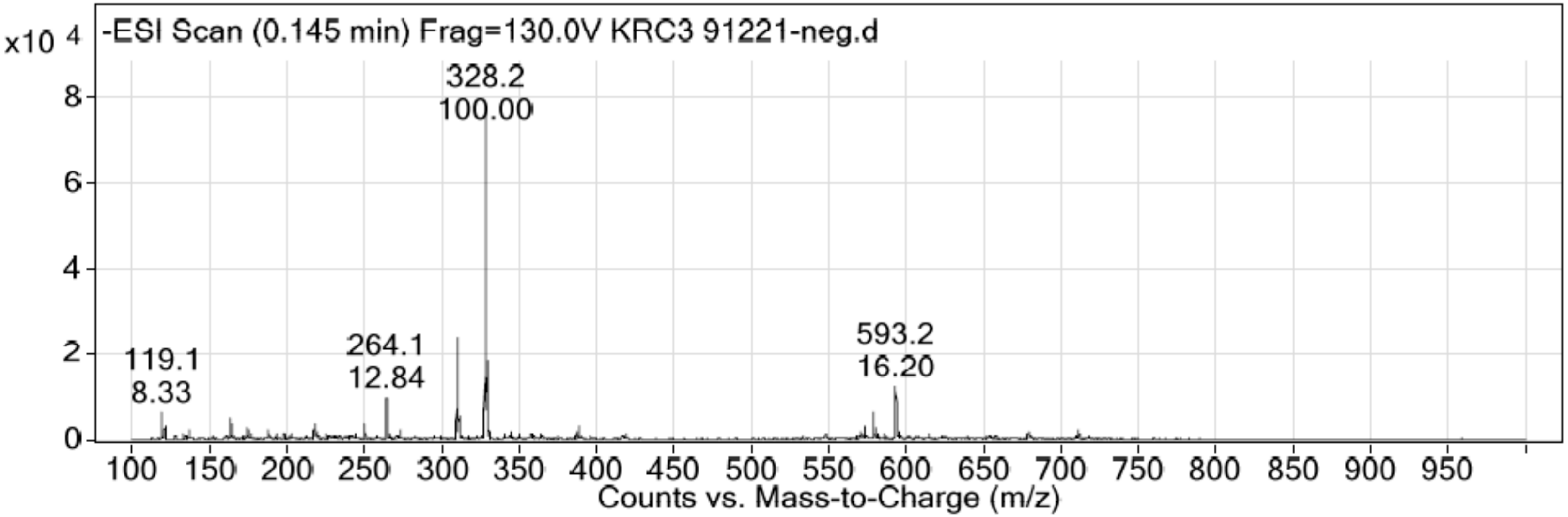

Figure S30. ESI/MS (Negative) spectrum of **7**.

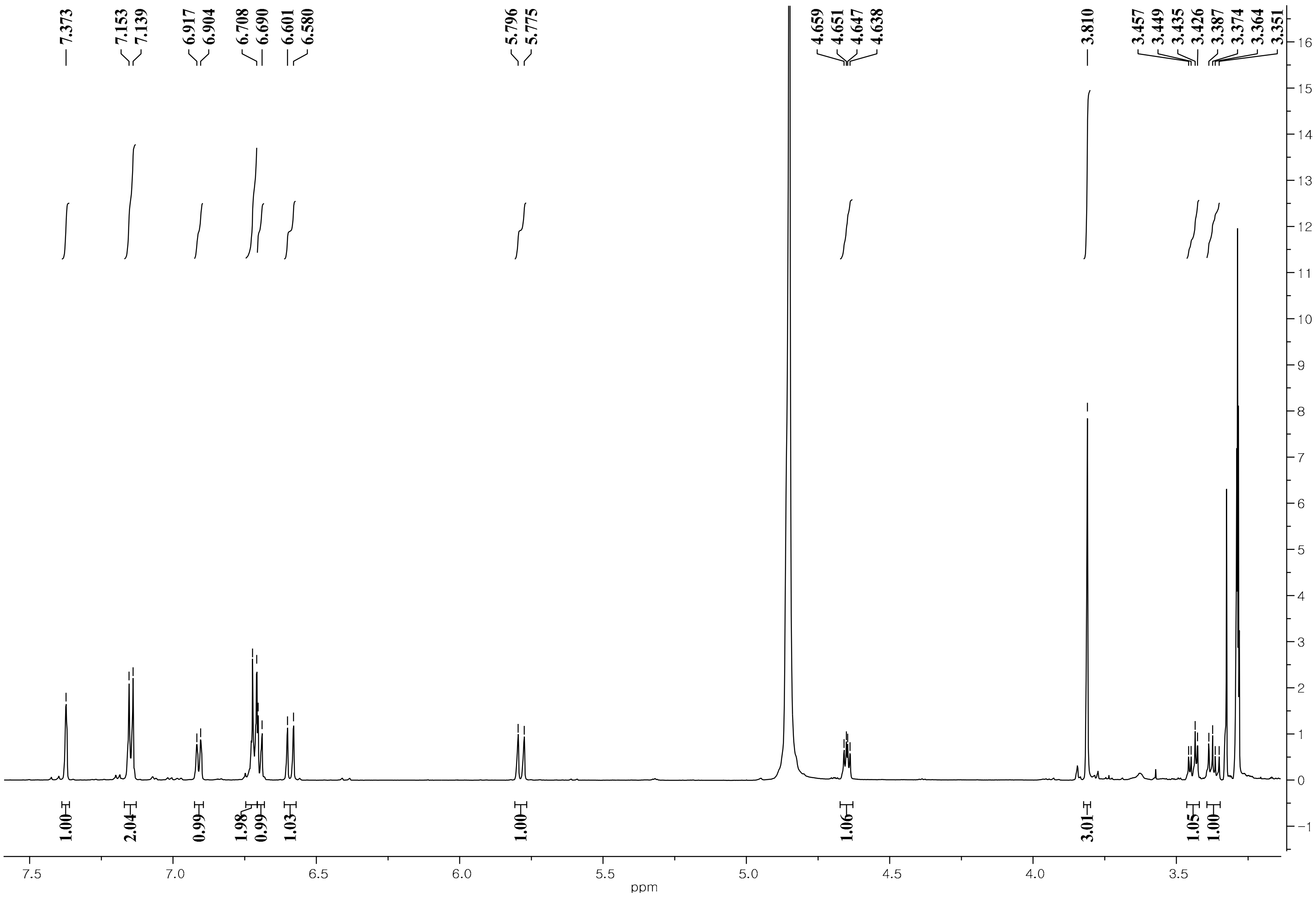

Figure S31. ^{1}H-NMR (600 MHz, CD_3OD) spectrum of **7**.

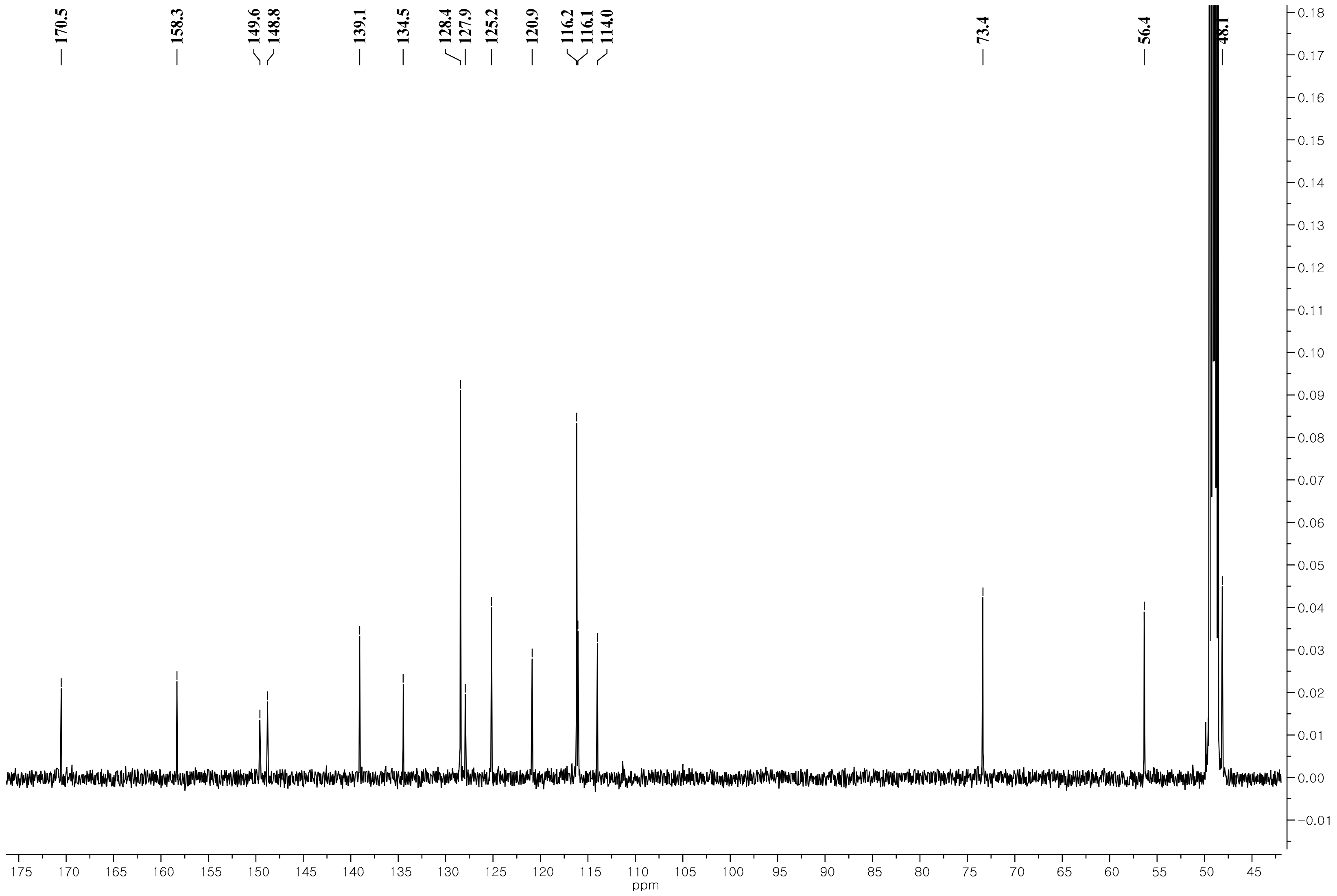

Figure S32. ^{13}C-NMR (150 MHz, CD_3OD) spectrum of **7**.

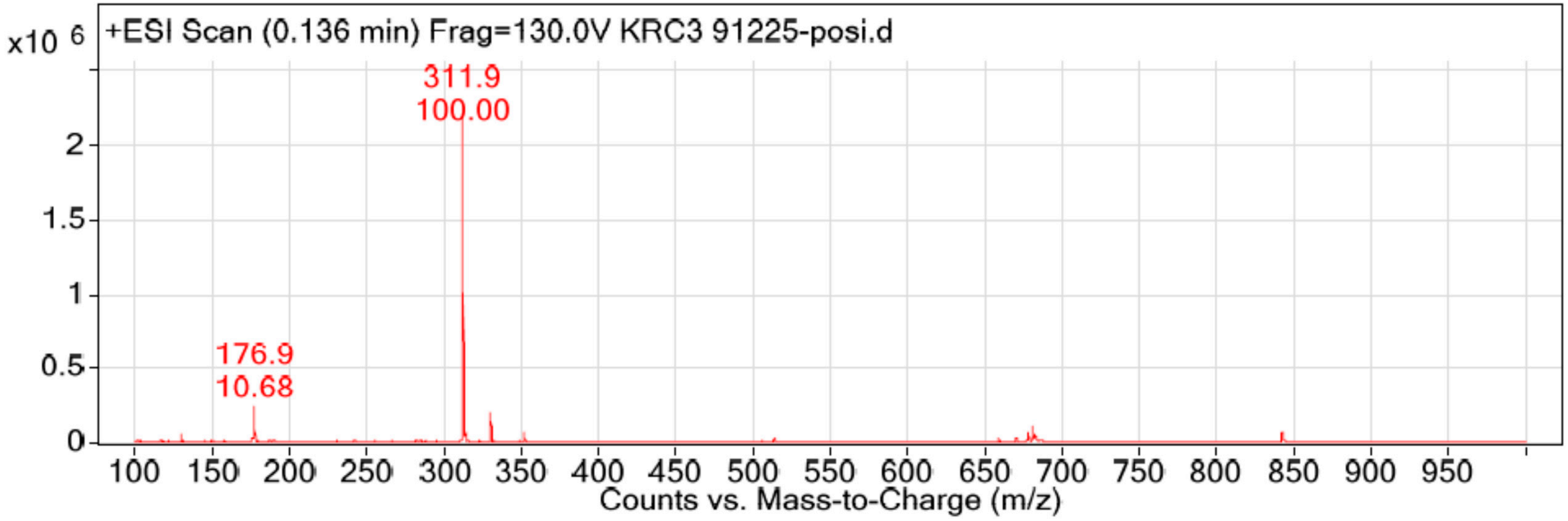

Figure S33. ESI/MS (Positive) spectrum of **8**.

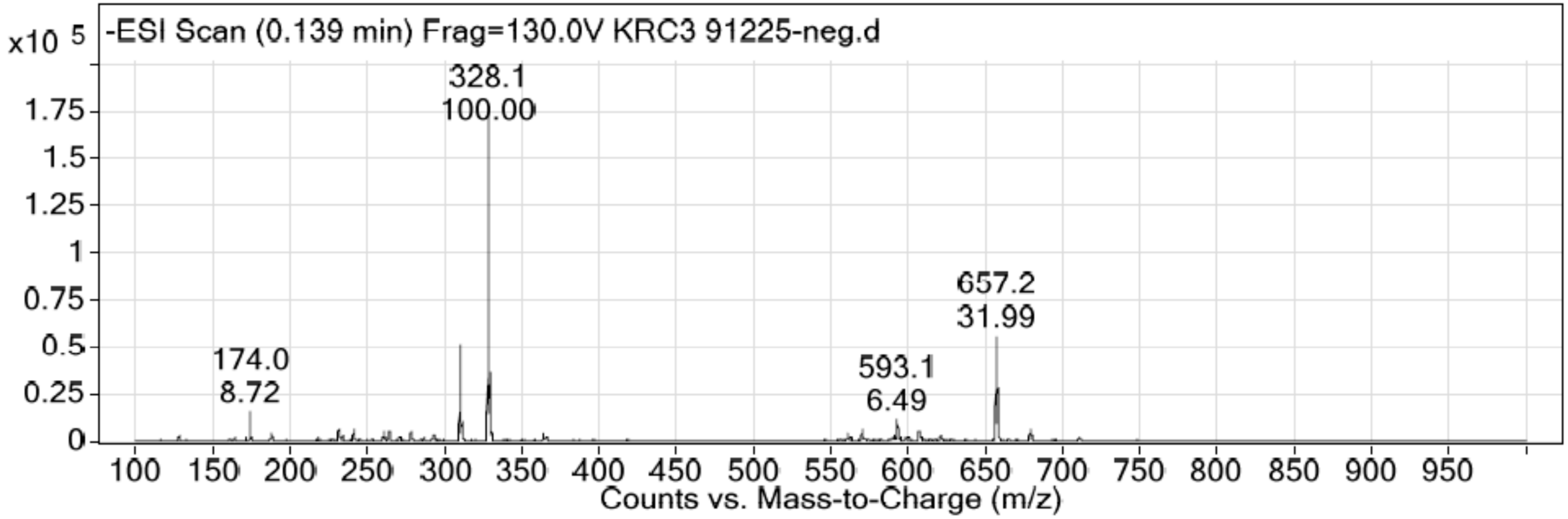

Figure S34. ESI/MS (Negative) spectrum of **8**.

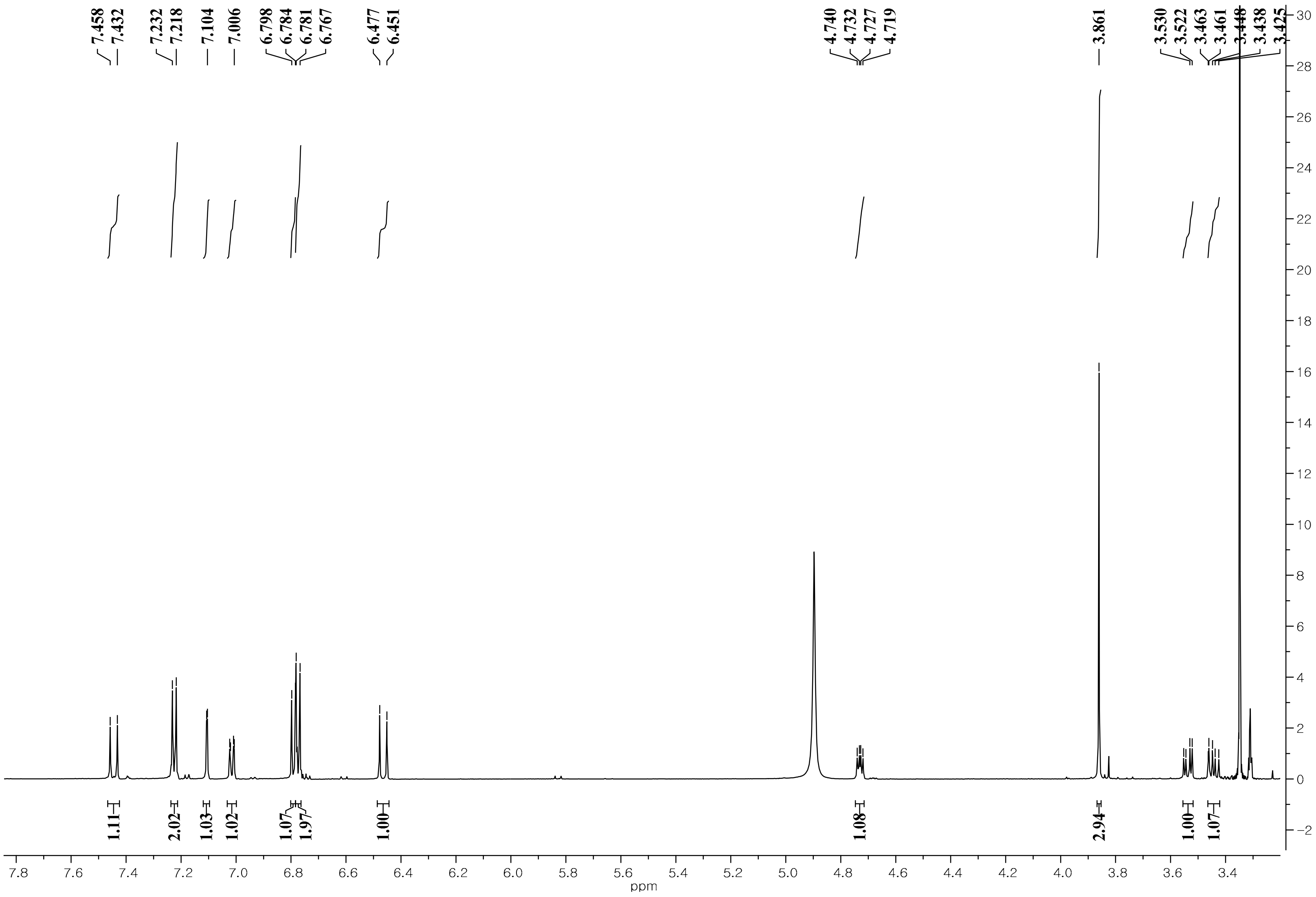

Figure S35. ^{1}H-NMR (600 MHz, CD_3OD) spectrum of **8**.

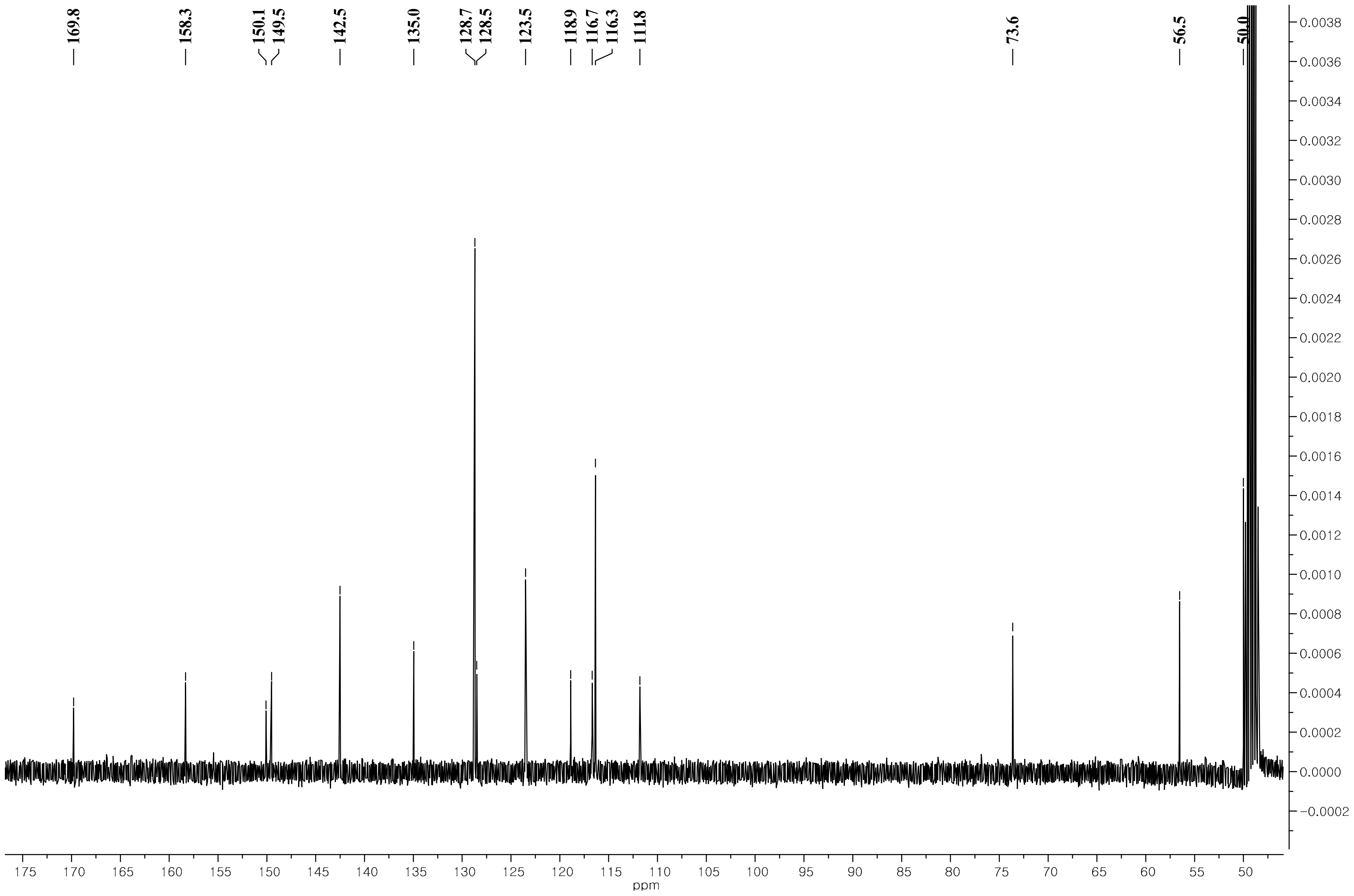

Figure S36. ^{13}C-NMR (150 MHz, CD_3OD) spectrum of **8**.

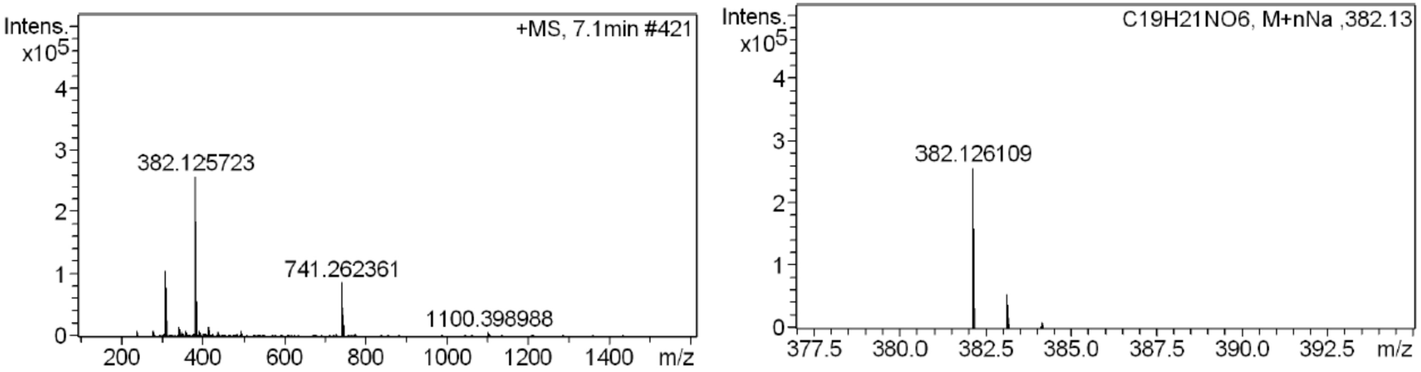

Figure S37. HRESI/MS spectrum of **9**.

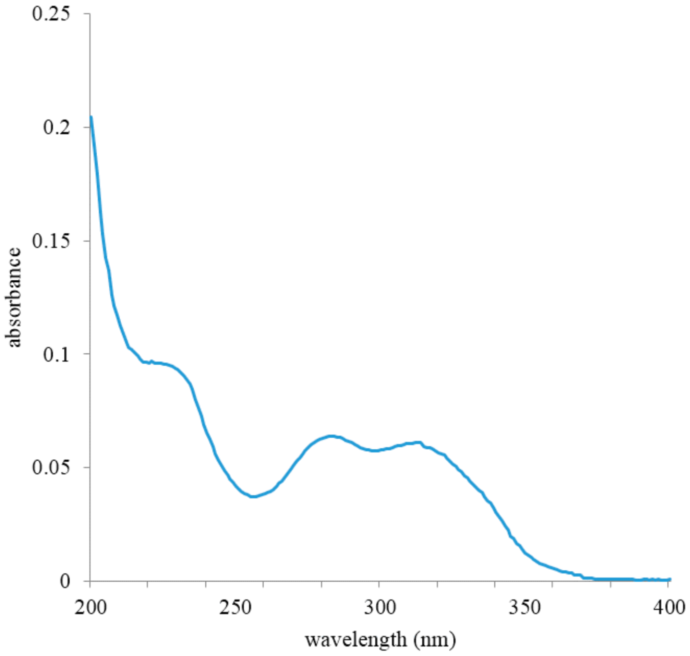

Figure S38. UV spectra of **9**.

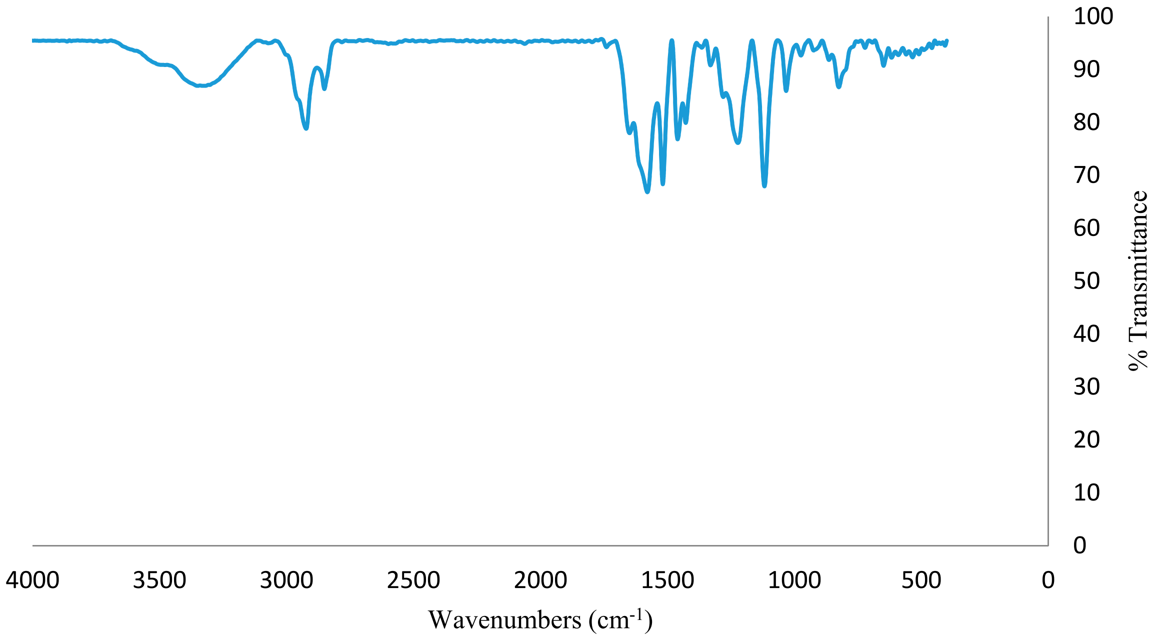

Figure S39. IR spectra of **9**.

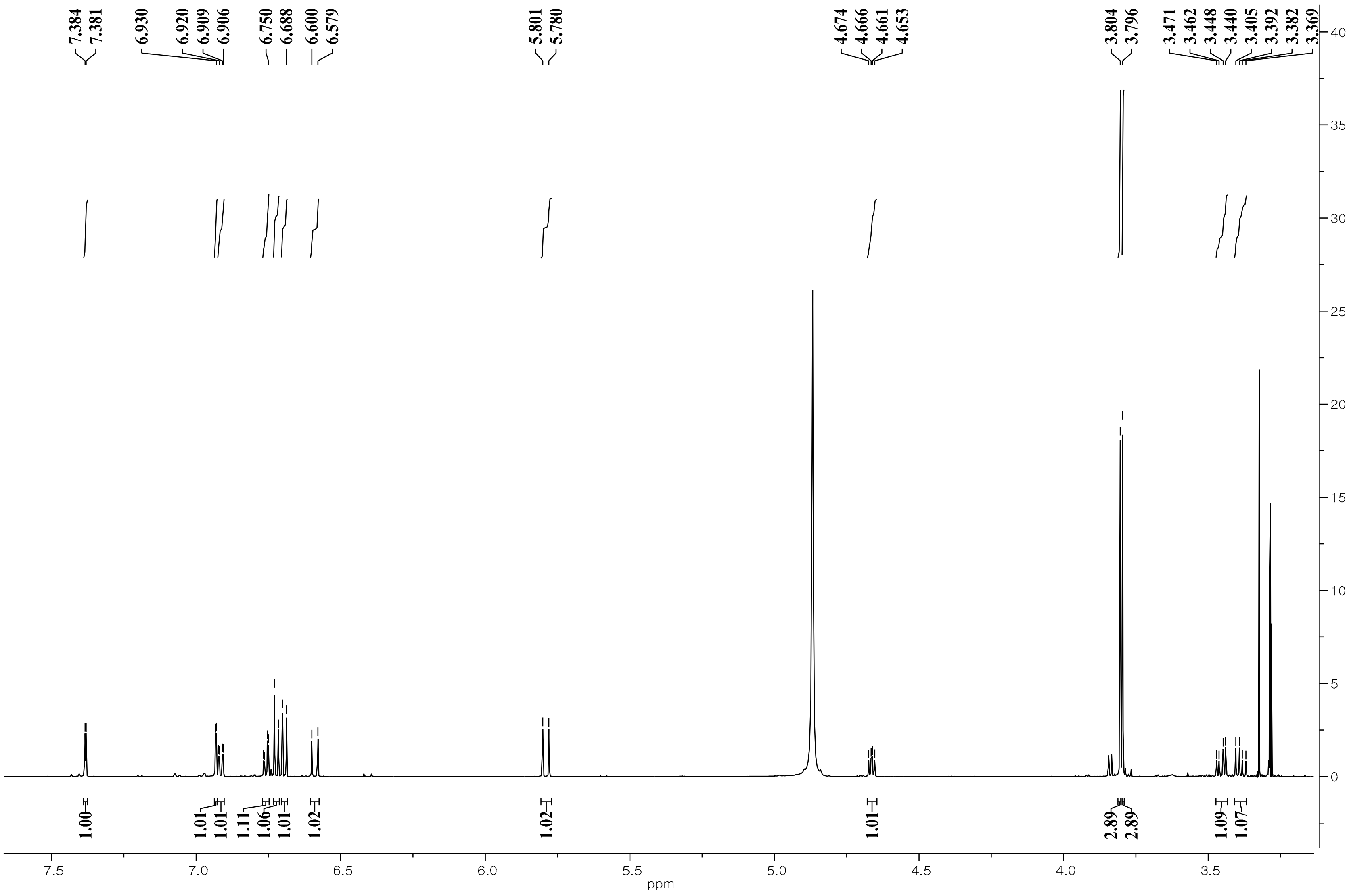

Figure S40. ^{1}H-NMR (600 MHz, CD_3OD) spectrum of **9**.

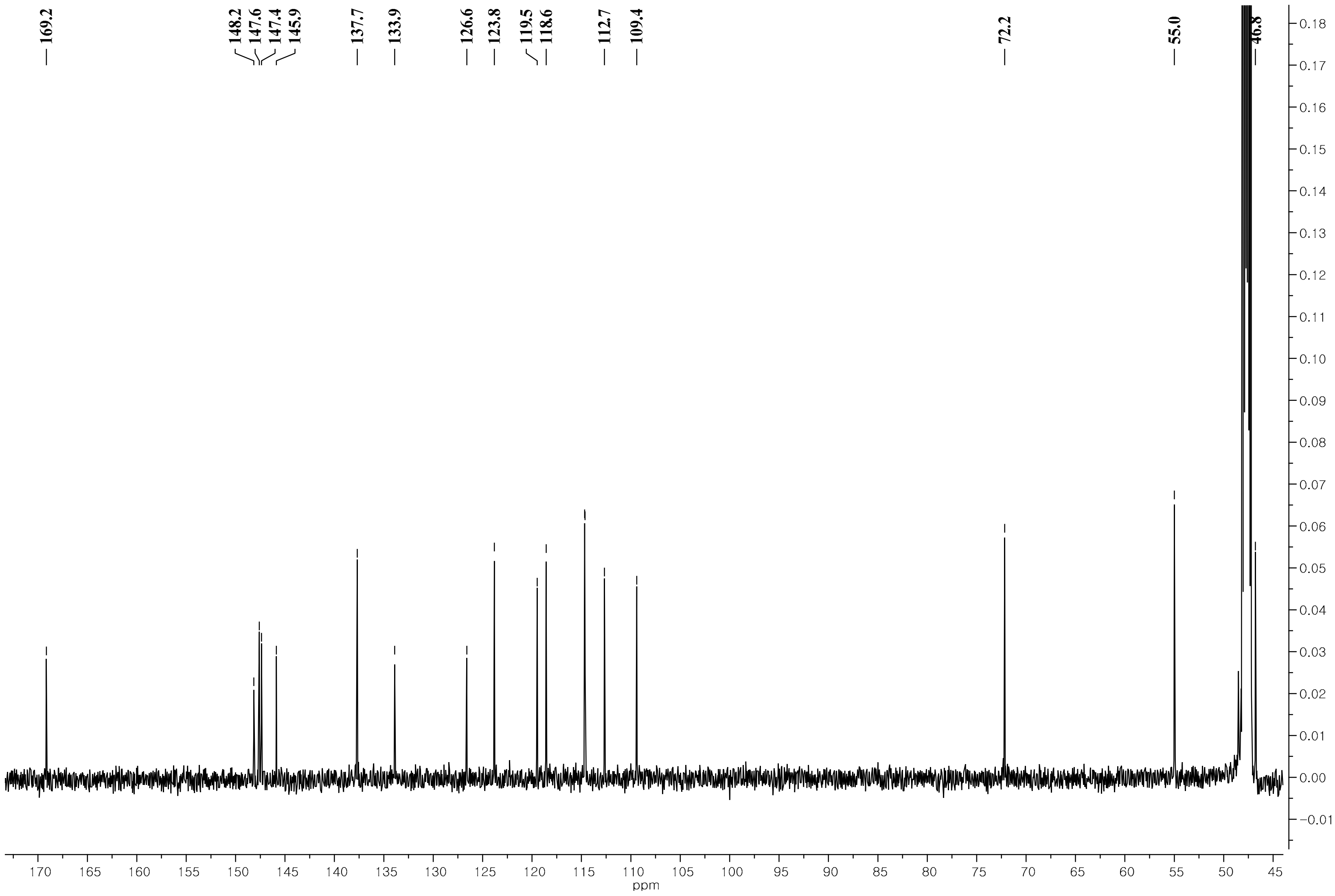

Figure S41. ^{13}C-NMR (150 MHz, CD_3OD) spectrum of **9**.

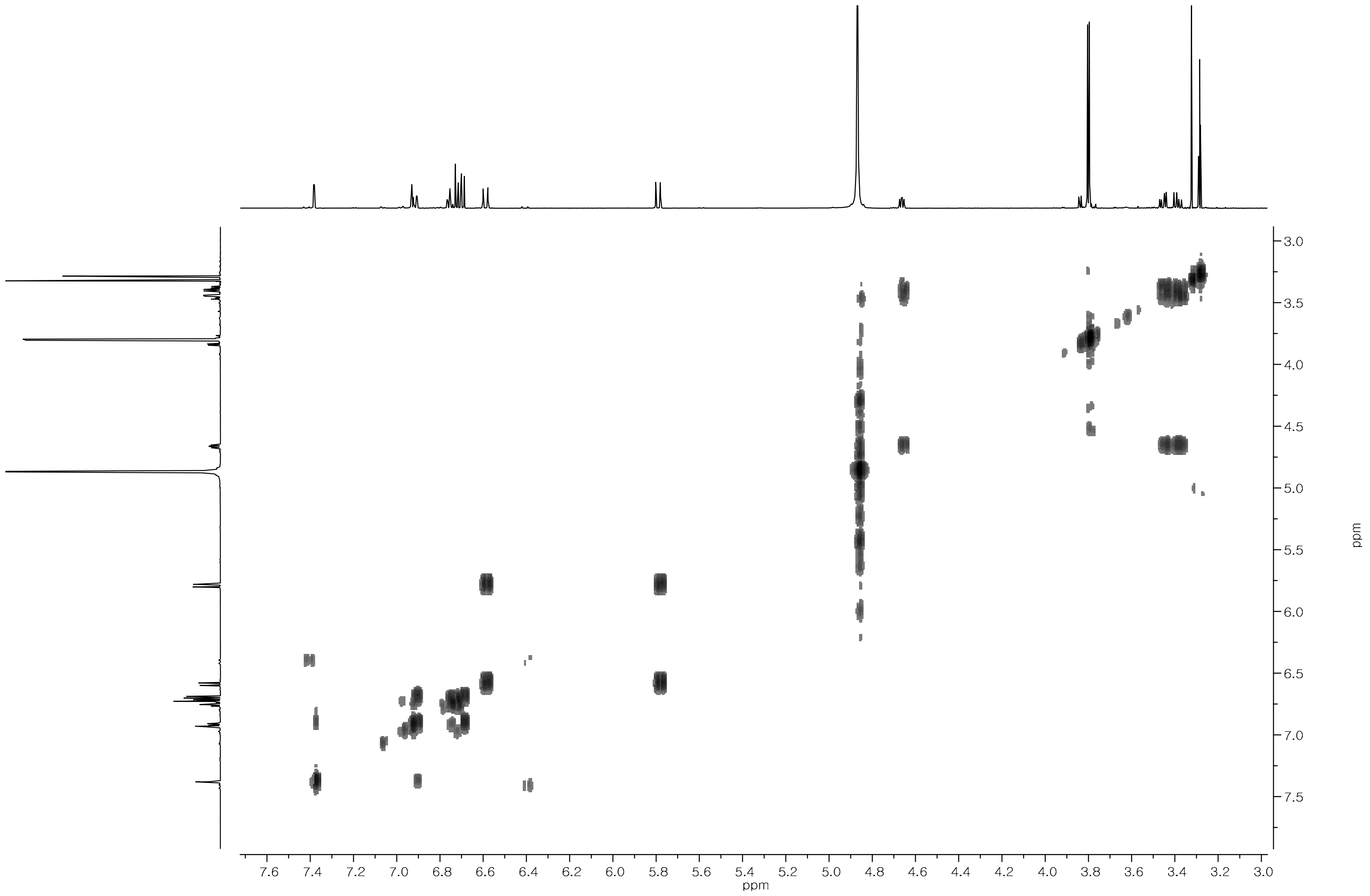

Figure S42. ^{1}H-^{1}H COSY spectrum of **9**.

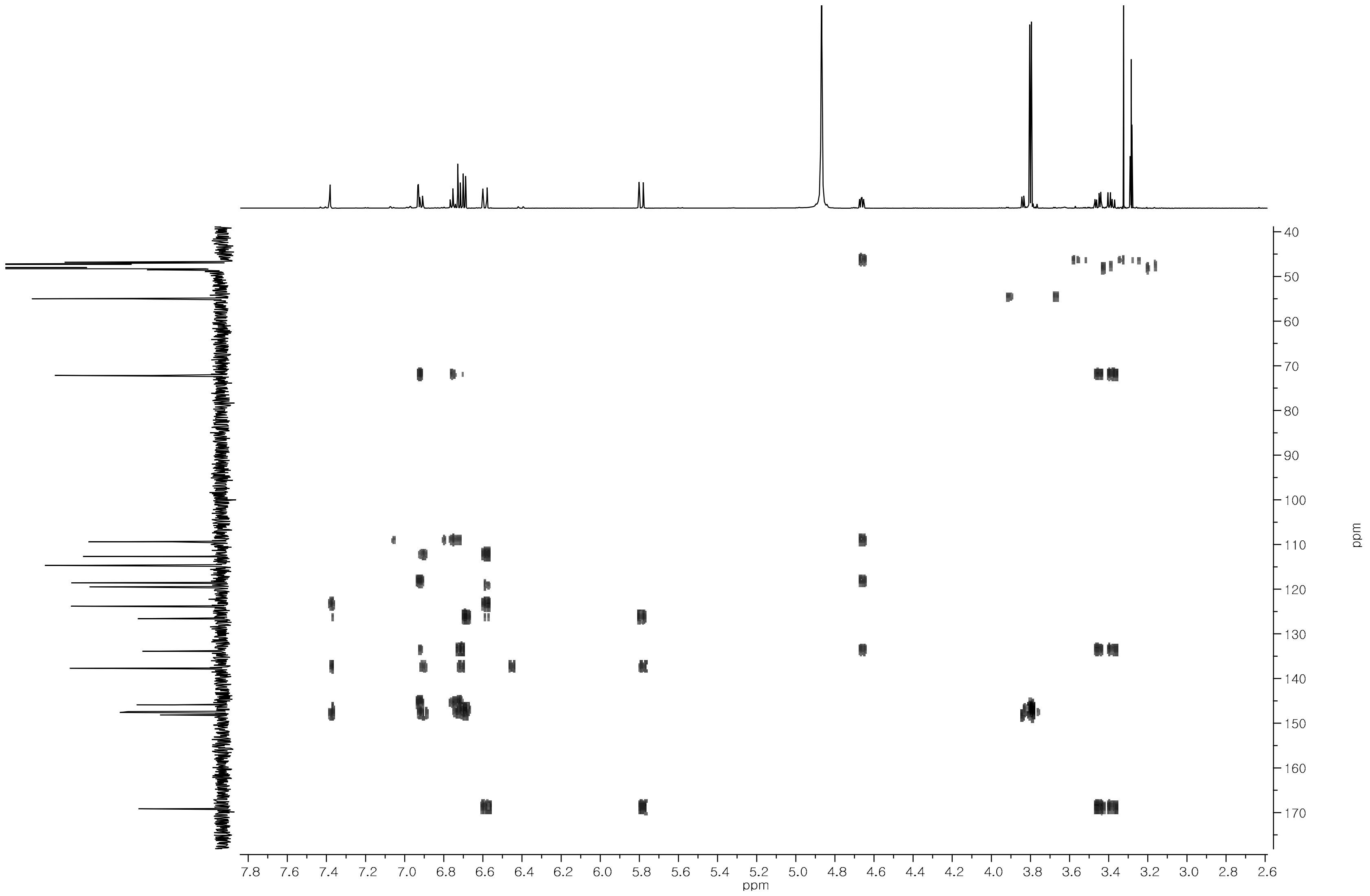

Figure S43. HMBC spectrum of **9**.

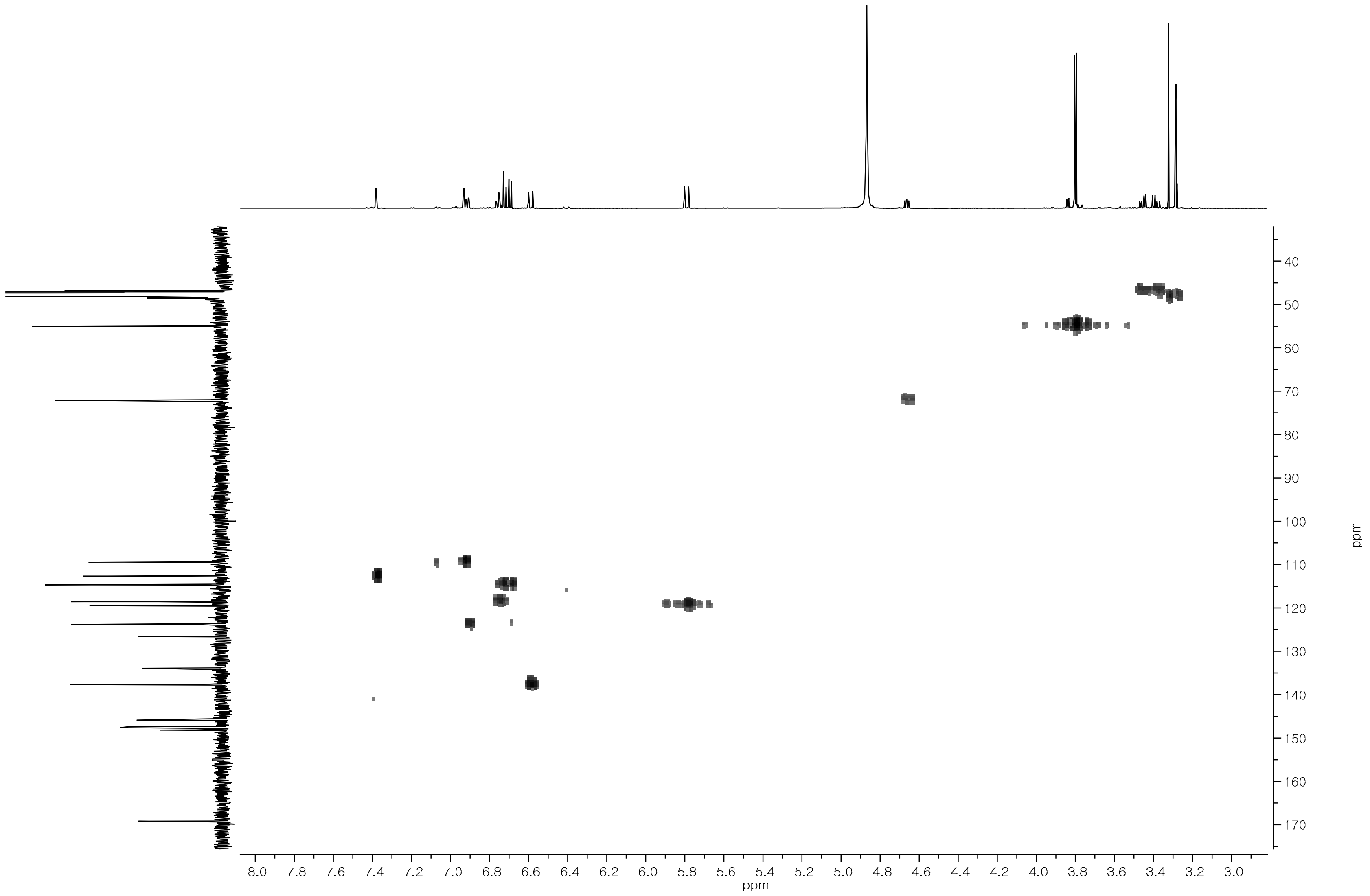

Figure S44. HMQC spectrum of **9**.

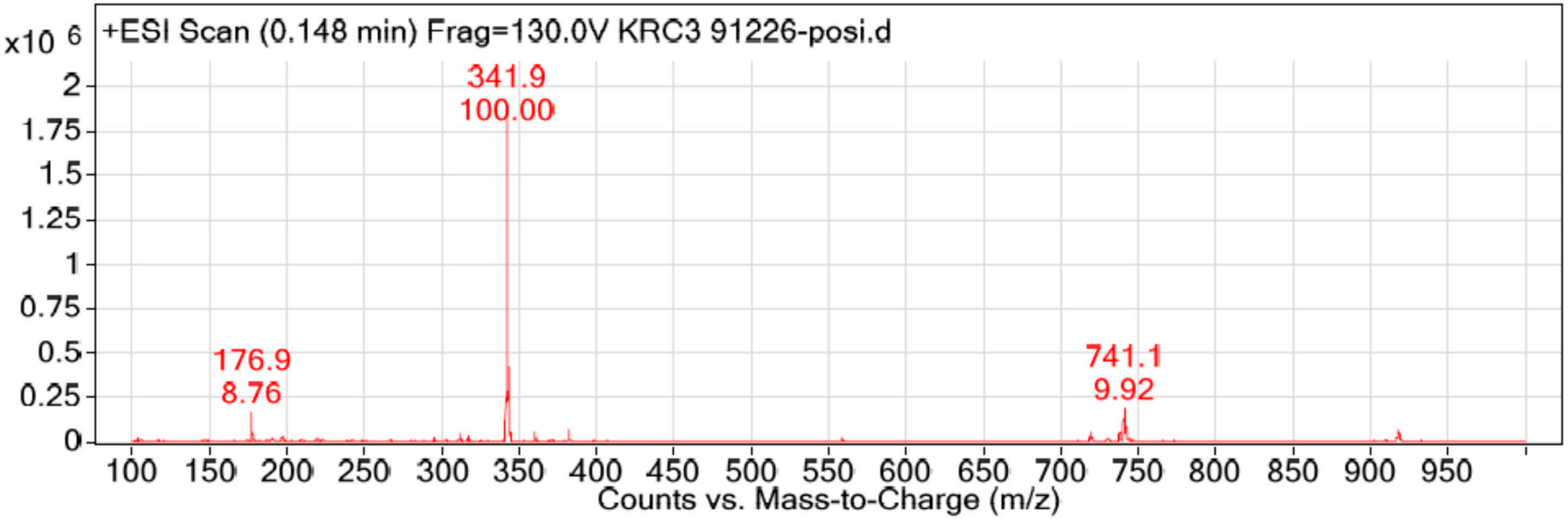

Figure S45. ESI/MS (Positive) spectrum of **10**.

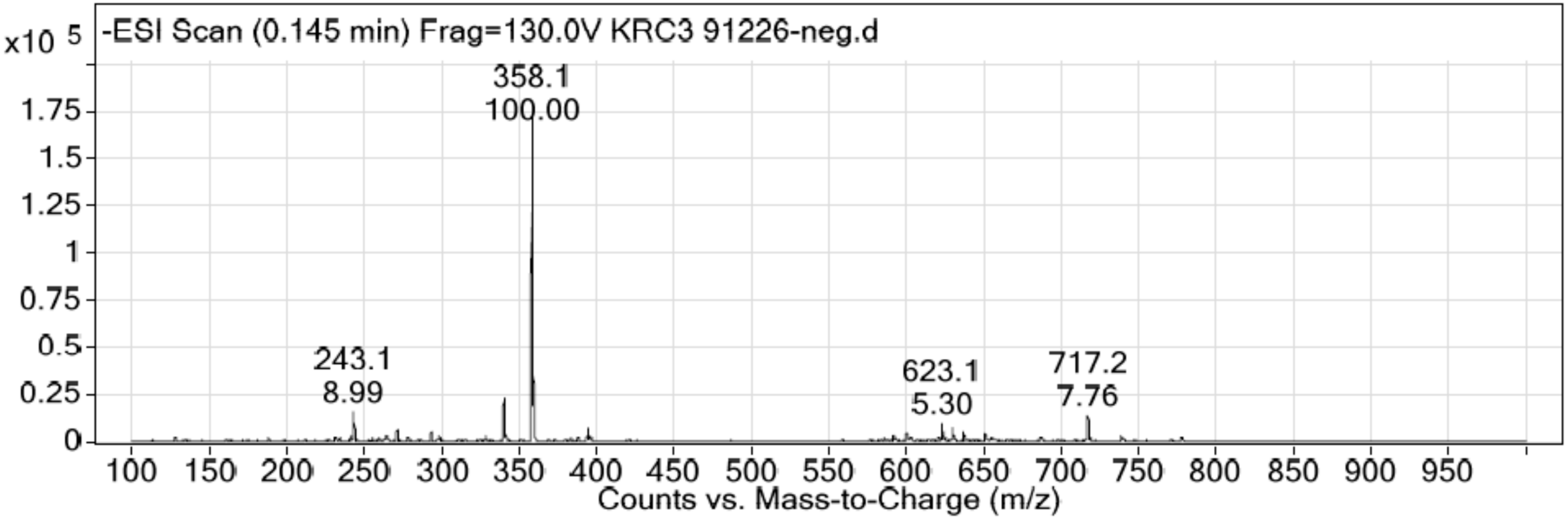

Figure S46. ESI/MS (Negative) spectrum of **10**.

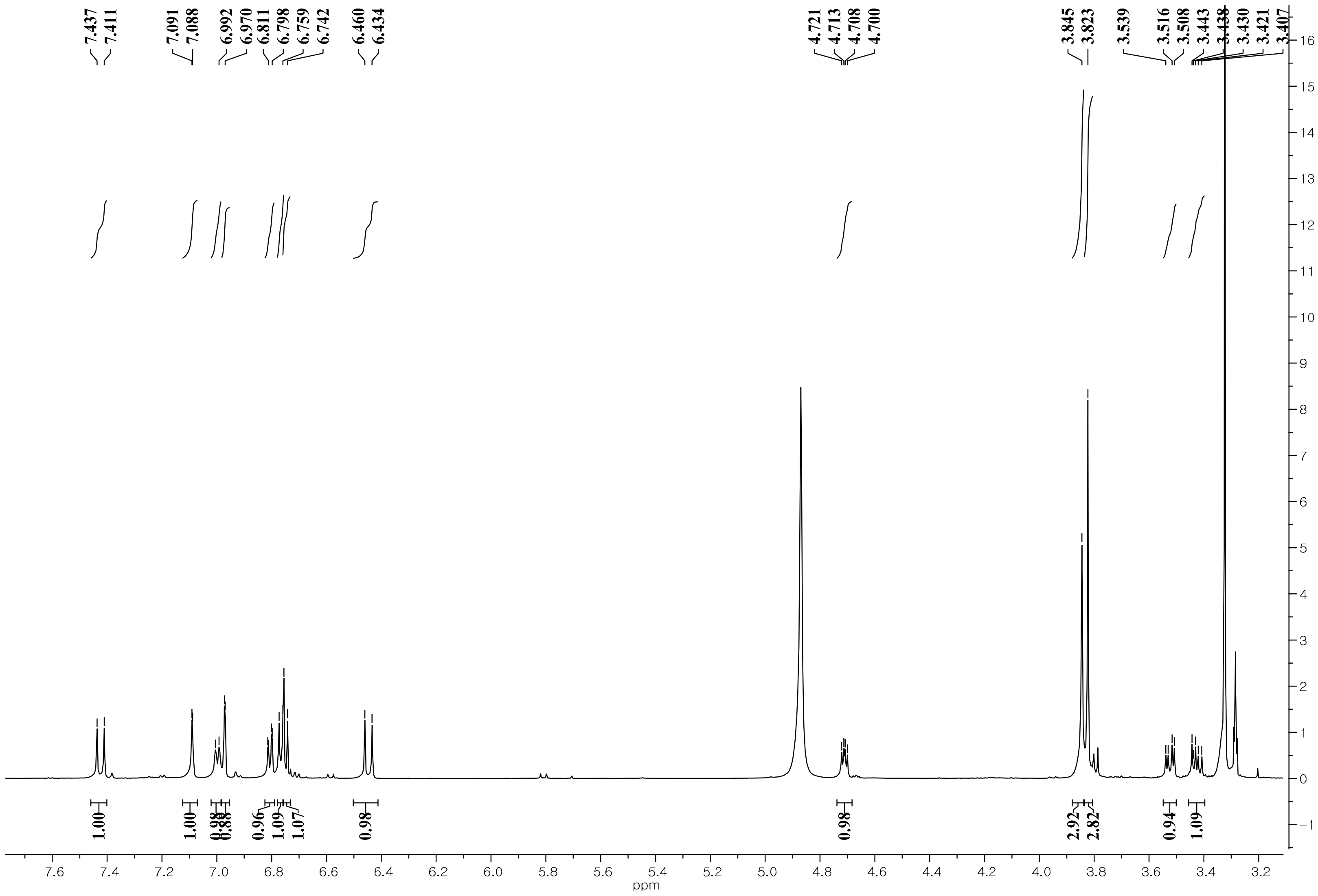

Figure S47. ^{1}H-NMR (600 MHz, CD_3OD) spectrum of **10**.

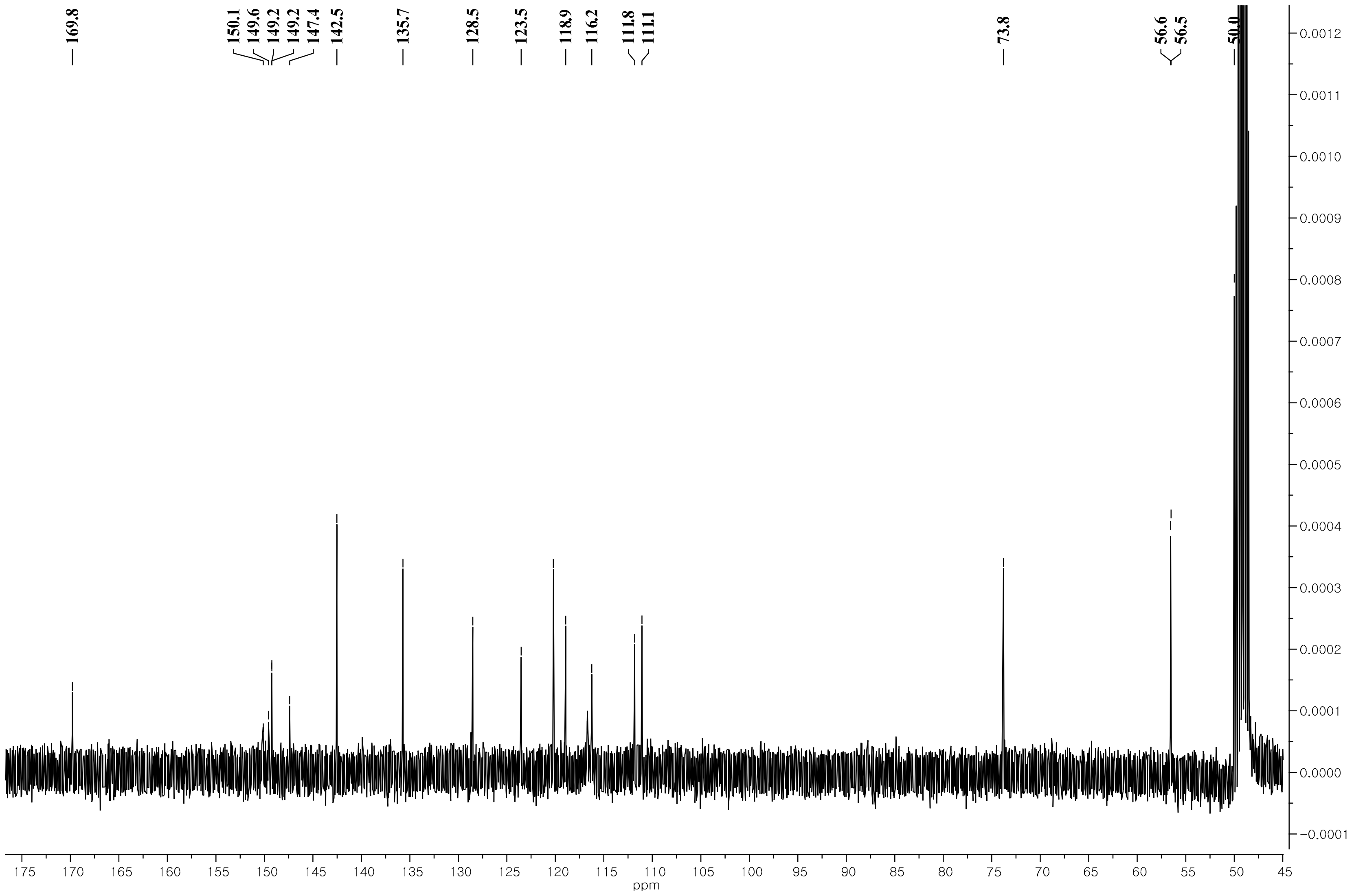

Figure S48. ^{13}C-NMR (150 MHz, CD_3OD) spectrum of **10**.

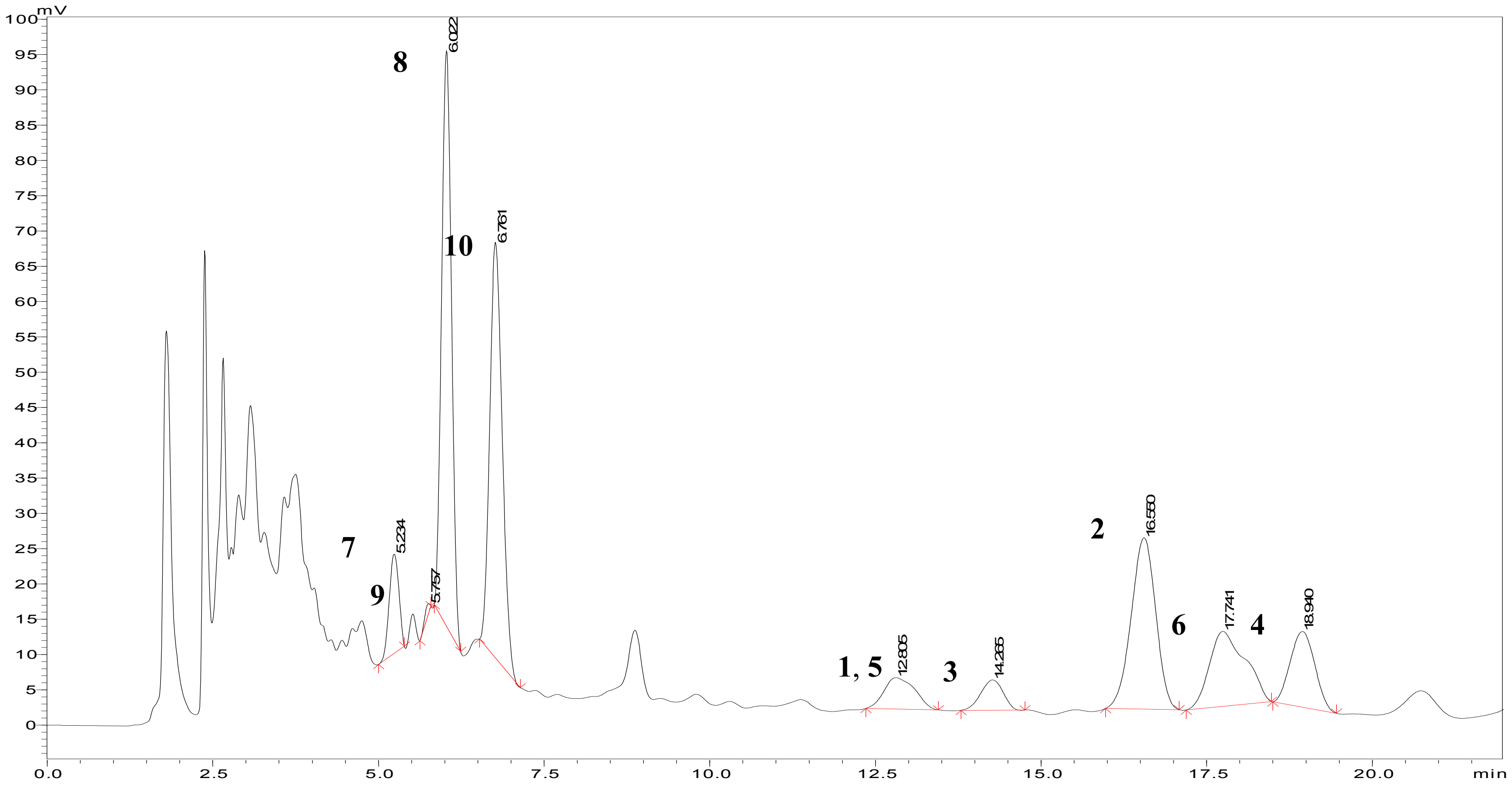

Figure S49. HPLC chromatogram of *P. oleracea* (dry powder*)* which refined by Silica-SPE (eluted with a Hexane:EtOAc = 7:3).

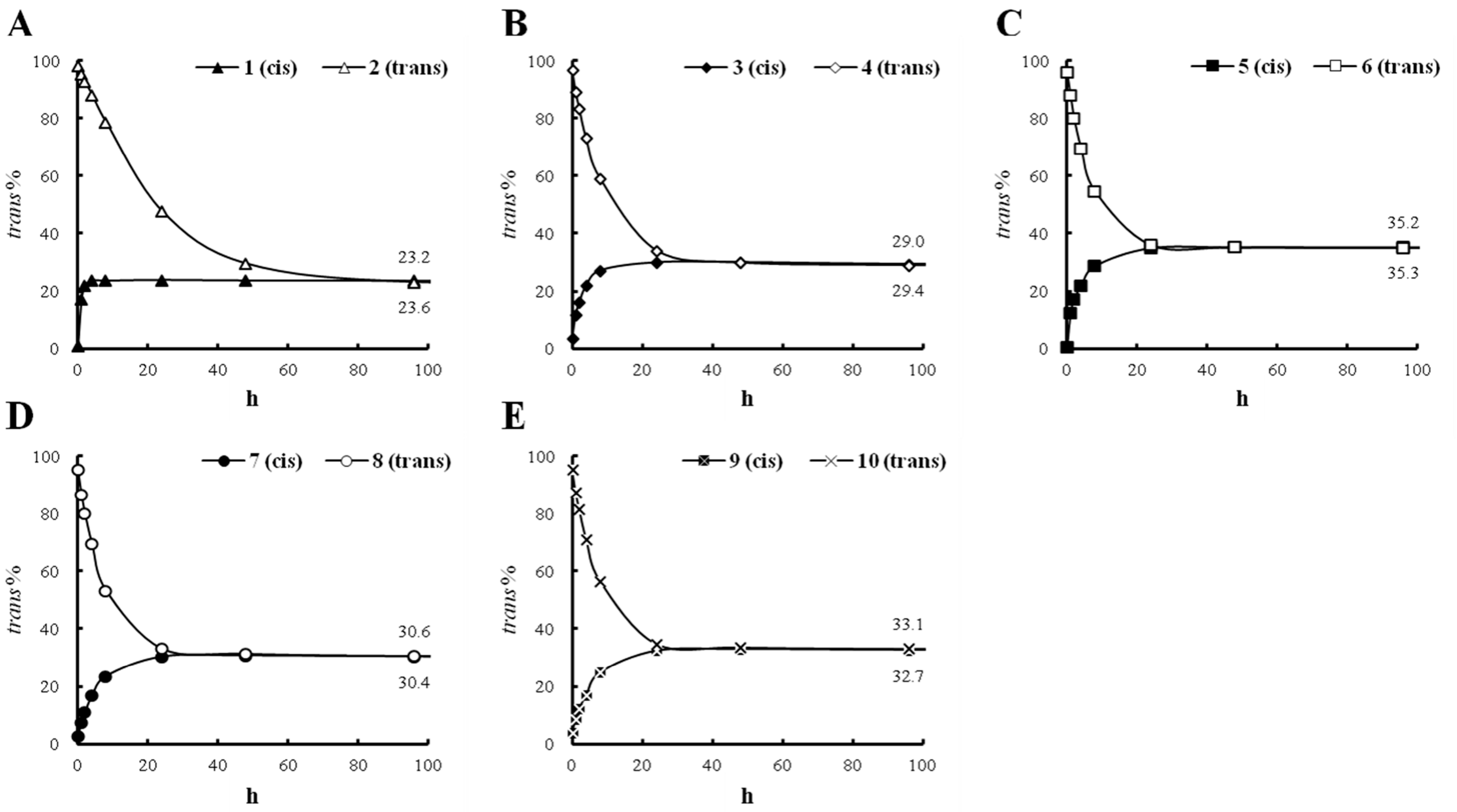

Figure S50. Conversion rates and equilibrium ratio of Compound **1** and **2** (**A**); Compound **3** and **4** (**B**); Compound **5** and **6** (**C**); Compound **7** and **8** (**D**); Compound **9** and **10** (**E**).

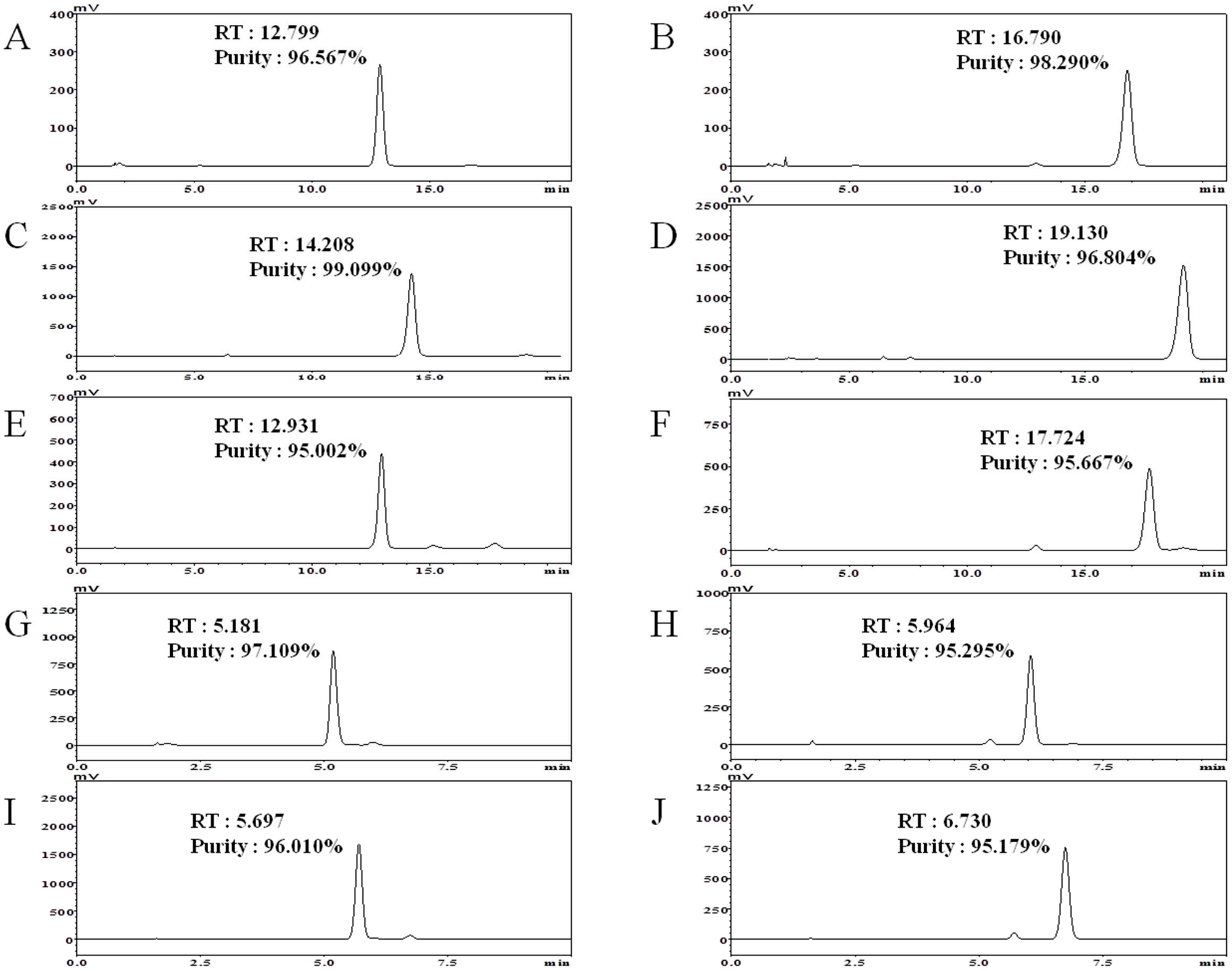

Figure S51. The HPLC chromatograms of **1** (**A**); **2** (**B**); **3** (**C**); **4** (**D**); **5** (**E**); **6** (**F**); **7** (**G**); **8** (**H**); **9** (**I**) and **10** (**J**).

Table S1. Conversion rates of *cis* and *trans*- feruloyl amides (**1**–**10**).

Compound	t_R (min) [a]	0 h	1 h	2 h	4 h	8 h	24 h	48 h	96 h
N-*cis*-feruloyl tyramine (**1**)	12.8	0.9 [b]	17.2	21.8	23.8	23.7	23.9	23.9	23.6
N-*trans*-feruloyl tyramine (**2**)	16.7	98.3	95.2	92.6	88.1	78.6	47.7	29.6	23.2
N-*cis*-feruloyl-3′-methoxytyrammine (**3**)	14.3	3.4	11.8	16.1	22.0	27.2	30.0	30.0	29.4
N-*trans*-feruloyl-3′-methoxytyrammine (**4**)	19.2	96.8	89.2	83.4	73.0	58.9	34.1	29.8	29.0
N-*cis*-hibiscuamide (**5**)	12.9	5.0	12.6	17.3	22.1	28.9	35.1	35.3	35.0
N-*trans*-hibiscuamide (**6**)	17.8	95.8	87.8	80.1	69.6	54.6	36.1	34.3	34.2
(7′*S*)-*N*-*cis*-feruloyl octopamine (**7**)	5.2	2.9	7.4	10.9	16.8	23.5	30.3	30.7	30.4
(7′*S*)-*N*-*trans*-feruloyl octopamine (**8**)	6.0	95.3	86.6	80.0	69.5	53.3	33.1	31.3	30.6
(7′*S*)-*N*-*cis*-feruloyl normetamephrine (**9**)	5.7	4.0	8.5	12.2	16.8	24.8	32.3	32.9	32.7
(7′*S*)-*N*-*trans*-feruloyl normetamephrine (**10**)	6.7	95.2	87.3	81.4	71.1	56.5	34.8	33.6	33.1

[a] Retention time of the compound in HPLC analysis; [b] Peak area normalization method to calculate the percentage of *trans*.
